# A systems immunology approach to investigate cytokine responses to viruses and bacteria and their association with disease

**DOI:** 10.1038/s41598-022-16509-4

**Published:** 2022-08-05

**Authors:** Lijing Lin, John A. Curtin, Eteri Regis, Aurica Hirsman, Rebecca Howard, Mauro Tutino, Michael R. Edwards, Mattia Prosperi, Angela Simpson, Magnus Rattray, Adnan Custovic, Sebastian L. Johnston

**Affiliations:** 1 Faculty of Biology, Medicine and Health, University of Manchester, Manchester, UK; 2 National Heart and Lung Institute, Asthma UK Centre in Allergic Mechanisms of Asthma, Imperial College London, London, UK; 3 Department of Epidemiology, College of Public Health and Health Professions & College of Medicine, University of Florida, Florida, 32611 USA; 4 Department of Transfusion Medicine and Haemostaseology, Erlangen University Hospital, 91054 Erlangen, Germany

**Keywords:** Computational biology and bioinformatics, Immunology, Cytokines, Respiratory tract diseases

## Abstract

Patterns of human immune responses to viruses and bacteria and how this impacts risk of infections or onset/exacerbation of chronic respiratory diseases are poorly understood. In a population-based birth cohort, we measured peripheral blood mononuclear cell responses (28 cytokines) to respiratory viruses and bacteria, Toll-like receptor ligands and phytohemagglutinin, in 307 children. Cytokine responses were highly variable with > 1000-fold differences between children. Machine learning revealed clear distinction between virus-associated and bacteria-associated stimuli. Cytokines clustered into three functional groups (anti-viral, pro-inflammatory and T-cell derived). To investigate mechanisms potentially explaining such variable responses, we investigated cytokine Quantitative Trait Loci (cQTLs) of IL-6 responses to bacteria and identified nine (eight novel) loci. Our integrative approach describing stimuli, cytokines and children as variables revealed robust immunologically and microbiologically plausible clustering, providing a framework for a greater understanding of host-responses to infection, including novel genetic associations with respiratory disease.

## Introduction

Viruses and bacteria are major causes of respiratory morbidity, including upper and lower respiratory tract infections (RTIs). Patients with obstructive lung diseases such as asthma and chronic obstructive pulmonary disease (COPD) have a substantially increased risk of RTIs, and in this patient group, virus-induced exacerbations^1–4^ and invasive pneumococcal disease^5–7^ are responsible for substantial morbidity and mortality. Virus infections (in particular those with rhinoviruses [RV]), may be causally linked with asthma development^8,9^. However, the immune mechanisms of the susceptibility to infection, and its relationship with the pathogenesis of obstructive lung diseases, and predisposition to exacerbations of asthma and COPD are poorly understood^10,11^.

Comparatively little is known about the variation within the human immune system in relation to the patterns of response to viruses and bacteria at a population level. Modelling of the cytokine responses of cells, such as peripheral blood mononuclear cells (PBMCs), to stimulation with viral and bacterial stimuli, may be informative in this regard^12^, but a comprehensive systems approach is required to advance our understanding of these complex immune responses. We have previously used machine learning to describe six patterns (immunophenotypes) of multiple cytokine responses to rhinovirus by human PBMCs, each associated with different risk profiles for hospitalizations with RTIs, early-life wheezing, childhood asthma, and allergic sensitization^13^. A recent study which investigated bacteria- and fungi-induced profiles of seven cytokines in PBMCs using unsupervised hierarchical clustering found that cytokine responses were predominantly organized around a response to pathogens, rather than specific immune pathways or cytokines^14^. Although variability in the heritability of cytokine levels has been reported^12,14,15^, some cytokine responses (including IL-6) are heritable, with several cytokine Quantitative Trait Loci (cQTL) identified^14^.

Herein we describe the inter-individual variation and the patterns of responses of 28 cytokines (including type I, II & III interferons (IFNs), immune and pro-inflammatory cytokines and chemokines, listed in Supplementary Table S1) to 15 stimuli (listed in Table 1), including 5 common respiratory pathogens (2 bacteria and 3 viruses), 9 toll-like receptor (TLR) ligands and phytohemagglutinin (PHA), to stimulate T cells in PBMCs in 307 children from a population-based birth cohort. We used an integrated systems approach, applying machine learning methods to data, to uncover enormous differences between children in the architecture of cytokine responses to infectious agents/stimuli. To investigate mechanisms potentially explaining such variable responses between children, we performed cQTL analysis on IL-6 responses to bacterial stimulation because IL-6 was strongly induced by the eight bacterial stimuli. In addition, we examined cQTL associations with important asthma-related traits in these children.

**Table 1:** Infectious agents, Toll-like receptor (TLR) ligands and other stimuli used to stimulate peripheral blood mononuclear cells (PBMCs).

Stimulus and abbreviation	Concentration	Group	Source	Pattern recognition receptors used	Mean % data missing^‡^
Stimulation Media (RPMI1640 with l-glutamine, 2% HEPES, 1% sodium bicarbonate, 1% penicillin/streptomycin and 10% foetal bovine serum)	NA	NA	Invitrogen	NA	0.7
Rhinovirus serotype 16 (RV16)	MOI of 1	Viral	Grown in our laboratory, from ATCC	TLR3/7/8/9, RIG-I, MDA5	0.8
Respiratory syncytial virus (RSV)	MOI of 1	Viral	Grown in our laboratory, from ATCC	TLR3/7/8/9, RIG-I, MDA5	1.3
*Haemophilus influenzae* ATCC strain 49247 (Hin)	10^6^ CFU/mL	Bacterial	Grown in our laboratory, from ATCC	TLR4, NOD1	2.2
*Streptococcus pneumoniae* strain D39 (Strpn)	10^6^ CFU/mL	Bacterial	Grown in our laboratory	TLR1/2/6, TLR4, dectin-1	2.9
Phytohemagglutinin (PHA)	10 µg/mL	T cell	Roche diagnostics	NA	5.1
Rhinovirus serotype 1B (RV1B)	MOI of 1	Viral	Grown in our laboratory, from ATCC	TLR3/7/8/9, RIG-I, MDA5	5.8
Polyinosinic: polycytidylic acid (polyIC)	100 µg/mL	Viral	Sigma-Aldrich	TLR3	NA
Lipopolysaccharide (LPS)	10 µg/mL	Bacterial	Sigma-Aldrich	TLR4	13.4
Resiquimod (R848)	1 µM	Viral	Source Bioscience	TLR7/8	17.8
Class A CPG oligonucleotide (CpG-A)	1 µM	Viral	Invivogen	TLR9	32.7
Synthetic triacylated lipoprotein PAM3CSK4 (PAM)	10 µg/mL	Bacterial	Invivogen	TLR1/2	27.8
Peptidoglycan (PGN)	10 µg/mL	Bacterial	Invivogen	TLR2	33.1
Fibroblast stimulating ligand-1/synthetic diacylated lipoprotein (FSL)	1 µg/mL	Bacterial	Invivogen	TLR2/6	37.3
Flagellin (Fla)	100 ng/mL	Bacterial	Invivogen	TLR5	44.5
Lipoteichoic acid from *Staphylococcus aureus* (LTA)	25 µg/mL	Bacterial	Invivogen	TLR2	48.8

*MOI* multiplicity of infection, *CFU* colony-forming unit, *TLR* toll-like receptor, *RIG-I* retinoic acid-inducible gene I, *MDA5* melanoma differentiation-associated protein 5, *NOD1* Nucleotide-binding oligomerization domain-containing protein 1, *NA* not applicable.

^‡^If insufficient cells were available for culture with every stimulus, the stimuli were used in rank order of this table, thus the mean % missing data increases down the table (last column).

## Results

### Study population and descriptive data

The children studied were participants in the Manchester Asthma and Allergy Study (MAAS), a population-based birth cohort (see “Methods” section for further details). Of the 921 children who were followed up at age 11 years, 340 provided PBMCs for the current studies (Supplementary Fig. S1); there were no differences in demographic data, environmental exposures, asthma/allergy and lung function between children providing PBMCs compared to those who did not (Supplementary Table S2). Following quality control (see “Methods” section), cytokine data was available for analysis from 307 children (157 males), of whom 273 had genetic data. A complete cytokine-stimulus data set (all 28 cytokines for all 15 stimuli) was available for 130 children; the mean percentage of missing values in the cytokine-stimulus pairs was 18.3% (inter-quartile range 2.6–32.9%; for individual stimuli see Table 1).

**Figure 1:**
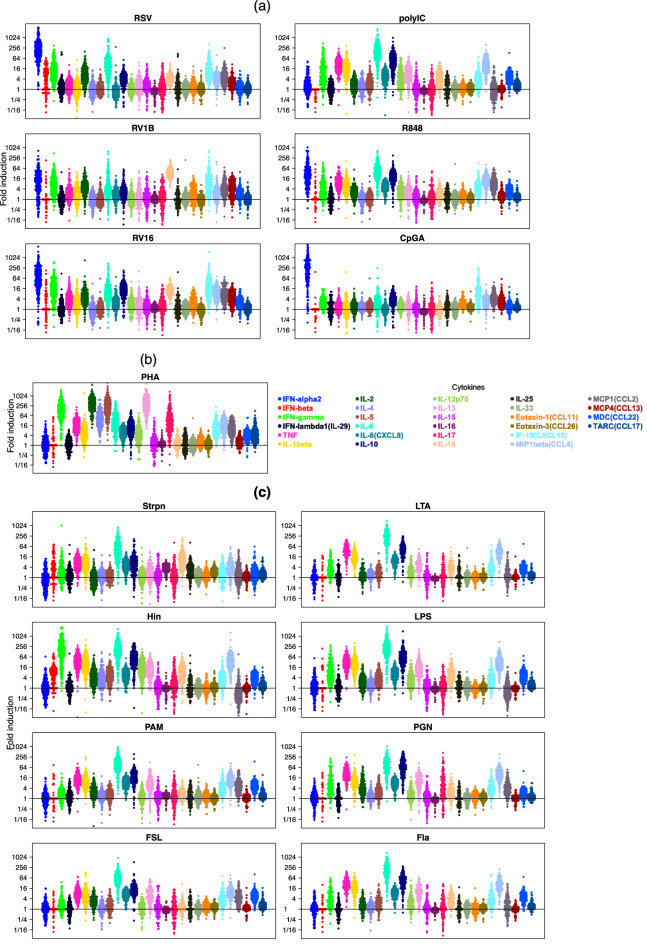
Cytokine response patterns according to stimulus. Fold induction (response/media) of a panel of 28 innate, inflammatory and immune cytokines from PBMCs; each dot represents a child. (**a**) Viral stimuli: RSV, RV1B, RV16, polyIC, R848 and CpGA; (**b**) T-cell stimulus: PHA. (**c**) Bacterial stimuli: Strpn, LTA, Hin, LPS, PAM, PGN, FSL and Fla. This figure was generated using beeswarm package in R (version 3.6.3, https://www.R-project.org/).

Cytokine responses (fold induction: response/media) are presented in Fig. 1, with similar stimuli grouped together: (a) viral stimuli: respiratory syncytial virus (RSV), rhinovirus 1B (RV1B), rhinovirus 16 (RV16), polyinosinic:polycytidylic acid (polyIC), resiquimod (R848) and class A CpG oligonucleotide (CpGA); (b) the T-cell stimulus: phytohemagglutinin (PHA); (c) bacterial stimuli: *Streptococcus pneumoniae* (Strpn), lipoteichoic acid (LTA), *Haemophilus influenzae* (Hin), lipopolysaccharide (LPS), synthetic triacylated lipoprotein PAM3CSK4 (PAM), peptidoglycan (PGN), fibroblast stimulating ligand-1 (FSL) and flagellin (Fla). Significantly induced cytokine/stimulus pairs are shown as mean fold-inductions relative to media (Supplementary Fig. S2), and as absolute concentrations in pg/mL (Supplementary Fig. S3).

### Substantial variation in cytokine induction between stimuli

The viral stimuli (RSV, RV1B, RV16 and R848) had very similar patterns of induction, with very strong induction (~ 1000-fold or greater) of interferon (IFN)-α2 and IL-6, strong induction (~ 256-fold) of IFN-γ and CXCL10/IP-10, and many other cytokines with moderate induction of ~ 64-fold or less (Fig. 1a, Supplementary Figs. S2, S3). In contrast, the eight bacterial stimuli were very strong inducers of IL-6, strong inducers of IL-10 and moderate to strong inducers of TNF, IL-1β and CCL4/MIP1β, the Gram-negative stimuli Hin and LPS were very strong/strong inducers of IFN-γ, while the Gram-positive stimuli Strpn and PAM and FSL were only weak inducers of IFN-γ (Fig. 1c, Figs. S2, S3). PHA strongly induced the T cell related cytokines IFN-γ, IL-2, IL-5, IL-13 and IL-17, moderately induced IL-4 and IL-6 and induced many other cytokines/chemokines less strongly (Fig. 1b, Supplementary Figs. S2, S3).

### Substantial variation in cytokine induction between individual participants

For most cytokine/stimulus pairs where induction was strong, responses between children differed vastly in magnitude (Fig. 1); e.g. for IFN-α2 induction by RSV, RV16 and CpGA, some children had no induction, while others were induced > 1000-fold (Fig. 1a). The scale of variation between children for induction of IFN-γ and IL-6 to the live bacteria Strpn and Hin was similarly large (Fig. 1c).

### Architecture of cytokine/stimulus responses

**Figure 2:**
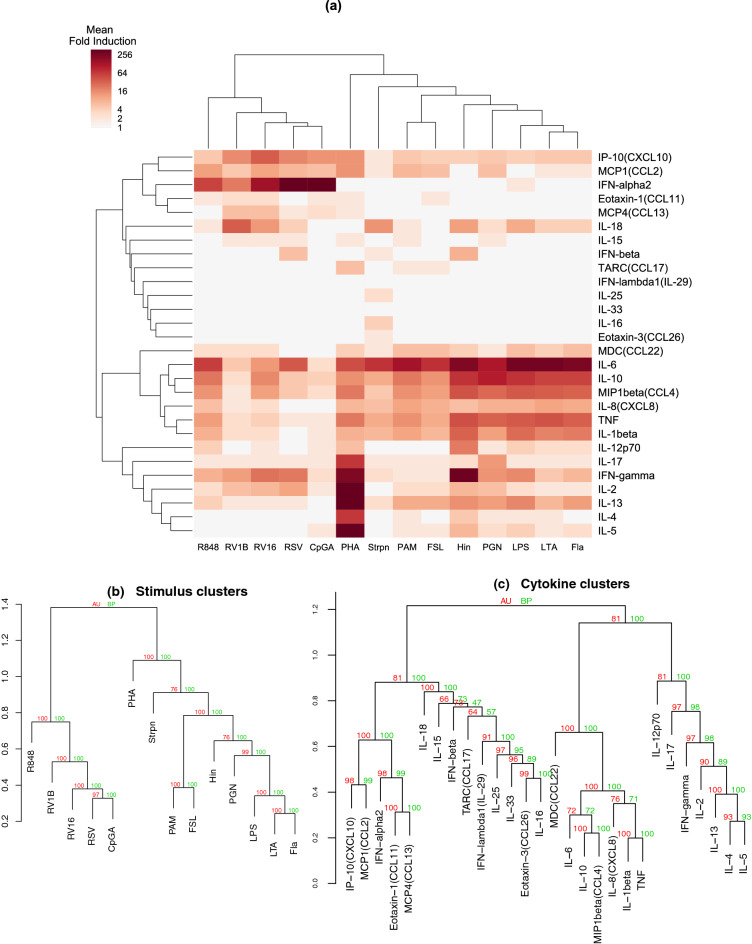
Hierarchical clustering (HC) of cytokine responses. (**a**) Heatmap of the mean level of response for each cytokine-stimulus pair (307 children, media-normalised). (**b**) Hierarchical tree for stimuli. (**c**) Hierarchical tree for cytokines. Values on nodes indicate probability values (%) of the found clusters appearing in bootstrap resampling. Green numbers are estimates from ordinary bootstrap probabilities (BP); red numbers are “approximately unbiased” (AU) estimates from multi-scale bootstrap resampling, which are less biased estimates than BP (see “Methods” section). Plot (**a**) was generated using seaborn package in Python (version 3.8, https://www.python.org/). Plots (**b,c**) were generated using pvclust package in R (version 3.6.3, https://www.R-project.org/).

Hierarchical clustering was used to cluster 28 cytokines and 15 stimuli respectively. Results of clustering using media-normalised data (log-transformed fold induction) from all 307 children (including those with imputed data, Fig. 2) and 130 children with a complete data set (Supplementary Fig. S4) were very similar, indicating that our handling of missing data was robust (see “Methods” section). PolyIC unexpectedly clustered separately from viral stimuli, and next to LPS (Supplementary Fig. S4); polyIC (from the same source, Sigma-Aldrich) was confirmed to be heavily contaminated with endotoxin; see Supplementary Text 2. Excluding polyIC from the clustering did not materially change the results for the other stimuli (Fig. 2). Thus, for all remaining analyses we used media-normalised data from 307 children, with polyIC excluded.

Following clustering, the average response for each stimulus/cytokine pair was plotted as a heat map (Fig. 2a). The hierarchical trees of stimuli across all cytokine responses and cytokines across all stimuli are expanded in Fig. 2b,c respectively, with high bootstrap values indicating stability of the tree structures. Except for PHA which was placed close to the root of the tree on its own, the stimuli clustered into two distinct groups; all viruses/viral ligands (R848, RV1B, RV16, RSV, CpGA) clustered together, and all bacteria/bacterial ligands clustered together (Fig. 2b). Sub-level distinction revealed further features, with Strpn forming a singleton sub-cluster on its own, and PAM and FSL grouping together, distinct from a sub-cluster containing Hin, PGN, LPS, LTA and Fla.

The cytokine clustering identified four cytokine groups (Fig. 2c). The first cluster included IFN-α2 and IFN-induced chemokines IP-10/CXCL10, MCP1/CCL2, eotaxin-1/CCL11 and MCP4/CCL13. The second cluster was a mixed group of 9 cytokines that were mostly induced to low levels, or not induced. The third cluster included CCL22/MDC, the pro-inflammatory cytokines (IL-1β, IL-6, CXCL8/IL-8, CCL4/MIP1β and TNF), and IL-10. The final cluster comprised IL-12p70 and a group of T cell-derived cytokines (IL-17, IFN-γ, IL-2, IL-4, IL-5 and IL-13) that were strongly induced by PHA.

To visualise cytokine and stimulus patterns, we applied principal component analysis (PCA) on the *Child-Cytokine* × *Stimulus* matrix and *Child-Stimulus* × *Cytokine* matrix*,* respectively (Supplementary Fig. S5, Methods and Supplementary Text 1).

### Substructure of cytokines, stimuli, and cytokine/stimulus pairs

**Figure 3:**
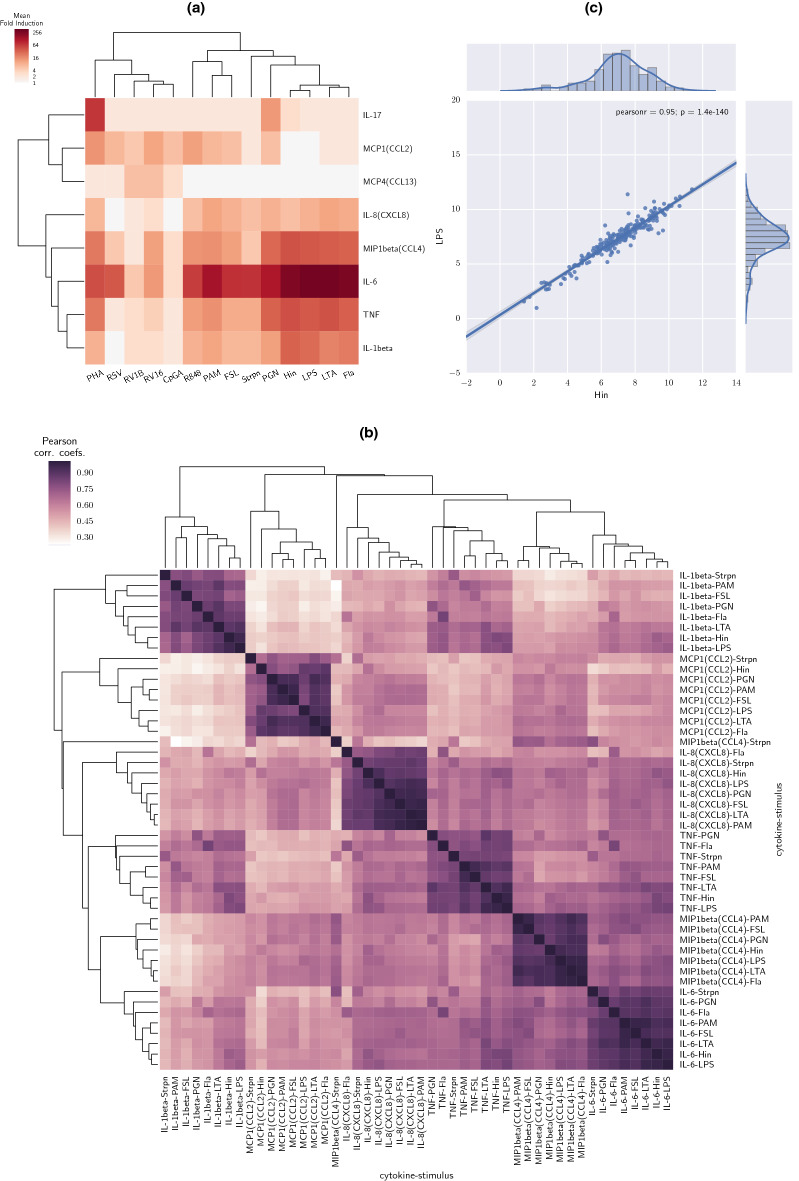
Hierarchical clustering for pro-inflammatory cytokine data. (**a**) HC with heatmap on pro-inflammatory cytokine responses to all stimuli. (**b**) Pairwise Pearson correlation coefficients of production of pro-inflammatory cytokines in response to bacterial ligands. MCP4 and IL-17 were weakly induced and so are excluded from this panel. (**c**) Scatter plot with IL-6 responses to LPS versus IL-6 responses to Hin, and Pearson correlation coefficient between LPS and Hin. This figure was generated using seaborn package in Python (version 3.8, https://www.python.org/).

In order to investigate how cytokines in different functional groups (Supplementary Tables S1, S3) respond to various types of stimuli, we applied clustering and PCA on the subsets of data including only pro-inflammatory (Fig. 3, Supplementary Fig. S6) cytokines and anti-viral cytokines (Supplementary Fig. S7), respectively.

For pro-inflammatory cytokines, two subgroups were found (Fig. 3a). The larger consisted of cytokines that were strongly induced by all the bacterial stimuli, as well as by PHA and R848, with the strongest being IL-6 (up to mean 280-fold induction) and CCL4/MIP1β, TNF, IL-1β and CXCL8/IL-8 (6–40-fold induction) (Figs. S2, S3). The other subgroup included IL-17, CCL2/MCP1 and CCL13/MCP4, among which IL-17 was induced very strongly by PHA, moderately by PGN and weakly by other stimuli. CCL13/MCP4 was moderately induced only by viral stimuli (and most strongly by RVs) and CCL2/MCP1 was induced by most (but more strongly by viral) stimuli.

For stimuli, one cluster contained all the bacterial stimuli, and the viral stimulus R848 (Fig. 3a). A subgroup was formed by R848, PAM and FSL, based upon their induction of CCL2/MCP1. PGN was unique in strongly inducing IL-17. Within the second cluster (viral stimuli/PHA), CpGA was a very weak inducer of pro-inflammatory cytokines. RVs induced most pro-inflammatory cytokines weakly/moderately, and RSV strongly induced IL-6 (38-fold, Supplementary Fig. S2), weakly/moderately induced TNF and CCL4/MIP1β (two and fivefold respectively, Supplementary Fig. S2), but failed to induce IL-1β and CXCL8/IL-8. PHA moderately/strongly induced pro-inflammatory cytokines, CCL2/MCP1, and was unique in strongly inducing IL-17 (67-fold, Supplementary Fig. S2). The PCA was consistent with the clustering (Supplementary Fig. S6). In keeping with this clustering, we found that within individuals, there was a strong correlation between the levels of each pro-inflammatory cytokine in response to all of the bacterial ligands tested (Fig. 3b); e.g. IL-6 response to LPS predicted IL-6 response to Hin (Fig. 3c, coefficient = 0.95, *P* = 1.4E−140).

Similarly, clustering and PCA with the anti-viral cytokine responses revealed a clear division between viral and bacterial stimuli (Supplementary Fig. S7a,b). Responses to four viral stimuli (R848, RV16, RSV and CpGA) were strongly correlated, whereas responses to RV1B were less correlated to them (Supplementary Fig. S7b). A strong correlation within the levels of CXCL10/IP-10 and IFN-α2 in response to all viral ligands was also found (Supplementary Fig. S7c). See Supplementary Text 3 for more details on the anti-viral cytokine responses substructure.

### cQTLs of IL-6 induction by bacterial stimuli

**Figure 4:**
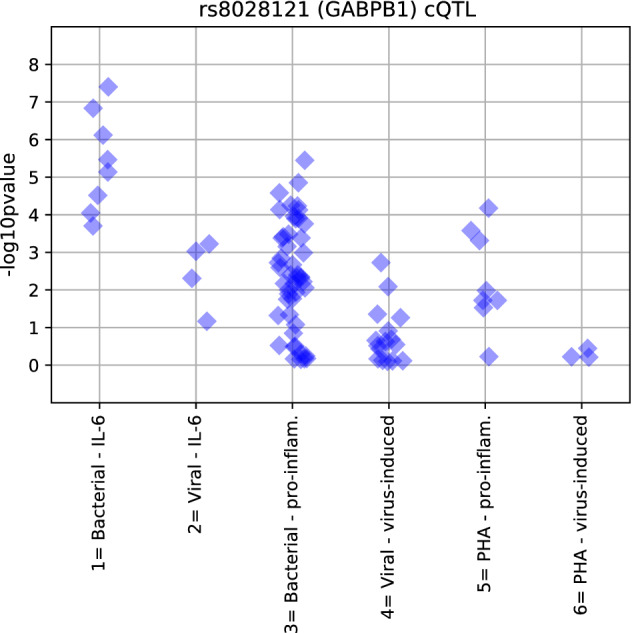
Association of rs8028121 with cytokine-stimulus pairs. The cQTL rs8028121 was associated with IL-6 production in response to bacterial stimuli (x-axis = 1) more strongly than IL-6 production in response to viral stimuli (x-axis = 2), other pro-inflammatory cytokine responses to bacterial stimuli (x-axis = 3), virus-induced cytokine responses to viral stimuli (x-axis = 4), pro-inflammatory cytokine responses to PHA (x-axis = 5), and virus-induced cytokine responses to PHA (x-axis = 6).

As IL-6 was strongly induced by eight bacterial stimuli and showed a wide range of between-subject responses, we assessed if any Single Nucleotide Polymorphisms (SNPs) were cytokine Quantitative Trait Loci (cQTL) of IL-6 responses to bacterial stimuli. We identified ten significant independent cQTLs in nine loci (*P* < 5 × 10^–8^); one locus was associated with Hin, two with LPS, three with Fla, two with LTA and one with both Fla and LTA (Table 2, Supplementary Fig. S8). These nine loci were also associated with IL-6 levels in response to the other bacterial stimuli at nominal significance in 61 of 62 tests (Supplementary Table S4), but less frequently associated with IL-6 responses to viral stimuli, with other pro-inflammatory cytokine responses to the remaining bacterial stimuli, or with PHA responses (Supplementary Fig. S9, Supplementary Table S4). The cQTL most consistently associated with IL-6 responses was rs8028121 (Fig. 4), which lies upstream of and is a known significant eQTL for *GABPB1* (Table 2). *GABPB1 is* a subunit of the transcription factor nuclear respiratory factor 2 (NRF2) which regulates expression of antioxidant proteins that protect against oxidative damage triggered by infections^16^. We replicated previously reported associations between the SNPs *rs351250* and *rs683458*1 and IL-6 responses to pathogens^12^. We report associations between *rs6834581* and IL-6 levels in response to bacterial (Hin, LPS, LTA) and viral (RSV, R848) stimuli as well as *rs351250* and IL-6 levels in response to the bacterial stimuli Fla and Hin (Supplementary Table S5) at nominal significance levels.

**Table 2:** SNPs associated with IL-6 induction in response to bacterial stimuli.

SNPs	Chrom	Gene	Allele*	Gene region	Stimulus	*P*-value	Beta	SE
rs73624755^c^	20	*ABHD16B*	G	Upstream	Fla	9.05E−14	− 4.00	0.54
rs73624755^c^	20	*ABHD16B*	G	Upstream	LTA	4.50E−09	− 2.48	0.42
chr7: 67342638:D	7	–	A	Intergenic	LPS	4.84E−09	− 1.64	0.28
rs117007889	7	*KIAA1324L*	A	Intron	LTA	5.56E−09	− 3.11	0.53
rs77609006^d^	19	*MUC16*	A	Intron	LTA	1.04E−08	− 2.42	0.42
rs7440580	4	–	C	Intergenic	Hin	1.32E−08	− 1.26	0.22
rs139089467	6	*CYP2AC1P*	G	Pseudogene	Fla	1.59E−08	− 1.50	0.27
chr2: 221028028:I	2	*AC114765.1*	CTATA	Intron	Fla	2.05E−08	− 1.88	0.33
rs111481643^a^	6	*LRRC16A*	G	Intron	LPS	3.40E−08	− 1.36	0.25
rs8028121^b^	15	*GABPB1*; *USP8*	C	Upstream	Fla	3.95E−08	− 1.56	0.28

*Alleles associated with lower IL-6 release. *SE* standard error. No loci were significantly associated with IL-6 responses to Strpn, PGN, PAM and FSL at genome-wide significance.

^a^rs111481643 is a significant eQLT for LRRC16A in 21 cohorts (eqtlgen).

^b^rs8028121 is a significant eQLT for GABPB1-AS in 15 cohorts (eqtlgen).

^c^rs73624755 is an eQTL for ABHD16B in CD4 T-cells at nominal significance (eQTL catalogue).

^d^rs77609006 eQTL catalogue for MUC16 in naïve monocytes at nominal significance (eQTL catalogue).

### Clinical phenotypes are associated with IL-6 cQTL

We next tested if these nine cQTLs were associated with asthma-related traits. Children carrying the minor allele of *rs1390894*67 had lower IL-6 levels in response to the bacterial stimulus Fla (Supplementary Fig. S8) and were more likely to have had an unscheduled visit to a primary care physician for asthma in the first 8 years of life (20.6% vs 15.8%, *P* = 0.03, Table 3). Children carrying the minor allele of *chr2: 221028028:I* had lower IL-6 levels in response to the bacterial stimulus Fla (Supplementary Fig. S8) and were more likely to require oral corticosteroids for acute asthma attacks after age 3 years (24.4% vs 15.5%, *P* = 0.03, Table 3), and were more likely to have more severe disease (exemplified by requiring a higher Global Initiative for Asthma treatment step) by age 8 years compared to those with the common allele (27.8% vs 15.6%, *P* = 0.01, Table 3).

**Table 3:** Clinical phenotypes associated with IL-6 cQTLs.

rs number	Chr	Gene ID	Minor allele	IL6 levels in minor allele carriers	Phenotype	Minor allele carrier % Case	Minor allele carrier % Control	*P* value
rs139089467	6	*CYP2AC1P*	G	Lower	Unscheduled visit for asthma	20.6	15.8	0.03
chr2: 221028028:I	2	*AC114765.1*	CTATA	Lower	Oral steroids use	24.4	15.5	0.03
chr2: 22128028:I	2	*AC114765.1*	CTATA	Lower	GINA* step ≥ 2 at age 8	27.8	15.6	0.01

*GINA Global Initiative for Asthma; step 1 treatment is used for mild intermittent asthma. Step 2 treatment or above is required to treat more severe asthma.

## Discussion

To facilitate better understanding of human immune responses to pathogens, we present a comprehensive description of the architecture of multiple cytokine responses by human PBMCs to the most common viruses and bacteria which cause respiratory disease, plus ligands for each human TLR, and the T-cell stimulus PHA. We describe distinct and discrete clustering of the microbes/microbial ligands (into viral, bacterial and PHA), and also of cytokines (into pro-inflammatory, T-cell derived, IFNs/IFN-induced, and not/weakly induced), which was consistently seen both in PCA and hierarchical clustering. Additional features of cytokine responses to individual stimuli were revealed. The observed patterns were biologically plausible and resilient to handling of missing data and normalisation strategies. Moreover, we found that in individual children, specific cytokine responses were correlated within each functional and stimulus group. We observed an enormous range of cytokine inductions between different children, with some children having no or almost no induction, while others had inductions of > 1000-fold, for example IL-6 induction by bacterial stimuli. Finally, as an exemplar of the strong heritable component of immune responses, we identified several novel cQTL for IL-6 induction by bacterial stimuli and observed that genetic associates of lower IL-6 production were linked to markers of more severe asthma in this population.

The hierarchical clustering identified three distinct stimulus groups—all viruses/viral ligands clustered together, as did the bacteria/bacterial ligands, with PHA alone in the third group. Indeed, the clustering so clearly separated bacteria from viruses that when we identified polyIC clustered with LPS, we suspected contamination of the polyIC with endotoxin, which we subsequently confirmed and therefore excluded polyIC from further analysis. A considerable proportion of the variance within the dataset was explained by the separation of viral from bacterial ligands, and the first 2 components of the *Child-Cytokine* PCA explained 76% of the variance. Our results indicate that despite the huge differences in the *size* of responses seen between individual children, the *patterns* of cytokine response to viral and bacterial stimuli were broadly similar across children. However, cytokines in the same functional groupings could behave differently: in the antiviral group, IFN-α2 and CXCL10/IP-10, which were strongly induced by viruses, edged away from the weakly or not induced IFN-β, IL-15 and IL-29/IFN-λ1; CXCL10/IP-10 and IFN-γ edged away from the rest of Th1 cytokines; in the pro-inflammatory group, IL-6 was distant from IL-17; IL-13 was parted from the other Th2/proTh2 cytokines; and the two “regulatory” cytokines IL-10 and IL-18 responded differently.

We observed that bacterial stimuli (with R848) induced IL-6, CCL4/MIP1β, TNF, IL-1β and CXCL8/IL-8. Pro-inflammatory cytokines in this subgroup were generally less strongly induced by the viral stimuli, whereas the other subgroup of pro-inflammatory cytokines consisted of CCL2/MCP1 and CCL13/MCP4 which tended to be more strongly induced by the viral stimuli and IL-17 which was moderately and strongly induced only by PGN and PHA respectively.

However, individual organisms exhibited some unique features, which is unsurprising given their distinct biology. For example, within the bacterial cluster, Strpn was separated as a much weaker inducer of IFN-γ and pro-inflammatory cytokines than Hin, despite being stimulated with identical numbers of CFUs and inducing other cytokines (IL-16, eotaxin-3/CCL26) more strongly than Hin. Within the viral cluster, RV16 was a stronger inducer of IFN-α, IFN-γ and pro-inflammatory cytokines than RV1B; in contrast, RV1B was a stronger inducer of IL-18. These differences between RV strains that are very closely related in terms of genetic sequence^17^, may be a result of them using a different receptor to enter cells (RV16 using ICAM-1^18^ and RV1B using the LDL receptor^19^) or perhaps subtle differences in their RNA being recognised by pattern recognition receptors thereby inducing differences in intracellular signalling.

As we analysed samples from children in an unselected birth cohort, we had limited sample volumes and were unable to determine cell type composition of PBMCs prior to performing cell culture, and therefore could not adjust analyses for differences in cell-type proportions. However others have recently shown only a minor effects of cell count differences on cytokine production^20^, and we therefore do not believe that, with the possible exception of IFN-λ, this has adversely affected our findings.

Although we measured 28 cytokines using sensitive multiplex assays with limits of detection that were on average 10–20 fold lower than ELISAs used in previous studies^12,14^, we identified a cluster of 9 cytokines that were not induced or were only weakly induced. Contributing factors may include the single time of sampling of supernatant 24 h and the fact that the PBMCs had been stored in liquid nitrogen. In contrast to our results where IFN-λ was not induced by any stimulus, freshly collected PBMCs do produce IFN-λ when stimulated by RV16^21^, thus it is also possible that the cell type capable of producing IFN-λ (likely dendritic cells) was depleted by the freeze/thaw process, or storage in liquid nitrogen. The lack of induction of IFN-β, while IFN-α2 was strongly induced is likely also a consequence of storage and of timing of sampling occurring only at 24 h, as IFN-α is induced about twofold more than IFN-β at 24 h in RV16-stimulated fresh PBMCs^21^.

Our analyses revealed the cytokines to have a more complex structure than the stimuli. Nineteen cytokines that were induced by multiple stimuli clustered into three distinct cytokine groups, reflecting their functional characteristics. One cluster included IFN-α2 and the IFN-induced chemokines CXCL10/IP10, CCL2/MCP1, CCL13/MCP4 and CCL11/eotaxin-1, which were induced predominantly by the viral stimuli. A second cluster included the pro-inflammatory cytokines (IL-1β, IL-6, CXCL8/IL-8, CCL4/MIP1β and TNF) and IL-10, which were induced strongly by bacterial stimuli. IL-10 is generally considered anti-inflammatory/regulatory, rather than pro-inflammatory, but it was nevertheless induced strongly by the bacterial stimuli, suggesting induction of both a pro-inflammatory and an anti-inflammatory response by these stimuli. A final cluster comprising a group of T cell-derived cytokines, with IL-12p70 as a more distantly related member of this group, were strongly induced by PHA. Within this group IFN-γ was very strongly induced by Hin, but was also induced by all other stimuli. IL-2, IL-17 and the Th2 cytokines were induced weakly to moderately by most. CCL22/MDC was alone as a side branch of the pro-inflammatory/IL-10 cluster and distant from CCL17/TARC, suggesting that their induction may be regulated differently^22^, even though they both bind the same CCR4 receptor, and both are associated with Th2 responses. Analyses on stratified data from functional subgroups of cytokines indicated subtle changes in stimuli structures, and the heat maps revealed that the cytokines had a stronger and more consistent response in the appropriate group of stimuli.

A striking observation was that where cytokines were strongly induced by a stimulus, we frequently observed an enormous range of inductions between children, with some children having no or almost no induction, while others had inductions of > 1000-fold. Notable examples include induction of IFN-α2 in response to live viruses (RSV and RV16) and CpGA, and induction of IFN-γ and IL-6 in response to Hin, Strpn and all bacterial ligands. In a recent study, Bakker et al*.*^23^ assessed multiple genetic and non-genetic host factors that influence cytokine production and showed that genetics is a major contributor to the inter-individual variations in cytokine production after pathogen stimulation. As IL-6 was the most strongly induced cytokine in response to bacterial stimuli, we investigated if any SNPs were cQTLs of these responses. Previous studies have shown that signalling responses of immune cells when cultured with IL-6 are not heritable^15^, but that the baseline IL-6 levels in serum and IL-6 responses to bacterial stimuli are^14,23^. Li et al*.* reported an association between *Coxiella burnetii* induced IL-6 levels and *rs351250* as well as polyIC-induced IL-6 levels and rs6834581 (in linkage disequilibrium with the missense SNP rs4833095)^12^. We successfully replicated the association between these SNPs and IL-6 responses to bacterial and viral stimuli. In addition, we identified nine loci associated with IL-6 responses to bacterial stimuli. Our results indicate that these loci may contribute to IL-6 responses to multiple stimuli and are not necessarily specific to a single microbial ligand, suggesting potential roles in multiple infective/inflammatory pathways leading to IL-6 production. Finally, for several of these loci we found associations with relevant clinical features, namely asthma exacerbations, which are precipitated by both viral and bacterial infections in childhood^24,25^ and disease severity, linking lower IL-6 responses with unfavourable outcomes in relation to asthma attacks and asthma severity in childhood.

In summary, we describe the architecture of immune responses, using a data-driven approach to model multiple cytokine responses to the most common respiratory viruses and bacteria which cause respiratory disease, to ligands of all human TLRs, and to PHA. We observed that despite the large differences in the size of responses seen, the patterns of cytokine response to viral and bacterial stimuli were broadly similar between children. The cytokine responses assembled into discrete and biologically plausible groupings, and the bacterial and viral ligands were clearly distinguished from each other. Further exploration of the genetic mechanisms underlying the different results observed with bacterial and viral stimulation is the focus of our future work. Our analysis identified genetic associates of immune responses, although the mechanisms underlying these remains to be elucidated. Our findings demonstrate that the data-driven approach described herein, which reveals robust immunologically and microbiologically plausible clustering, provides a framework for understanding the relationships between host responses to infection and their clinical consequences.

## Methods

### Study design

The Manchester Asthma and Allergy Study (MAAS) is a population-based birth cohort^26^. Participants were recruited from the maternity catchment area of Wythenshawe and Stepping Hill Hospitals (50 square miles of South Manchester and Cheshire), a stable mixed urban–rural population (http://www.maas.org.uk). The study was approved by South Manchester Health Authority Medical Research Ethics Committee for the age 0–5 years follow-up (ERP/94/032, approval received on 24/03/1994), and subsequently by South Manchester Local Research Ethics Committee for the age 5–8 years follow-up (SOU/00/258 and SOU/00/259, approval received on 20/12/2000) and for the age 8–11 years follow-up (03/SM/400, approval received on 05/12/2003). The study is registered at ISRCTN registry (http://www.isrctn.com/ISRCTN72673620). Written informed consent was obtained from parents. All methods were performed in accordance with the relevant guidelines and regulations.

### Screening and recruitment

Subjects were recruited prenatally and followed prospectively. All pregnant women were screened for eligibility at antenatal visits (8th–10th week of pregnancy) between 1995 and 1997. Of the 1499 couples who met the inclusion criteria (< 10 weeks of pregnancy, maternal age > 18 years), 288 declined to take part and 27 were lost to follow-up between recruitment and birth of a child. A total of 1184 participants had some evaluable data.

#### Follow up

Children have been followed prospectively, and attended review clinics at ages 1, 3, 5, 8 and 11 years. We carried out home visits for study participants who could not attend clinic appointments. At age 11 years, a total 921 subjects followed up, among which 340 subjects provided PBMCs, and 307 subjects (157 males, 150 females) were included in this study after QC (Supplementary Fig. S1). We extracted questionnaire data and data from General Practitioner (GP)-held medical records including prescriptions, acute wheeze episodes, oral steroid prescriptions and hospital admissions for asthma or wheeze. Timing, type of visit, symptoms, indication and prescriptions for each encounter were noted. Presence of wheeze and asthma diagnosis was recorded. Prescription information included timing, drug name, dose and indication.

### Definition of clinical outcomes

A trained physician reviewed the written and computerized primary care medical records for each child. Every piece of information was entered into that child’s designated database space. All consultations with health care providers including hospital admissions, hospital outpatient visits and use of the out of hours services, with linked prescriptions (drug name, route of administration and the dose) was separately entered by the date of the event, allowing the calculation of child’s age in days at each event. Information captured included location, the type of visit, reason for that particular consultation and any relevant symptoms (such as cough, wheeze, shortness of breath, fever) or diagnoses (such as asthma or bronchiolitis).

#### Unscheduled asthma/wheeze visits

This was defined as any unscheduled visit for asthma or wheeze excluding asthma/wheeze related hospital admissions.

#### Oral steroid receipt

All oral steroid prescriptions issued for lower respiratory indication or oral steroid prescriptions not linked to the visits, but issued to children who were known to have asthma or wheeze were extracted for the analysis.

#### Asthma medication and GINA step

All asthma medication prescribing (either linked to the specific consultation, or repeat medications not linked to the specific visit) were extracted. Drug name, dose and classification were noted. Asthma medication was defined as prescription for any drug belonging to one of these generic classes: 1. Inhaled short acting beta 2 agonist (SABA); 2. Inhaled ipratropium bromide; 3. Inhaled cromoglicate; 4. Inhaled long acting beta 2 agonist (LABA); 5. Inhaled corticosteroid; 6. Combination inhaler (Inhaled LABA + Inhaled corticosteroid); 7. Leukotriene receptor antagonist.

Asthma medication receipt for each year of follow up was defined according to the age of child at each issue of asthma medication. These data were used to classify the children according to the GINA Stepwise approach (www.ginasthma.org, as issued in 2010, so as to be contemporaneous to the prescriptions issued): STEP 1: As-needed SABA with no controller; STEP 2: Regular low dose ICS or leukotriene modifier plus as-needed SABA; STEP 3: Low-dose ICS plus LABA or Medium-or high-dose ICS or Low-dose ICS plus leukotriene modifier or Low-dose ICS plus sustained release theophylline; STEP 4: Medium-or high-dose ICS plus LABA (or leukotriene modifier); STEP 5: Oral glucocorticosteroid or Anti IgE treatment.

#### Admission for asthma/wheeze after the third-year life

This is derived from the age of child at each asthma/wheeze admission, defined as dichotomous yes–no outcome, depending on whether child was admitted for asthma/wheeze after the age of three.

### In vitro cell studies

We chose 15 infectious agents and stimuli: 3 common viruses (respiratory syncytial virus [RSV], rhinovirus 1B [RV1B], rhinovirus 16 [RV16]), 2 bacteria (*Streptococcus pneumoniae* [Strpn] and *Haemophilus influenzae* [Hin]), 9 ligands for all human TLRs (polyinosinic:polycytidylic acid [polyIC], resiquimod [R848], class A CpG oligonucleotide [CpGA], lipoteichoic acid [LTA], lipopolysaccharide [LPS], synthetic triacylated lipoprotein PAM3CSK4 [PAM], peptidoglycan [PGN], fibroblast stimulating ligand-1 [FSL] and flagellin [Fla]) and phytohemagglutinin (PHA) to stimulate T cells, to stimulate PBMCs to investigate the viral and bacterial responses (Table 1).

### PBMC collection and stimulation experiments

Blood for in vitro cell studies was collected at clinical follow-up at age 11 years, and PBMCs were extracted and cryopreserved in liquid nitrogen^20^. Cryobanked PBMCs were shipped in two batches for cell stimulations^20,27^. The first batch (n = 54) was analysed ~ 3 years before the second batch (n = 286), in order to obtain preliminary data to demonstrate that our approach was feasible and to obtain funding for the larger analysis. Cells were thawed and counted to assess viability. Cells (2 × 10^5^ per well) were re-suspended in media (RPMI)+/− one of the chosen stimuli up to a volume of 200 µL/well and cultured for 24 h; for live viruses, after 1 h the virus was replaced with fresh media. Supernatants were harvested 24 h post-stimulation and stored at − 80 °C. We used a combination of knowledge from existing literature as well as preliminary studies to select a dose that would induce relevant cytokines at the 24 h time point we were obliged to restrict ourselves to, due to numbers of cells available. If insufficient cells were available for culture with each stimulus individually, the stimuli were used in rank order of Table 1, hence the mean % missing data increases down the Table 1 (last column).

### Cytokine measurements

Protein levels of 28 cytokines (including cytokines, chemokines and interferons, but for ease of reference referred to henceforth simply as “cytokines”, Supplementary Table S1) were measured using the Meso Scale Discovery^®^ multiplex kits (http://www.mesoscale.com). Samples below the lower detection limit of the assay (defined per cytokine per batch, Supplementary Table S1) were assigned a value of ½ the lower detection limit.

### Quality control and data preprocessing

All samples with cell viability < 5% upon thawing were excluded from further analysis (n = 13). In addition, samples meeting all the following criteria were excluded as inadequate responders: viability < 20% and IL-2 response to PHA < 5 pg/mL and IFN-α response to RSV < 5 pg/mL and IFN-γ response to RV16 < 7 pg/mL and IL-6 response to Hin < 5 pg/mL (n = 19). One child was excluded with 32% viability, as no cytokine response was seen to any stimulus.

Cryobanked PBMCs were analysed in two batches for cell stimulations. Linear regression analysis was used to assess whether viability was predicted by batch assignment and whether viability was associated with cytokine levels. We found no significant difference in viability across batches (P = 0.90) and no significant association between cytokine production levels and viability, except for IL-16 responses to Strpn (Pearson’s r = 0.24, P < 0.001) and IL-18 responses to Strpn (Pearson’s r = 0.25, P < 0.001). Children with < 25% viability have similarly distributed cytokine expression levels compared to those with > 25% viability. Therefore, we did not include viability in subsequent analyses.

We found a significant difference in cytokine responses across batches using both raw values and log-transformed response values relative to media. We then investigated whether these differences were because of differences in disease characteristics across batches. We found significant differences between batches within different disease phenotypes (i.e. there were significant batch differences between participants with and without asthma, or atopy, etc.). We therefore decided to adjust for batch in all subsequent analyses and clustering. We used regression analysis to assess a coefficient evaluating the difference in mean stimuli responses for each cytokine that is due to difference between batches. These coefficients were defined using batch 2 (n = 286) as the baseline, which had more samples and tended to have higher values than batch 1 (n = 54). This coefficient was subtracted from the individual responses of batch 1. In the case when adjusted raw values were below the detection limit of the batch 2 assay, they were assigned a value of ½ the detection limit of the assay. For later statistical analysis, the batch-adjusted response values were media-normalised when needed.

The missing cytokine response values were imputed by inferring them from probabilistic principal component analysis^28^ (PPCA) using the Expectation Maximisation (EM) algorithm. Bayesian model selection^29^ was used to determine the optimal dimensionality of the data for PPCA. This approach was compared with the simple mean imputation, where we simulated missing data by randomly removing a certain percentage of the known values (using the real data missing rate) and computed the imputation errors for both methods. The proposed method yielded better imputation accuracy than the mean imputation in all our simulations (Supplementary Fig. S10).

### Hierarchical clustering and principal component analysis

Pre-processed data were analysed using hierarchical clustering (HC) and principal component analysis (PCA). The cytokine-stimulus response pairs available for each child were projected into two new variable spaces, creating multiple cytokine/stimulus vectors per child. In other words, we created one data matrix where columns represented cytokine types and rows represented children, where the elements in each row were filled in with cytokine responses under a certain stimulus, i.e., *Child-Stimulus* × *Cytokine* matrix. Similarly, a data matrix of the same type was created by using stimuli as column indices, i.e. *Child-Cytokine* × *Stimuli* matrix, with each row representing one child and cytokine combination. PCA was executed on both data matrices to project the high dimensional data (28-dimensional in *Child-Stimulus* × *Cytokine* matrix and 15-dimensional in *Child-Cytokine* × *Stimulus* matrix for media-subtracted data) onto lower-dimensional spaces to visualise the data. Hierarchical clustering was executed on both *Child-Stimulus* × *Cytokine* and *Child-Cytokine* × *Stimulus* data matrices using the average linkage method and correlation distance to cluster cytokines and stimuli, respectively. Linkage methods and distance metrics were chosen after testing the stability of results under different linkage/distance functions. Clustering robustness was evaluated by using bootstrap resampling techniques and calculating the probability values for each cluster, i.e. the frequencies of the found clusters appearing in bootstrap resampling. We used R package pvclust^30^ which implements two types of probability values: bootstrap probability (BP) value is calculated by the ordinary bootstrap resampling; approximately unbiased (AU) probability value is calculated by using a multi-scale bootstrap resampling method^31^. An asymptotic theory proves that AU value is less biased than BP value^31^. In our clustering, bootstrap analysis was performed with the number of bootstrap replications being 1000. An AU value > 95% indicates that the cluster is highly supported by the data^30^.

Within smaller functional groups (bacterial stimuli with pro-inflammatory cytokines and viral stimuli with antiviral cytokines), we further analysed the correction between cytokine-stimulus pairs and performed hierarchical clustering to the *Child* × *Cytokine-Stimulus* matrix, i.e. each child represented by a reduced set of cytokine-stimulus pairs. We visualised this clustering with Pearson’s correlation from the bivariate analysis carried out on cytokine-stimulus response pairs.

The results from the media-normalised data are presented in the main manuscript, where the log2-transformed level of the media control was subtracted from the log2-transformed stimulus response to reflect the response relative to media. In addition, we performed analyses without this normalisation and with the media as an additional independent control to permit comparison of each stimulus with media control (Supplementary Figs. S11, S12).

### Genotyping, quality control and imputation

DNA was genotyped and imputed as previously described^32^. Briefly: DNA samples from 880 Caucasian children were genotyped on an Illumina 610 quad chip. The Illumina genotypes were called using the Illumina GenCall application following the manufacturer’s instructions. Non-Caucasian samples were excluded to ensure there was no population stratification by reviewing a PCA plot of the genotypes (Supplementary Fig. S13). Quality control criteria for samples included: 97% call rate, exclusion of samples with an outlier autosomal heterozygosity (scree-plot visualisation) gender validation and sequenome genotype concordance. Quality control criteria for SNPs included a 95% call rate, Hardy–Weinberg equilibrium (HWE) > 5.9 × 10^–7^, minor allele frequency > 0.005.

Genotypes were subsequently prephased (SHAPEIT v2.r644) and imputed (IMPUTE V2.2.2). We used the “1000 Genomes Phase I integrated variant set” reference genotypes to impute 560 K genotypes to yield 37,985,335 genotypes per sample.

### Cytokine quantitative trait loci (cQTLs)

We used an additive model (SNPTEST V2.5.1^33,34^; score method) to identify genome wide significant (*P* < 5 × 10^–8^) cQTLs for IL-6 responses to 8 bacterial stimuli (Table 1): Hin, Strpn, LPS, PAM, PGN, FSL, Fla, LTA. We retained all genotyped SNPs and removed imputed SNPs with info scores of < 0.75 and minor allele frequencies (MAF) < 0.05. We used the media-normalised cytokine response data with no missing data imputation for cQTL analysis rather than raw cytokine responses. Normality of the distribution was tested using Kolmogorov–Smirnov test for goodness of fit against normal distributions. The normality test showed that more IL-6 to bacterial stimulus responses followed normal distribution after data transformation (see Supplementary Fig. S14).

We assessed if cQTLs that were significantly associated with IL-6 induction to bacterial stimuli (at genome wide significance, *P* < 5 × 10^–8^) were specific to IL-6 responses to bacterial ligands or were seen in other stimulus/cytokine pairs. To do this we tested for associations (at nominal significance, *P* < 0.05) between those nine SNPs and IL-6 responses to other Bacterial stimuli (Table 1) (n = 7); IL-6 responses to Viral stimuli (Table 1) (n = 4); the response of pro-inflammatory cytokines to Bacterial stimuli (Supplementary Table S1) (n = 56); the response of virus-induced cytokines to Viral stimuli (Supplementary Table S1) (n = 16); the response of pro-inflammatory cytokines to PHA (n = 7) and the response of virus-induced cytokines to PHA (n = 4).

We also tested for associations between 7 SNPs, selected from all available SNPs in our genotyped and imputed dataset, that were found to be previously associated with IL-6 production^12^ and IL-6 response to bacterial stimuli in our dataset.

We assessed if our cQTLs were known significant eQTLs by querying the eQTLGene Consortium (https://www.eqtlgen.org/cis-eqtls.html; accessed 28 April 2022). In addition, we queried the eQTL catalogue (https://www.ebi.ac.uk/eqtl/; accessed 28 April 2022) to assess if our cQTLs are eQTLs in studies that utilised the following cell types: T cells, monocytes, neutrophils, NK cells, CD4+ T cells, CD8+ T cells, Th17 cells, Th1 cells, Th2 cells, Treg naive, Treg memory, CD16+ monocytes, Cultured fibroblasts, EBV-transformed lymphocytes. We defined nominal significance as P < 0.05.

### Association between clinical phenotypes and IL-6 cQTLs

We used an additive model (SNPTEST V2.5.1^33,34^; score method) to identify associations between our cQTLs and (1) Unscheduled asthma/wheeze visits, (2), Oral steroid receipt and Asthma Medication and (3), GINA where GINA Cases were defined as Step 2 or greater and GINA Controls received only Step 1 treatment for asthma.

### Statistical analysis

Descriptive statistics were given for the absolute levels of cytokines (batch adjusted, in pg/mL) for each cytokine stimulus pair (Supplementary Fig. S3). For statistical significance of up-regulated stimulus induction in relation to media control, a right one-sided t-test was used with the null hypothesis that the mean of the fold inductions in log scale is greater than zero at a significance level α adjusted by the total number of cases to test, i.e., the number of cytokine stimulus pair. We set α to be 0.05/(28 × 15) ≈ 0.000119. Significance test results and mean fold induction for each cytokine stimulus pair are summarised in Supplementary Fig. S3.

The R package “pvclust” was used for hierarchical clustering. Probabilistic principal component analysis for missing data imputation was carried out using the MATLAB function “ppca”. The R package “beeswarm” was used to generate plots of cytokine response patterns. PCA plots and heatmaps are based on the “seaborn” Python package.

## Supplementary Information

Supplementary Information 1.

Supplementary Information 2.

Supplementary Figure S3.

Supplementary Figure S8.

Supplementary Figure S9.

Supplementary Table S4.

Supplementary Table S5.

## Data Availability

The cytokine data presented in this study are provided in the Supplementary Information files. Summary statistics related to the cQTL analysis are available at the NHGRI-EBI GWAS catalog (GCP ID: GCP000279, https://www.ebi.ac.uk/gwas). We do not have consent to share the genotyping data and GP records from the participants; however, any external researcher can request data access for any specific projects from the MRC/BBSRC Flagship Consortium in Systems Immunology of the Human Life Course. The consortium embraces open access principles for sharing published data with other researchers. Existing access mechanisms are leveraged to enable managed access, thereby respecting the expectations of privacy and confidentiality of the research participants who contributed the data and/or samples. Requests for data will normally be agreed to, if these do not compromise governance restrictions, publications, confidentiality, or commercially exploitable results.

## Code availability

Python and R code to reproduce the cytokine analysis is available at https://github.com/ManchesterBioinference/Systems-Immunology-Cytokine-Responses.
