# Supplementary material for: A systems immunology approach to investigate cytokine responses to viruses and bacteria and their association with disease
Source: Sci Rep. 2022 Aug 5;12:13463. doi: 10.1038/s41598-022-16509-4 (PMC9356009; doi:10.1038/s41598-022-16509-4)
Supplement: Supplementary file 1 — Supplementary Information 1. [file 41598_2022_16509_MOESM1_ESM.docx]

Supplementary Materials for

**A systems immunology approach to investigate cytokine responses to viruses and bacteria and their association with disease**

Lijing Lin^1†^, John A. Curtin^1†^, Eteri Regis^2^, Aurica Hirsman^2^, Rebecca Howard^1^, Mauro Tutino^1^, Michael R. Edwards^2^, Mattia Prosperi^3^, Angela Simpson^1^*‡, Magnus Rattray^1^‡, Adnan Custovic^2^‡, Sebastian L. Johnston^2^‡

*Corresponding author. Email: angela.simpson@manchester.ac.uk

**This PDF file includes:**

Supplementary Text 1, Text 2, Text 3

Supplementary Figs. S1, S2, S4 to S7, S10 to S14

Supplementary Tables S1 to S3

**Other Supplementary Materials for this manuscript include the following:**

Figs. S3, S8, S9 (in separate PDF files)

Tables S4, S5 (in separate Excel files)

Supplementary Text

1. Cytokine/stimulus patterns with principal component analysis (PCA***)***

By performing PCA for the *Child-Cytokine x Stimulus* matrix (see Materials and Methods), 5 components explained 90% of variance. Supplementary Fig. S5a shows the projection of *Child-Cytokine x Stimulus* data matrix into the first three dimensions of the PCA space (in two 2-D planes). Each point represents one child-cytokine pair and the lines denote the contribution of each stimulus to each component. Stimuli variables on the PC1 *vs*. PC2 plot (Supplementary Fig. S5a, left) pointed to two main directions, one consisting of all bacterial stimuli, plus PHA, and the other with all the viral stimuli. The separation of PHA and Strpn from the other stimuli can be seen from the PC3 *vs*. PC2 plot (Supplementary Fig. S5a, right), which is consistent with the hierarchical clustering results (Fig. 2b).

PCA on the *Child-Stimulus x Cytokine* data matrix yielded 16 components explaining 90% of variance. Cytokine grouping results were consistent with the HC where cytokine features point to four clearly different directions best seen in the PC2/PC3 plot (Supplementary Fig. S5b right, the weakly induced cytokine group being just visible in the centre of the figure).

To visualise the cytokine data more clearly, we presented the data in separate plots with cytokines grouped by functional roles (Supplementary Table S1 and S3) for PC1/PC2 (Supplementary Fig. S5c). Data points for each cytokine generally clustered together. The positions of cytokines in each functional group largely overlapped with each other (consistent with their functional activities), except for IFN-α2, IL-6 and IL-10 which edged away from other cytokines in their group (Supplementary Fig. S5c).

2. polyIC contamination

Because polyIC unexpectedly clustered separately from viral stimuli, and next to LPS (Supplementary Fig. S4), we suspected the polyIC was contaminated with endotoxin. For the cell stimulations reported herein we used polyIC from Sigma-Aldrich, catalogue number P0913 10mg, lot number not recorded. After performing the clustering and suspecting contamination none of this lot remained to enable us to measure endotoxin levels and determine whether contamination was present. We therefore obtained two fresh lots of polyIC (Sigma-Aldrich, catalogue number P0913, 10mg, lot number #094M4146V and 50mg, lot number #065M4064V) and tested these for contamination with endotoxin using the limulus ameobocyte lysate QSL-1000 assay (Lonza) performed according to the manufacturer’s instructions. We found that #094M4146V contained 2.68 and #065M4064V 2.87 EU/mL of endotoxin, very similar to the endotoxin concentration in the LPS stimulus used (2.73 EU/mL), thereby confirming that the polyIC used for the cell stimulations was likely also heavily contaminated with endotoxin, and therefore not a specific viral mimic/ligand for TLR-3, as intended. Consequently, all polyIC data were removed from further analysis. All other stimuli tested had very low or absent endotoxin concentrations.

3. Substructure of anti-viral cytokine/stimulus patterns with hierarchical clustering and PCA

For anti-viral cytokines, stimulus clustering revealed a clear division between viral and bacterial stimuli (Supplementary Fig. S7a). IFN-α2 was only induced by viral stimuli – ranging from 21-fold for RV1B to 511-fold for CpGA (Supplementary Fig. S2). In contrast CXCL10/IP-10 was induced by all stimuli (2-fold for Strpn to 35-fold for RV16), while IFN-β was only induced by Strpn, Hin and RSV. IL-29/IFN-λ1 was not induced by any stimulus, and levels of IL-15 were generally low.

PCA (Supplementary Fig. S7b) revealed larger between-child variation within both CXCL10/IP-10 and IFN-α2 compared to IFN-β, IL-29/IFN-λ1 and IL-15. Anti-viral cytokine responses to four viral stimuli (R848, RV16, RSV and CpGA) were strongly correlated, whereas the responses to RV1B were less correlated to these (Supplementary Fig. S7b, right panel), although we note that PC2 explained only a small proportion of the variance in the data (6.9%). We found a strong correlation within the levels of CXCL10/IP-10 and IFN-α2 in response to all viral ligands (Supplementary Fig. S7c).

**Fig. S1. Study cohort enrolment, follow-up, and exclusions.**

**Fig. S2. Statistically significant up-regulated cytokine-stimulus pairs** **(fold change in relation to media control, one-sided t-tests)**. Geometric mean fold-inductions are indicated numerically within in each box, and colored according to grey scale. White blocks indicate non-significant pairs (where the stimulus did not induce that cytokine relative to media).

**Fig. S4. Hierarchical clustering of cytokine responses for** **130 children with complete dataset**, **polyIC INCLUDED. (a)** Heatmap of the mean level of response for each cytokine-stimulus pair (media-normalised).

**Fig. S4. Hierarchical clustering of cytokine responses for** **130 children with complete dataset**, **polyIC INCLUDED. (b)** Hierarchical tree for stimuli**. (c)** Hierarchical tree for cytokines. Values on nodes indicate probability values (%) of the found clusters appearing in bootstrap resampling. Green numbers are estimates from ordinary bootstrap probabilities (BP); red numbers are “approximately unbiased” (AU) estimates from multi-scale bootstrap resampling, which are less biased estimates than BP (see Materials and Methods). Plot (a) was generated using seaborn package in Python (version 3.8, <https://www.python.org/>). Plots (b) and (c) were generated using pvclust package in R (version 3.6.3, <https://www.R-project.org/>).

**Fig. S5.** **Principal component analysis (PCA) with all cytokines and stimuli. (a)** Projection of *Child-Cytokine x Stimulus* data matrix onto the first three principal components: PC1 vs. PC2 (left); PC3 vs. PC2 (right). **(b).** Projection of *Child-Stimulus x Cytokine* data matrix onto the first three principal components: PC1 vs. PC2 (left); PC3 vs. PC2 (right). Each point represents one child-stimulus pair, coloured by the corresponding stimulus kind. Straight lines represented directions and relative weights of cytokine variables on each PC.

**Fig. S5.** **Principal component analysis (PCA) with all cytokines and stimuli.** (c) Replotting (a) with cytokines separated into smaller functional groups for clarity (PC1 vs. PC2 only). This figure was generated in Python (version 3.8, <https://www.python.org/>).

**Fig. S6. PCA for pro-inflammatory cytokine responses.** Projection of *Child-Cytokine x Stimulus* data including only pro-inflammatory cytokines onto the first two principal components: PC1 vs. PC2, for cytokine responses to all stimuli (left panel) and bacterial stimuli (right panel), respectively. Each point represented one child-cytokine pair, coloured by the corresponding cytokine. Straight lines represented directions and relative weights of stimulus variables on each PC. This figure was generated in Python (version 3.8, <https://www.python.org/>).

**Fig. S7.** **Hierarchical clustering and PCA for anti-viral cytokine data. (a)** Hierarchical clustering with heatmap on anti-viral cytokine responses to all stimuli**. (b)** PCA on *Child-Cytokine x Stimulus* sub-matrices using only anti-viral cytokines responses to all stimuli (left) or viral stimuli (right), respectively.

**Fig. S7.** **Hierarchical clustering and PCA for anti-viral cytokine data. (c)** Pairwise Pearson correlation coefficients of production of anti-viral cytokine in response to virus/viral ligands. IFN-β, IL-29/IFN-λ1 and IL-15 were weakly induced and so are excluded from this panel. This figure was generated in Python (version 3.8, <https://www.python.org/>).

**Fig. S10. Accuracy comparison of two missing data imputation methods: probabilistic PCA (PPCA) vs. mean imputation.** The assessment was carried out by randomly removing a proportion of data from the original dataset, imputing them using different methods, and then comparing the imputed data against the observed values. PPCA was applied with reduced dimensions chosen from 1 to 14. For each choice of reduced dimension, we ran 10 tests each with 12% of the observed data randomly removed. The average imputation error ${err=\sum(R}_{obs}-R_{imp})$ over the 10 tests was reported (y-axis) for each reduced dimension (x-axis) for both PPCA (blue) and mean imputation (red).

**Fig. S11.** **Hierarchical clustering of cytokine response (including media as an independent variable) for 307 children. (a)** Heatmap of the mean raw responses for each cytokine-stimulus pair**.**

**Fig. S11.** **Hierarchical clustering of cytokine response (including media as an independent variable) for 307 children. (b)** Hierarchical tree for stimuli. **(c)** Hierarchical tree for cytokines. Values on nodes indicate probability values (%) of the found clusters appearing in bootstrap resampling. Green numbers are estimates from ordinary bootstrap probabilities (BP); red numbers are “approximately unbiased” (AU) estimates from multi-scale bootstrap resampling, which are less biased estimates than BP (see Materials and Methods). Plot (a) was generated using seaborn package in Python (version 3.8, <https://www.python.org/>). Plots (b) and (c) were generated using pvclust package in R (version 3.6.3, <https://www.R-project.org/>).

**Fig. S12.** **PCA with all cytokines and stimuli on log-transformed data (media as an independent variable and not used for normalisation).** **(a)** Projection of *Child-Cytokine x Stimulus* data matrix onto the first three principal components: PC1 vs. PC2 (left); PC3 vs. PC2 (right). Each point represented one child-cytokine pair, coloured by the corresponding cytokine. Straight lines represented directions and relative weights of stimulus variables on each PC. **(b)**. Projection of *Child-Stimulus x Cytokine* data matrix onto the first three principal components: PC1 vs. PC2 (left); PC3 vs. PC2 (right). Each point represented one child-stimulus pair, coloured by the corresponding stimulus kind. Straight lines represented directions and relative weights of cytokine variables on each PC. This figure was generated in Python (version 3.8, <https://www.python.org/>).

**Fig. S13.** **PCA of MAAS genotypes for population structure.** We compared MAAS genotypes with genotypes from HapMap3 reference populations. European descent (CEU), Yoruba (YRI), Hans Chinese (CHB) and Japanese (JPT). This figure was generated using ggplot2 package in R (version 3.6.3, https://www.R-project.org/).

(a)

(b)

**Fig. S14.** **Distributions of IL-6 responses to 8 bacteria stimuli: Hin, Strpn, LPS, PAM, PGN, FSL, Fla, LTA.** Normality of the distribution was tested using Kolmogorov-Smirnov test for goodness of fit against normal distributions; p-value was shown on each plot with p < 0.05 indicating non-normality. (a) Distributions of raw cytokine levels. (b) Distributions of media-normalised responses: all values were log2 transformed and with log2 media response subtracted, i.e. log2 transformed fold induction.

**Table S1. Cytokines (including cytokines, chemokines and interferons, but for ease of reference referred to henceforth simply as “cytokines”) measured in supernatant.** Light grey font indicates cytokines that were not induced or very weakly induced. The last two columns are lower detection limits in pg/mL (defined per cytokine per batch). Samples below detection limits in the table below were assigned a value of ½ the detection limit of the assay.

| **Functional groups (ascribed from our knowledge of published literature)** | **Cytokine names** | **Detection limits (pg/mL)** | |
| --- | --- | --- | --- |
|  |  | **Batch 2 (n=286)** | **Batch 1 (n=54)** |
| **Pro-inflammatory** | TNF | 3 | 0.5 |
|  | IL-1β | 1 | 0.2 |
|  | IL-6 | 2 | 0.5 |
|  | IL-8(CXCL8) | 2 | 0.5 |
|  | IL-17 | 1.52 | 1.52 |
|  | MIP1β (CCL4) | 0.94 | 0.5 |
|  | MCP1(CCL2) | 0.83 | 0.5 |
|  | MCP4 (CCL13) | 8 | 30 |
| **Anti-Viral** | IFN-α2 | 0.2 | 0.2 |
|  | IFN-β | 7.44 | 7.44 |
|  | IFN-λ1(IL-29) | 20 | 20 |
|  | IP-10(CXCL10) | 30 | 40 |
|  | IL-15 | 0.335 | 0.335 |
| **Th2/proTh2** | IL-4 | 0.1 | 0.05 |
|  | IL-5 | 0.3 | 0.1 |
|  | IL-13 | 0.5 | 0.1 |
|  | IL-25 | 1.88 | 1.88 |
|  | IL-33 | 0.33 | 0.33 |
|  | Eotaxin-1(CCL11) | 10 | 10 |
|  | Eotaxin-3(CCL26) | 80 | 40 |
|  | MDC(CCL22) | 103 | 40 |
|  | TARC(CCL17) | 10 | 10 |
| **Th1** | IFN-γ | 2 | 0.5 |
|  | IL-12p70 | 0.4 | 0.06 |
|  | IL-15 | 0.335 | 0.335 |
|  | IL-18 | 0.3 | 0.3 |
|  | IP-10(CXCL10) | 30 | 40 |
| **T-Cell** | IL-2 | 0.2 | 0.1 |
|  | IL-16 | 4.25 | 4.25 |
|  | IL-17 | 1.52 | 1.52 |
| **Regulatory** | IL-10 | 0.5 | 0.1 |
|  | IL-18 | 0.3 | 0.3 |

**Table S2. Characteristics of children providing or not providing PBMCs for cytokine data at age 11 years.**

SE: standard error.

†Current asthma: Any two of the following three features: (1) Current wheeze; (2) Asthma medication in the last 12 months; (3) Physician-diagnosed asthma ever.

††% Predicted FEV1: Percentage of predicted air volume expelled in the first second of a forced expiration.

|  | **Children Providing PBMCs for Cytokine Data at Follow-up (n=340)**  **N (%), or mean (SE)** | **Children Not Providing PBMCs for Cytokine Data at Follow-up (n=581)**  **N (%), or mean (SE)** | **P-value** |
| --- | --- | --- | --- |
| **Gender (Male)** | 175/340 (51.5) | 323/581 (55.6) | 0.24 |
| **Mean age at follow up** | 11.49 (0.03) | 11.51 (0.02) | 0.64 |
| **Older siblings** | 188/338 (55.6) | 302/576 (52.4) | 0.37 |
| **Maternal smoking (pregnancy)** | 34/335 (10.1) | 60/563 (10.7) | 0.91 |
| **Maternal asthma at birth** | 57/340 (16.8) | 126/581 (21.7) | 0.07 |
| **Paternal asthma at birth** | 48/339 (14.2) | 81/581 (13.9) | 0.92 |
| **Maternal atopy at birth** | 189/334 (56.6) | 343/566 (60.6) | 0.26 |
| **Paternal atopy at birth** | 204/333 (61.3) | 365/564 (64.7) | 0.32 |
| **Current asthma**† | 62/340 (18.2) | 102/577 (17.7) | 0.86 |
| **% Predicted FEV1**†† | 94.83 (0.40) | 94.42 (0.35) | 0.45 |

^†^

**Table S3. Strength of evidence supporting the selection and grouping of specific cytokines as pro-inflammatory and anti-viral.**

| **Cytokine** | **Group** | **Strength of evidence†** | **References** |
| --- | --- | --- | --- |
| CCL13/MCP4 | Pro-inflammatory | Sequence data showing promoters all contain binding sites for pro-inflammatory transcription factors AP-1/AP-2, NF-κ B, c/EBP-β | [*1*] |
| TNF | Pro-inflammatory | In any cell type, the role of NF-κB, AP-1/AP-2 or c/EBP-β  in inducing each gene with any given stimuli as shown by siRNA knockdown, over-expression studies, specific pharmacological inhibitors or site-directed mutagenesis of each site within the promoter. | [*2*] |
| IL-1β | Pro-inflammatory |  |  |
| CCL4/MIP1β | Pro-inflammatory | Sequence data showing promoters all contain binding sites for pro-inflammatory transcription factors AP-1/AP-2, NF-κB, c/EBP-β  In any cell type, the role of NF-κB, AP-1/AP-2 or c/EBP-β in inducing each gene with any given stimuli as shown by siRNA knockdown, over-expression studies, specific pharmacological inhibitors or site-directed mutagenesis of each site within the promoter. | [*3, 4*] |
| CCL2/MCP1 | Pro-inflammatory |  | [2*, 5-7*] |
| IL-6 | Pro-inflammatory | As above and:  In any leukocyte, a defined role for TLR2/4/6 specific ligands or bacteria in inducing NF-κB, AP-1/AP-2 or c/EBP-β in the expression of each gene with any specific stimuli as shown by siRNA knockdown, over-expression studies, pharmacological inhibitors or site-directed mutagenesis of each site within the promoter. | [*8-10*] |
| CXCL8/IL-8 | Pro-inflammatory |  | [*2, 11, 12*] |
| IL-17 | Pro-inflammatory | Sequence data showing promoters all contain binding sites for pro-inflammatory transcription factors AP-1 and NF- κ B | [*13*] |
| IFN-α2, | Antiviral | The cytokines IFN-α2, IFN-λ1/IL-29, IL-15 and CXCL10/IP-10 were considered related to IFN action and anti-viral immunity as they either have properties similar to type I IFN-beta or are directly induced by the actions of interferons or viruses and have a role in the anti-viral response | [*14-18*] |
| IFN-λ1(IL-29) | Antiviral |  |  |
| CXCL10/IP-10 | Antiviral |  |  |
| IL-15 | Antiviral |  |  |

**†According to the references cite**

**Other Supplementary Materials**

**Fig. S3.** **(Separate PDF File) Boxplots for absolute levels of 28 cytokines in pg/mL for medium control and each of the 15 stimuli shown as individual panels.**

Each panel presents one cytokine; when cytokine responses were highly variable among different stimuli, a right y-axis was needed with the corresponding stimuli using right y-axis indicated in pink. Each plot shows lower quartile (Q1, 25th percentile), median (red line), and upper quartile (Q3, 75th percentile). Interquartile range IQR = Q3-Q1 and values outside the range Q1-1.5 * IQR and Q3 +1.5 *IQR (where grey whiskers extended) were deemed outliers and omitted from the plot (except in IFN-beta where outliers are shown for the cases where IQR = 0). One-sided t-test was used to test if the cytokine was significantly induced by the stimulus: h=0 no; h=1 yes; p: p-value of the test; CI: lower bound in the 95% confidence interval; fold mean: robust mean (excluding outliers) of the corresponding fold induction.

**Fig. S8.** **(Separate PDF File) Locuszoom Plots and IL-6 levels by genotypes for cQTL.**

A locuszoom plot for each cQTL as well as IL-6 levels by genotype is provided for rs139089467 (IL-6 levels in response to Fla), rs117007889 (IL-6 levels in response to LTA), rs111481643 (IL-6 levels in response to LPS), rs77609006 (IL-6 levels in response to LTA), rs73624755 (IL-6 levels in response to LTA), rs73624755 (IL-6 levels in response to Fla). rs8028121 (IL-6 levels in response to Fla), rs7440580 (IL-6 levels in response to Hin). IL-6 levels by genotype for chr7:67342638:D and chr2:221028028:I (associated with IL-6 levels in response to FLS and Fla respectively) are also provided but it was not possible to generate locuszoom plots for these two SNPs.

**Fig. S9.** **(Separate PDF File) Cytokine QTL of bacterial stimuli-induced IL-6 are associated with bacterial stimuli-induction of other pro-inflammatory cytokines.**

Nine SNPs underlying ten cQTL were associated with IL-6 response to at least one bacterial stimulus (x-axis=1). We assessed if these cQTL were also associated with IL-6 levels in response to viral stimuli (x-axis=2), other pro-inflammatory cytokine responses to bacterial stimuli (x-axis=3), virus induced cytokine responses to viral stimuli (x-axis=4), PHA induced pro-inflammatory cytokine responses (x-axis=5), PHA induced virus-induced cytokine responses (x-axis=6). This figure was generated in Python (version 3.8, <https://www.python.org/>).

**Table S4. (Separate Excel file) Association of cytokine-stimulus pairs with ten cQTLs.**

**Table S5. (Separate Excel file) Replication of published associations**.

We replicated associations between rs351250 and IL-6 responses to the bacterial stimulus (*Coxiella burnetii*) and between rs6834581 and IL-6 responses to the viral stimulus (polyIC) (*12*); in MAAS we replicated associations between rs6834581 and IL-6 response to bacterial (Hin, LPS, LTA) and viral (RSV, R848) stimuli as well as rs351250 and IL-6 responses to Fla and Hin.

**References**

1. H. Hein *et al.*, Genomic organization, sequence, and transcriptional regulation of the human eotaxin gene. *Biochemical and biophysical research communications* **237**, 537-542 (1997).

2. J.-H. Kim *et al.*, Decursin inhibits induction of inflammatory mediators by blocking NF-κB activation in macrophages. *Molecular pharmacology* **69(6)**, 1783-1790 (2006).

3. Z. Zhang *et al.*, CCAAT/enhancer-binding protein β and NF-κB mediate high level expression of chemokine genes CCL3 and CCL4 by human chondrocytes in response to IL-1β. *Journal of Biological Chemistry* **285**, 33092-33103 (2010).

4. P.-C. Tseng *et al.*, Helioxanthin inhibits interleukin-1β-induced MIP-1β production by reduction of c-jun expression and binding of the c-jun/CREB1 complex to the AP-1/CRE site of the MIP-1β promoter in Huh7 cells. *Biochemical pharmacology* **76**, 1121-1133 (2008).

5. Y.-C. Lin *et al.*, Tumor necrosis factor-alpha inhibitors suppress CCL2 chemokine in monocytes via epigenetic modification. *Molecular immunology* **83**, 82-91 (2017).

6. M. L. L. Richard, T. K. Nowling, D. Brandon, D. K. Watson, X. K. Zhang, Fli-1 controls transcription from the MCP-1 gene promoter, which may provide a novel mechanism for chemokine and cytokine activation. *Molecular immunology* **63**, 566-573 (2015).

7. C. Bethel-Brown, H. Yao, G. Hu, S. Buch, Platelet-derived growth factor (PDGF)-BB-mediated induction of monocyte chemoattractant protein 1 in human astrocytes: implications for HIV-associated neuroinflammation. *Journal of neuroinflammation* **9**, 262 (2012).

8. V. Gambhir *et al.*, The TLR2 agonists lipoteichoic acid and Pam3CSK4 induce greater pro-inflammatory responses than inactivated Mycobacterium butyricum. *Cellular immunology* **280**, 101-107 (2012).

9. S. Sarkar *et al.*, Suppression of the NF-κB pathway by diesel exhaust particles impairs human antimycobacterial immunity. *The Journal of Immunology*, 1101380 (2012).

10. S. Seshadri, Y. Kannan, S. Mitra, J. Parker-Barnes, M. D. Wewers, MAIL regulates human monocyte IL-6 production. *The Journal of Immunology* **183(8)**, 5358-5368 (2009).

11. A. Uehara *et al.*, Muramyldipeptide and diaminopimelic acid‐containing desmuramylpeptides in combination with chemically synthesized Toll‐like receptor agonists synergistically induced production of interleukin‐8 in a NOD2‐and NOD1‐dependent manner, respectively, in human monocytic cells in culture. *Cellular microbiology* **7**, 53-61 (2005).

12. J. Im *et al.*, Induction of IL-8 expression by bacterial flagellin is mediated through lipid raft formation and intracellular TLR5 activation in A549 cells. *Molecular immunology* **47**, 614-622 (2009).

13. X. K. Liu, X. Lin, S. L. Gaffen, Crucial role for nuclear factor of activated T cells (NFAT) in T cell receptor-mediated regulation of human interleukin-17. *Journal of Biological Chemistry* **279(50)**, 52762-52771 (2004).

14. N. W. Bartlett *et al.*, Defining critical roles for NF‐κB p65 and type I interferon in innate immunity to rhinovirus. *EMBO molecular medicine* **4**, 1244-1260 (2012).

15. P. A. Wark *et al.*, IFN-γ–induced protein 10 is a novel biomarker of rhinovirus-induced asthma exacerbations. *Journal of Allergy and Clinical Immunology* **120**, 586-593 (2007).

16. V. Laza-Stanca *et al.*, The role of IL-15 deficiency in the pathogenesis of virus-induced asthma exacerbations. *PLoS Pathog* **7**, e1002114 (2011).

17. K. Onoguchi *et al.*, Virus infections activate type I and type III interferon genes through a common mechanism. *Journal of Biological Chemistry* **282(10)**, 7576-7581 (2007).

18. A. Meager, K. Visvalingam, P. Dilger, D. Bryan, M. Wadhwa, Biological activity of interleukins-28 and-29: comparison with type I interferons. *Cytokine* **31**, 109-118 (2005).
