# Supplementary material for: A systems immunology approach to investigate cytokine responses to viruses and bacteria and their association with disease
Source: Sci Rep. 2022 Aug 5;12:13463. doi: 10.1038/s41598-022-16509-4 (PMC9356009; doi:10.1038/s41598-022-16509-4)
Supplement: Supplementary file 3 — Supplementary Figure S3. [file 41598_2022_16509_MOESM3_ESM.pdf]

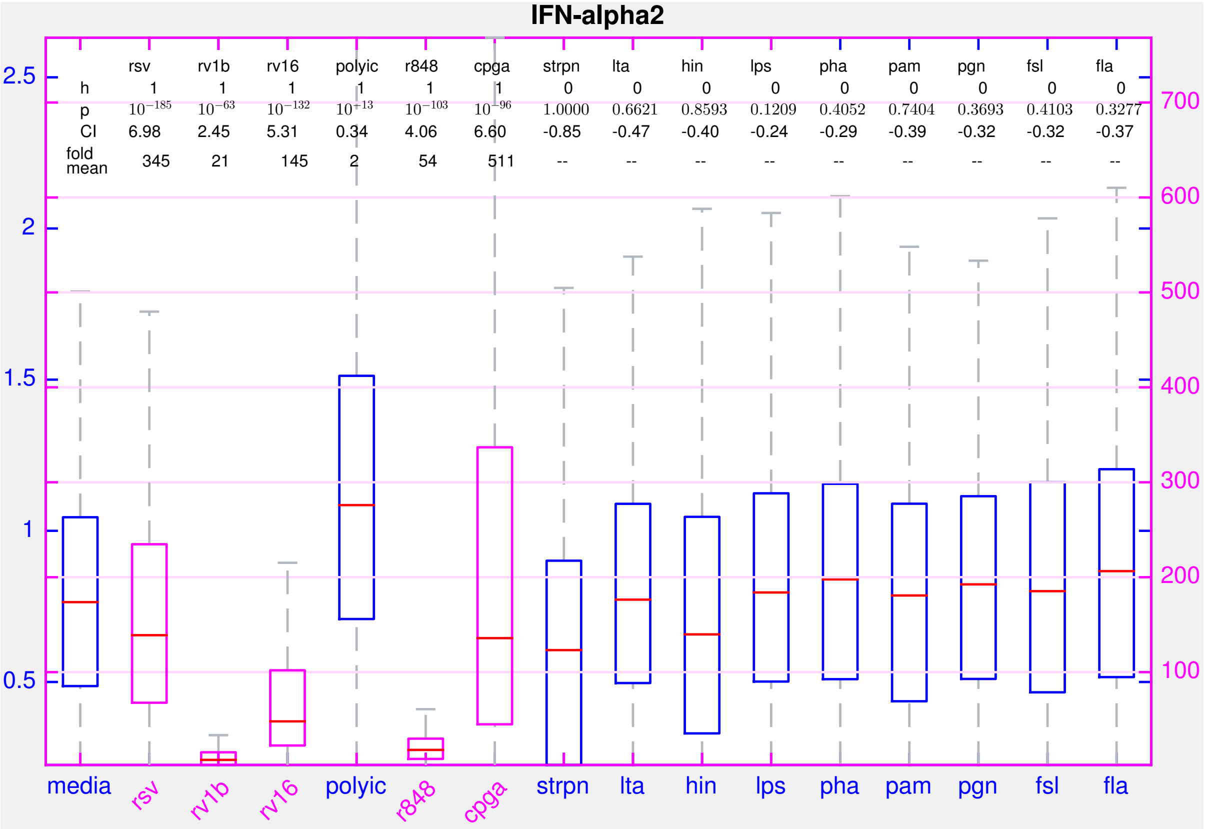

Fig. S3. Boxplots for absolute levels of 28 cytokines in pg/mL for medium control and each of the 15 stimuli shown as individual panels. (1) IFN-α2

Each panel presents one cytokine; when cytokine responses were highly variable among different stimuli, a right y-axis was needed with the corresponding stimuli using right y-axis indicated in pink. Each plot shows lower quartile (Q1, 25th percentile), median (red line), and upper quartile (Q3, 75th percentile). Interquartile range IQR = Q3-Q1 and values outside the range Q1-1.5 * IQR and Q3 +1.5 *IQR (where grey whiskers extended) were deemed outliers and omitted from the plot (except in IFN-beta where outliers are shown for the cases where IQR = 0). One-sided t-test was used to test if the cytokine was significantly induced by the stimulus: h=0 no; h=1 yes; p: p-value of the test; CI: lower bound in the 95% confidence interval; fold mean: robust mean (excluding outliers) of the corresponding fold induction.

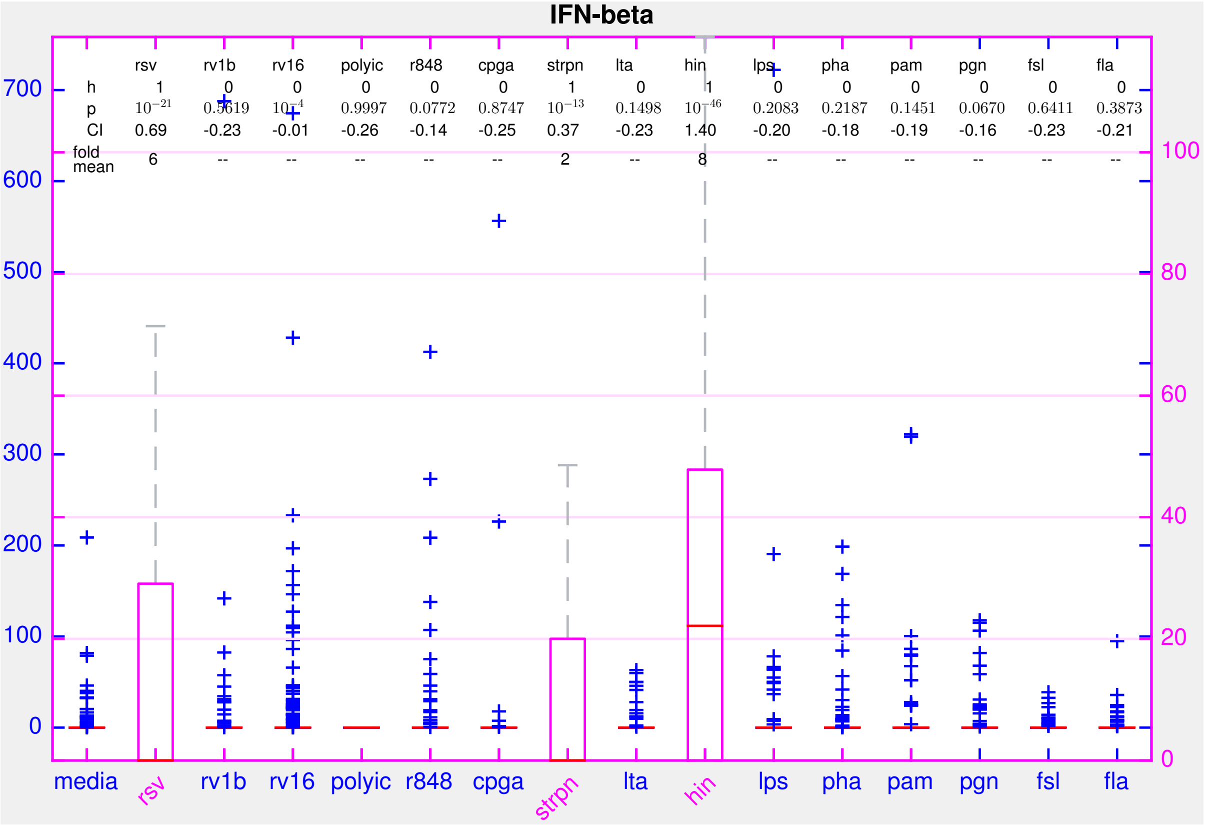

Figure S3. Boxplots for absolute levels of 28 cytokines in pg/mL for medium control and each of the 15 stimuli shown as individual panels. (2) IFN-β

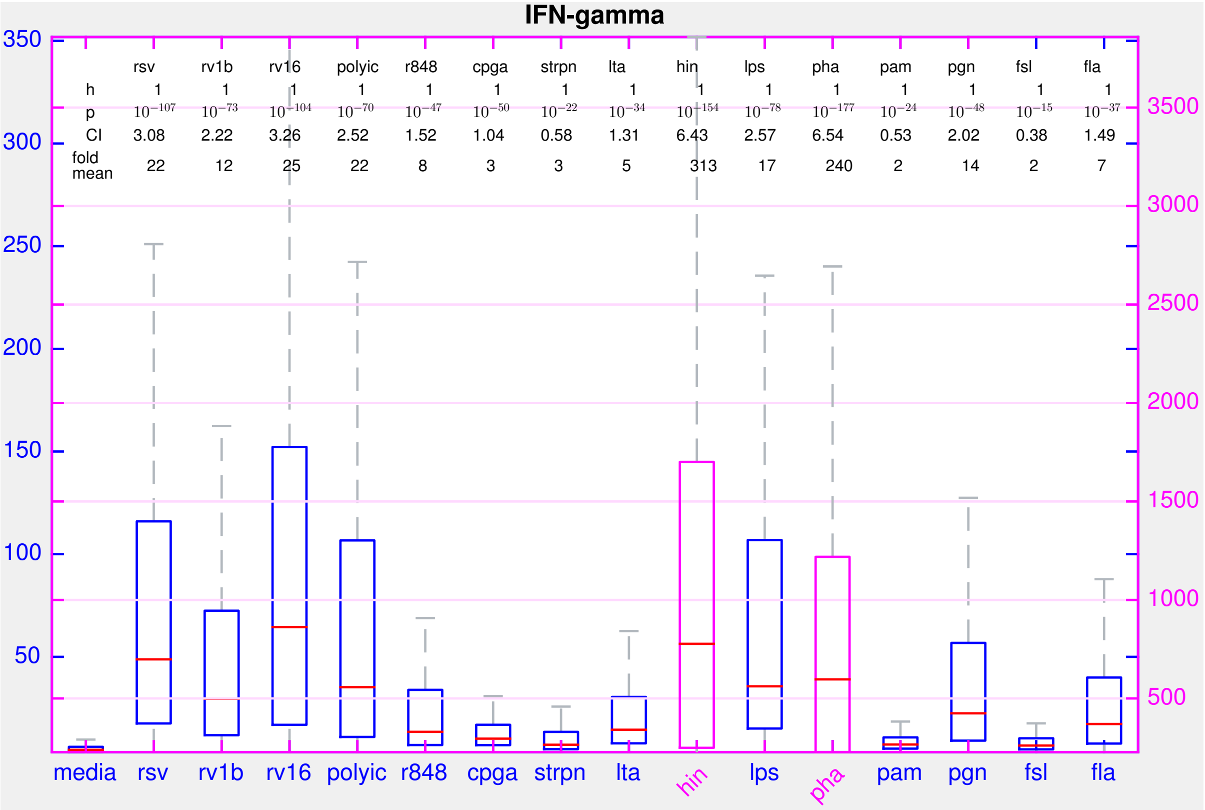

Figure S3. Boxplots for absolute levels of 28 cytokines in pg/mL for medium control and each of the 15 stimuli shown as individual panels. (3) IFN-γ

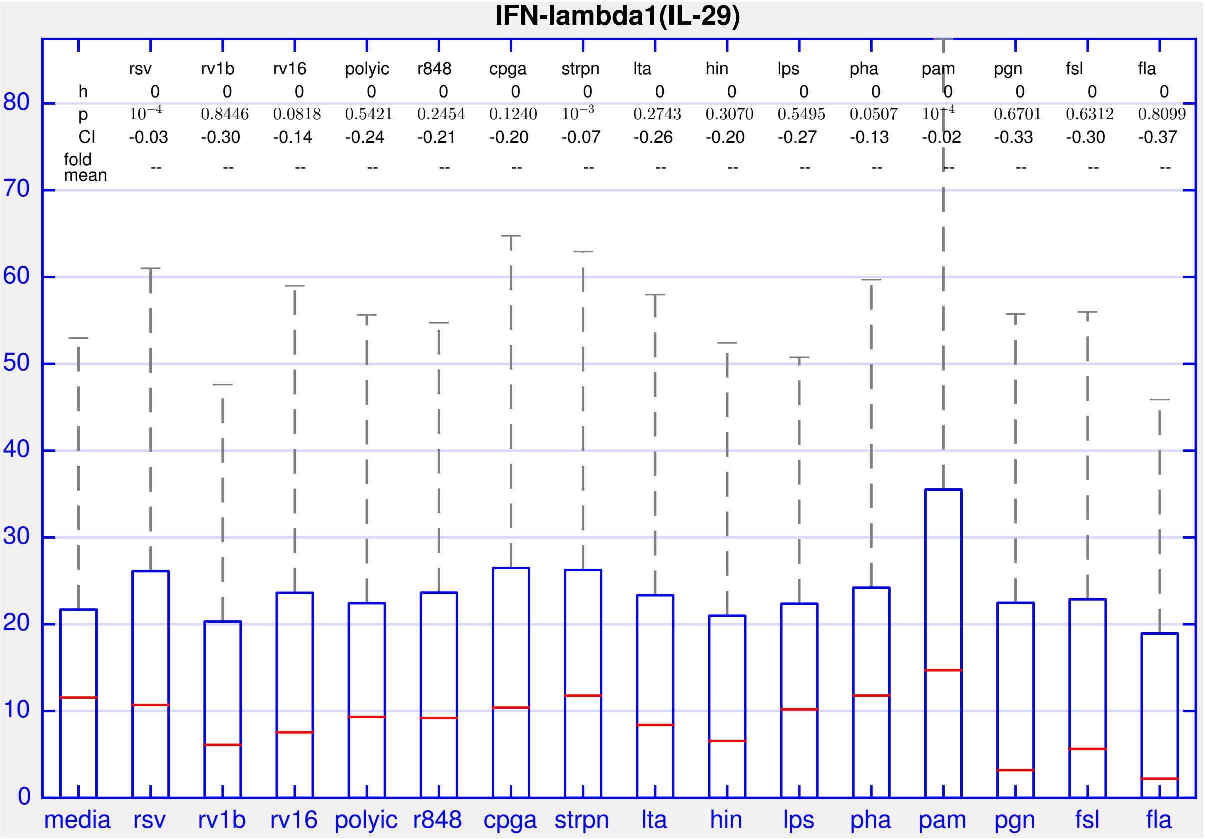

Figure S3. Boxplots for absolute levels of 28 cytokines in pg/mL for medium control and each of the 15 stimuli shown as individual panels. (4) IFN-λ1/IL-29

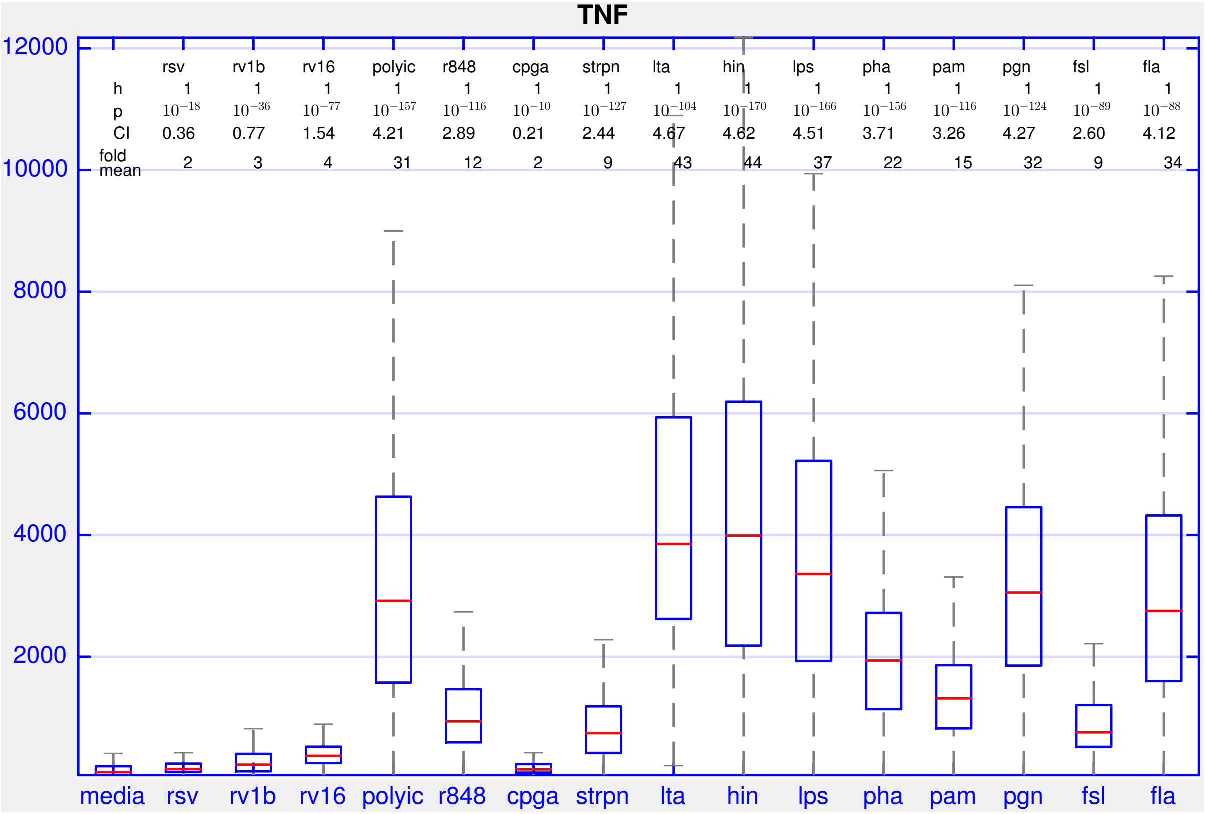

Figure S3. Boxplots for absolute levels of 28 cytokines in pg/mL for medium control and each of the 15 stimuli shown as individual panels. (5) TNF

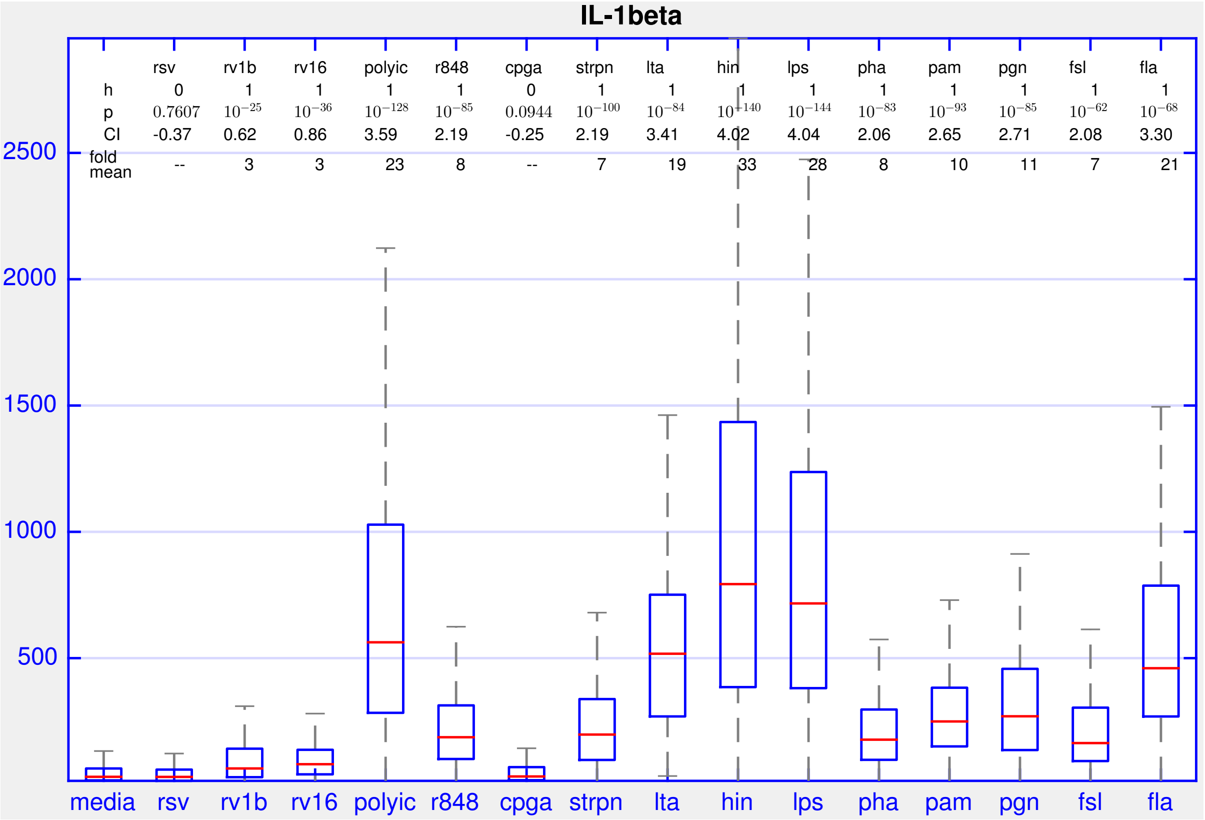

Figure S3. Boxplots for absolute levels of 28 cytokines in pg/mL for medium control and each of the 15 stimuli shown as individual panels. (6) IL-1β

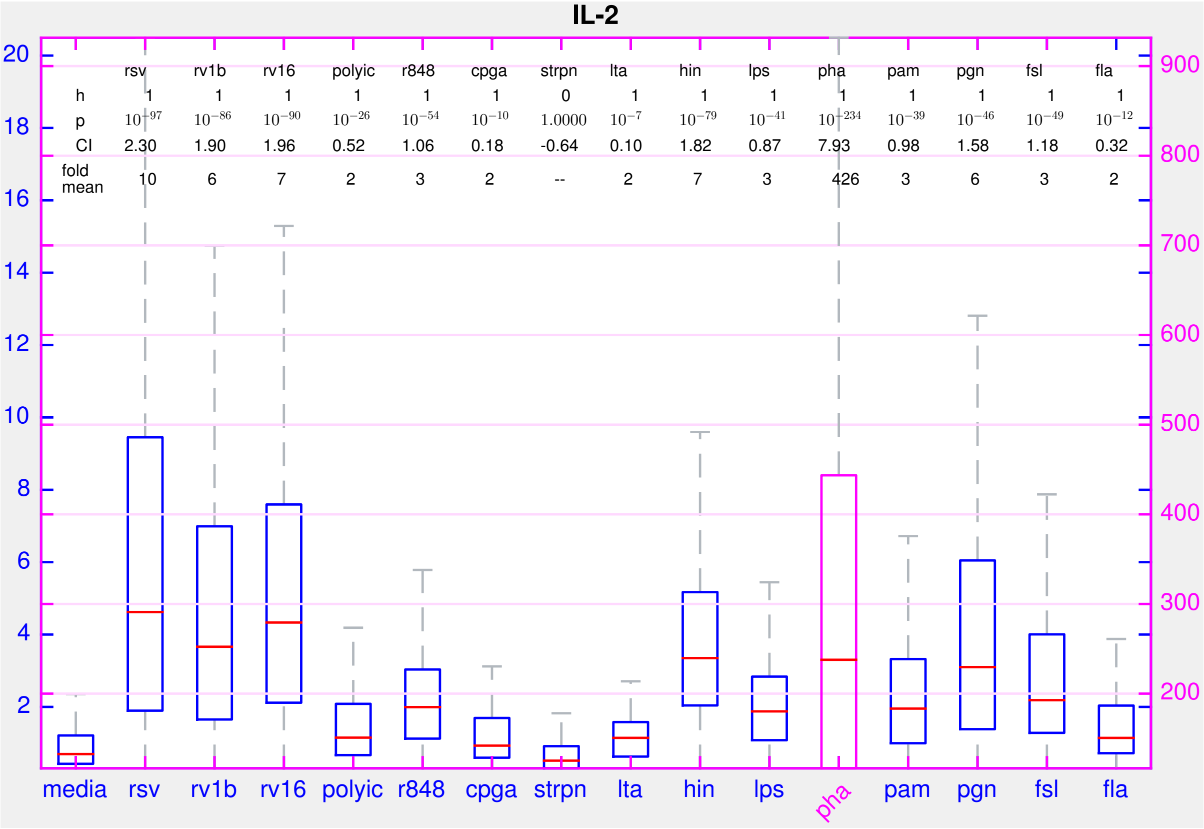

Figure S3. Boxplots for absolute levels of 28 cytokines in pg/mL for medium control and each of the 15 stimuli shown as individual panels. (7) IL-2

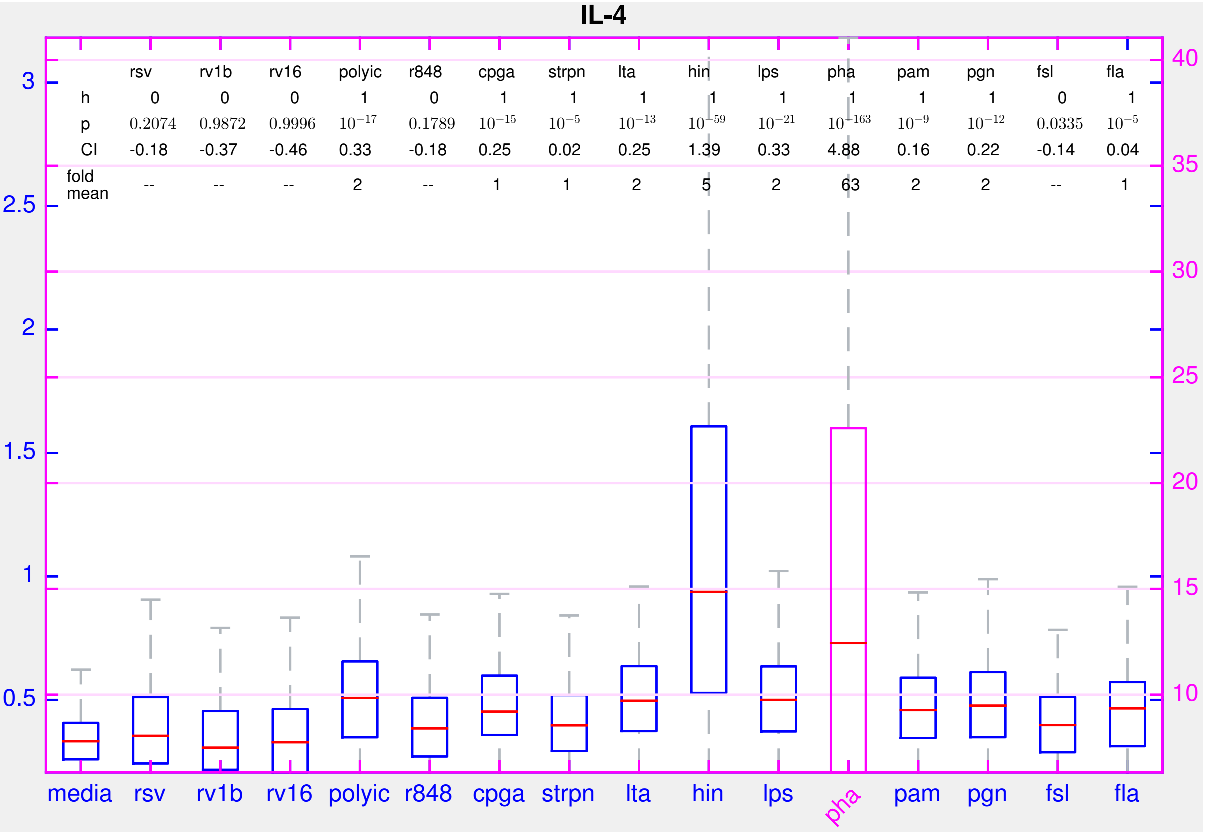

Figure S3. Boxplots for absolute levels of 28 cytokines in pg/mL for medium control and each of the 15 stimuli shown as individual panels. (8) IL-4

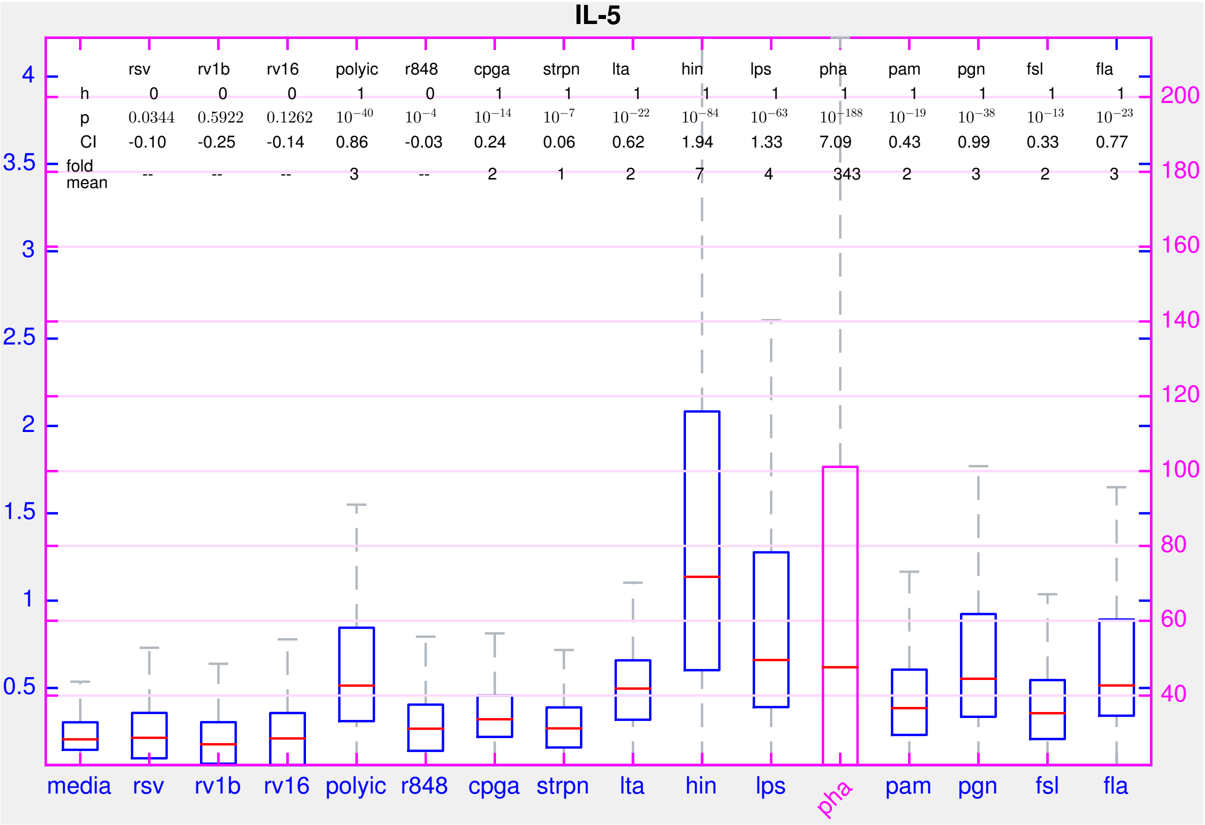

Figure S3. Boxplots for absolute levels of 28 cytokines in pg/mL for medium control and each of the 15 stimuli shown as individual panels. (9) IL-5

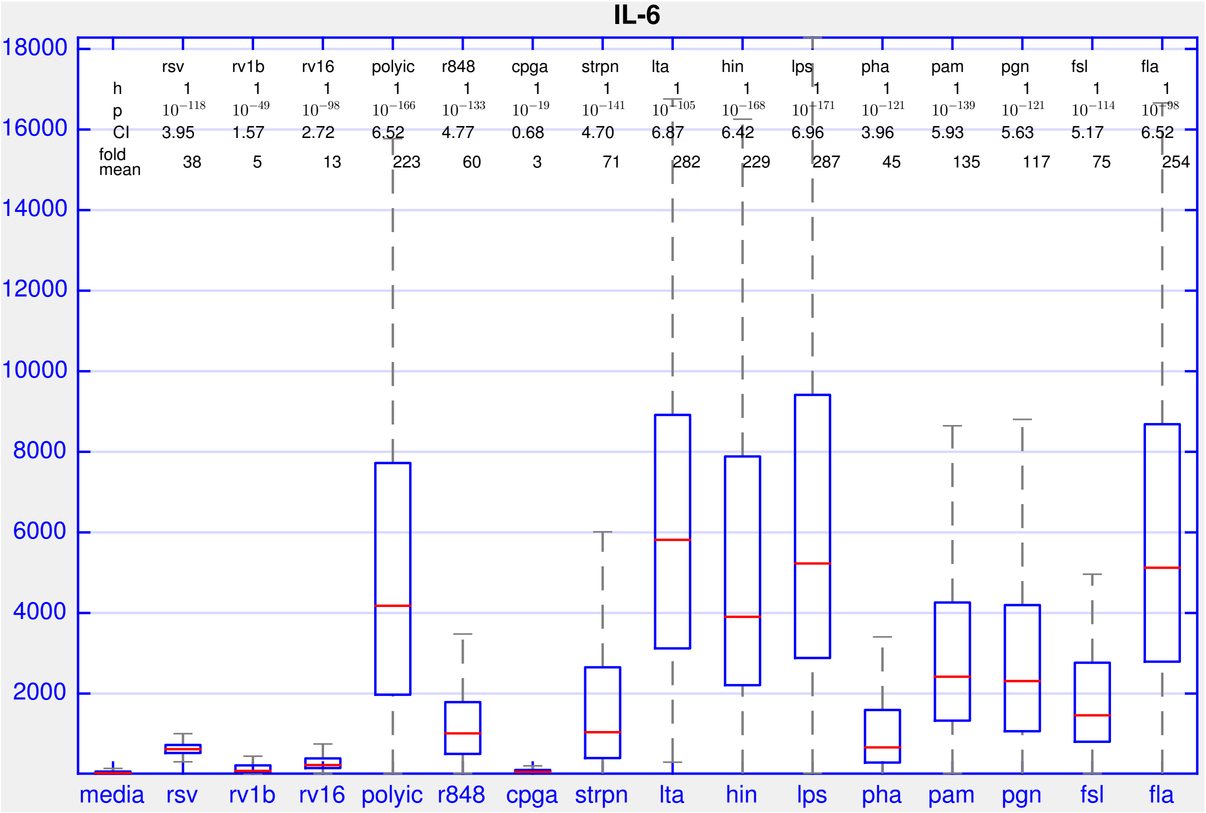

Figure S3. Boxplots for absolute levels of 28 cytokines in pg/mL for medium control and each of the 15 stimuli shown as individual panels. (10) IL-6

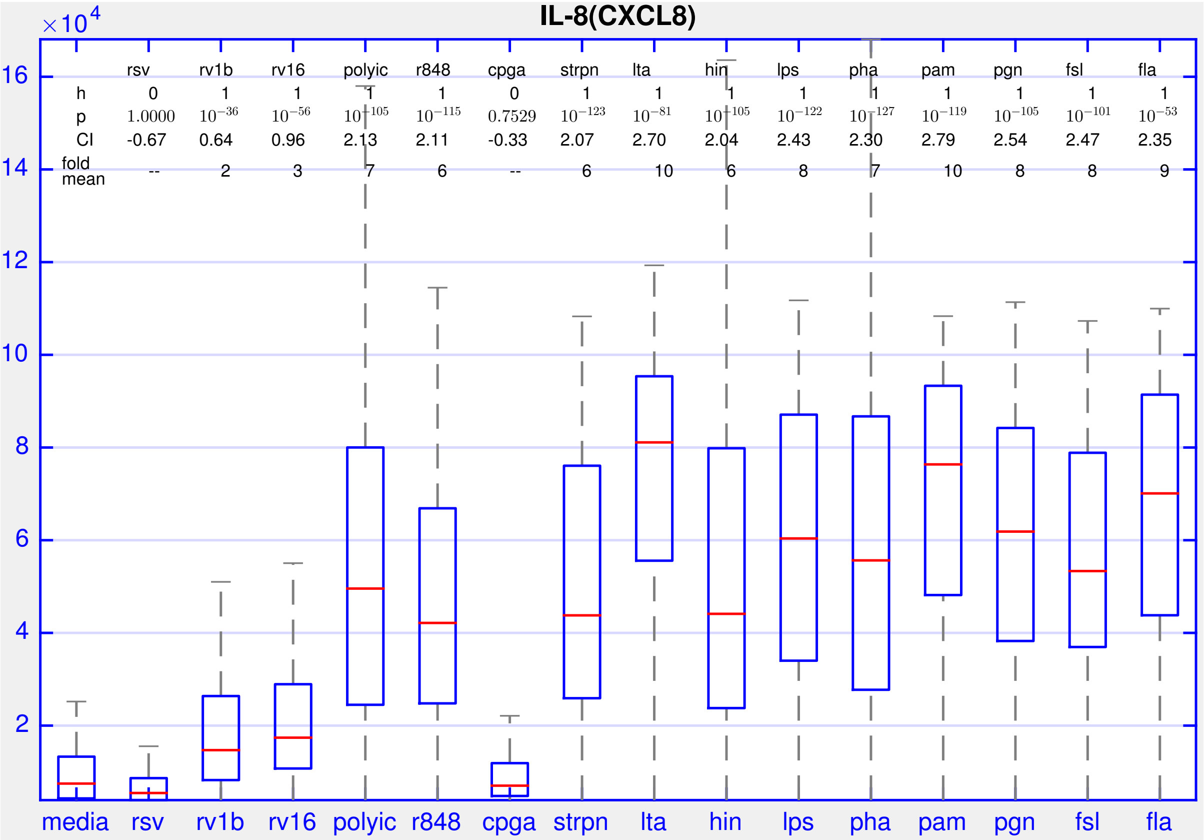

Figure S3. Boxplots for absolute levels of 28 cytokines in pg/mL for medium control and each of the 15 stimuli shown as individual panels. (11) IL-8/CXCL8

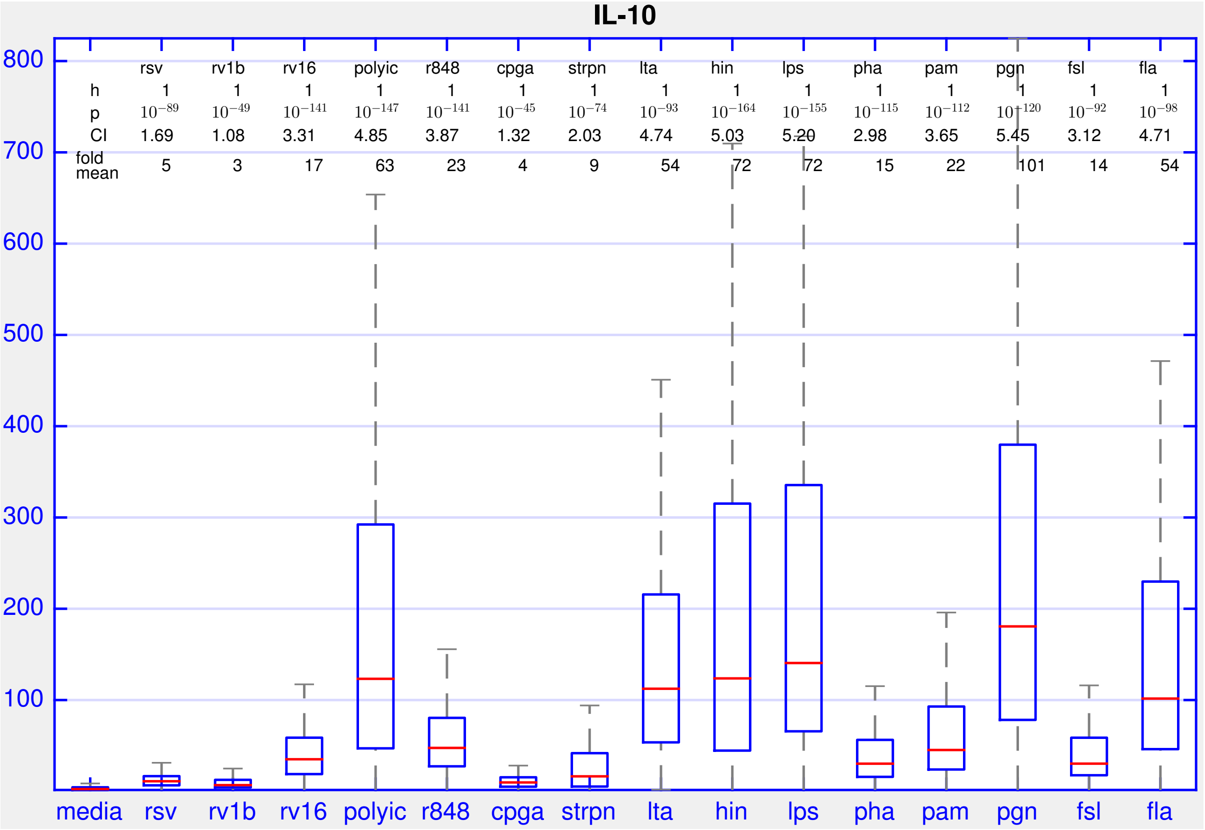

Figure S3. Boxplots for absolute levels of 28 cytokines in pg/mL for medium control and each of the 15 stimuli shown as individual panels. (12) IL-10

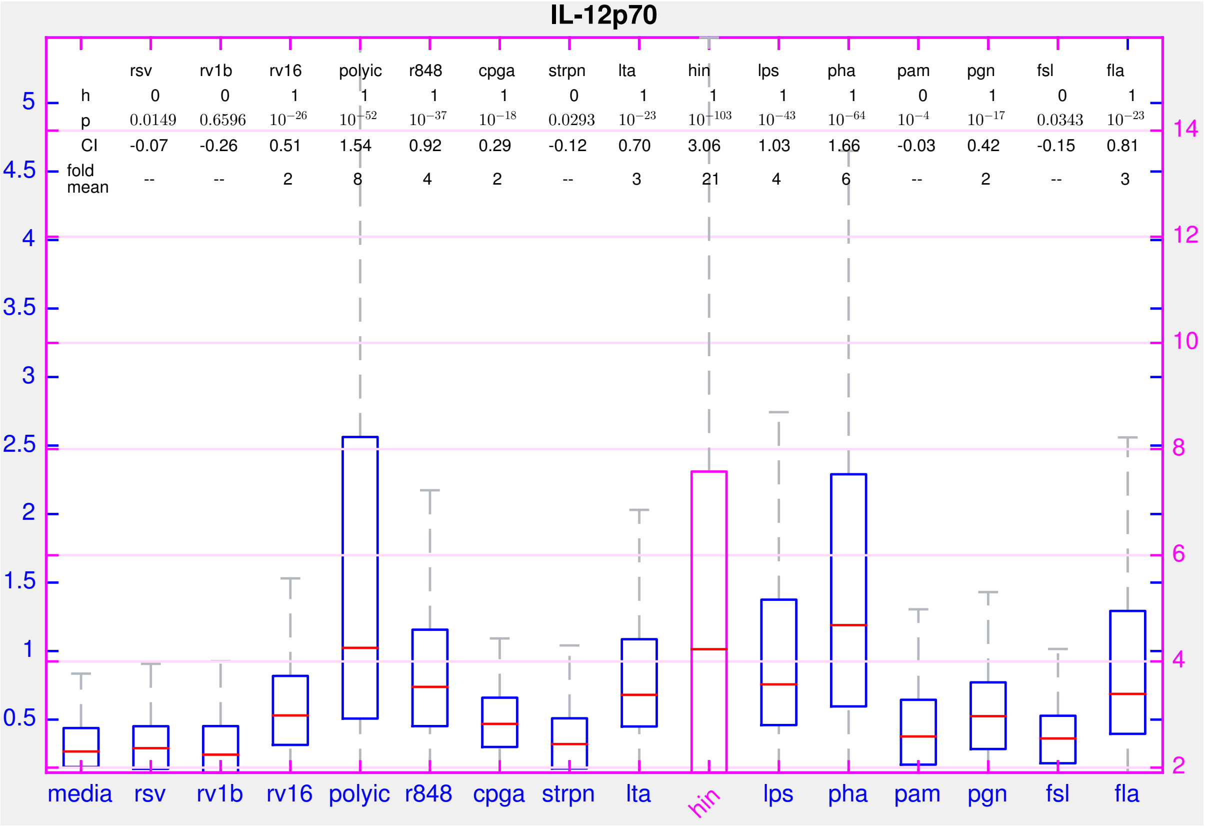

Figure S3. Boxplots for absolute levels of 28 cytokines in pg/mL for medium control and each of the 15 stimuli shown as individual panels. (13) IL-12p70

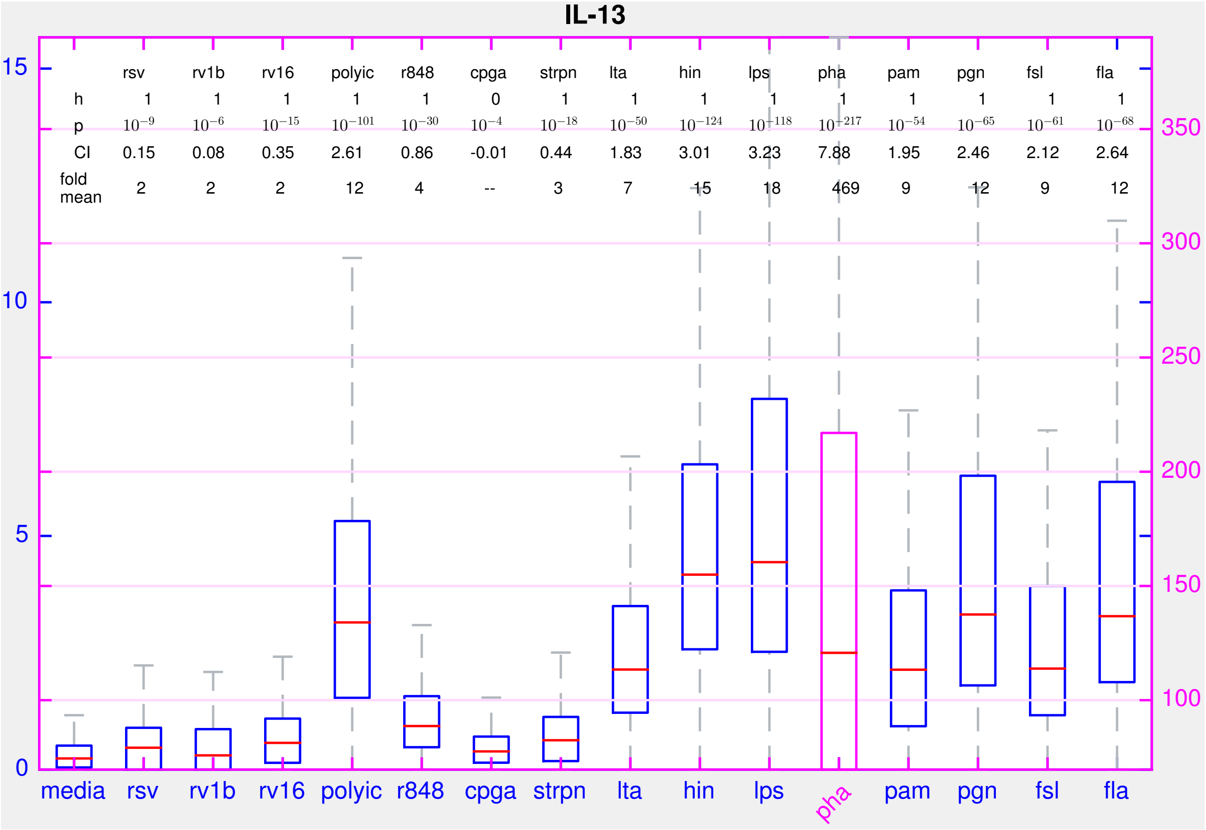

Figure S3. Boxplots for absolute levels of 28 cytokines in pg/mL for medium control and each of the 15 stimuli shown as individual panels. (14) IL-13

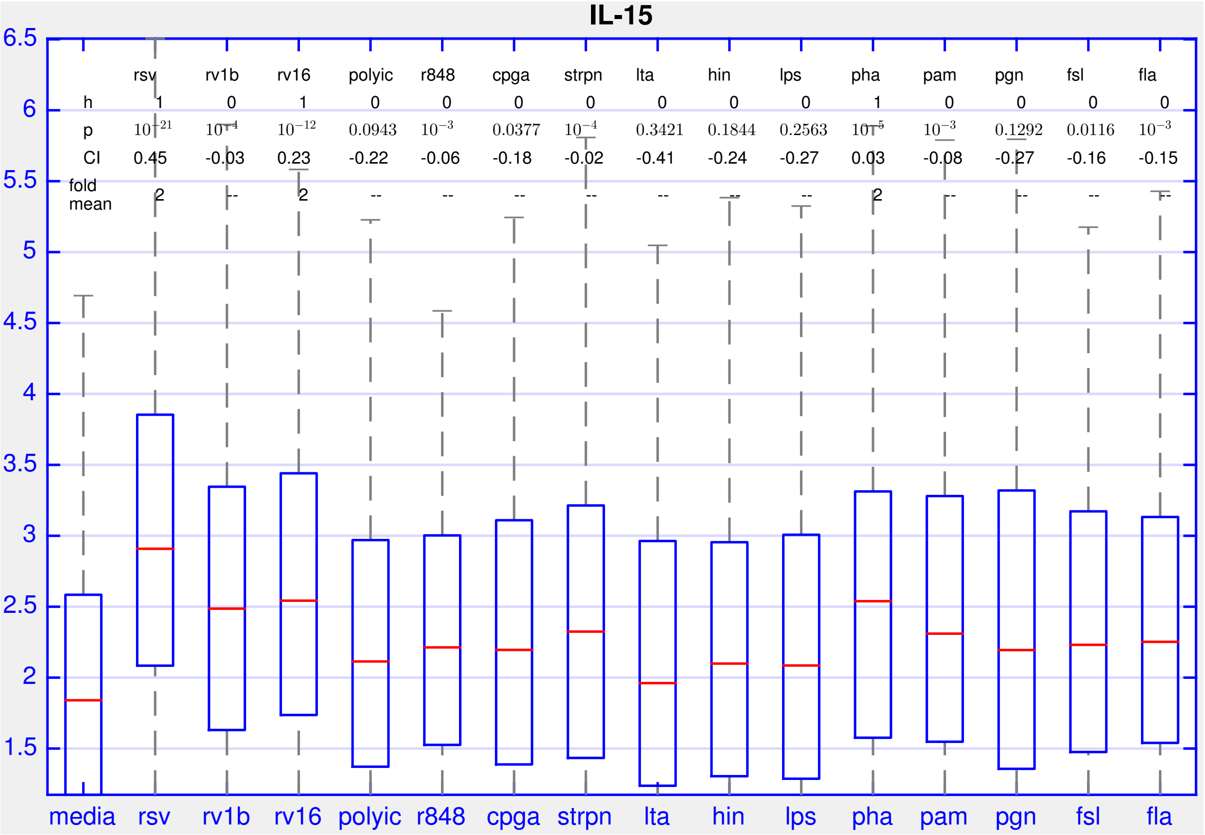

Figure S3. Boxplots for absolute levels of 28 cytokines in pg/mL for medium control and each of the 15 stimuli shown as individual panels. (15) IL-15

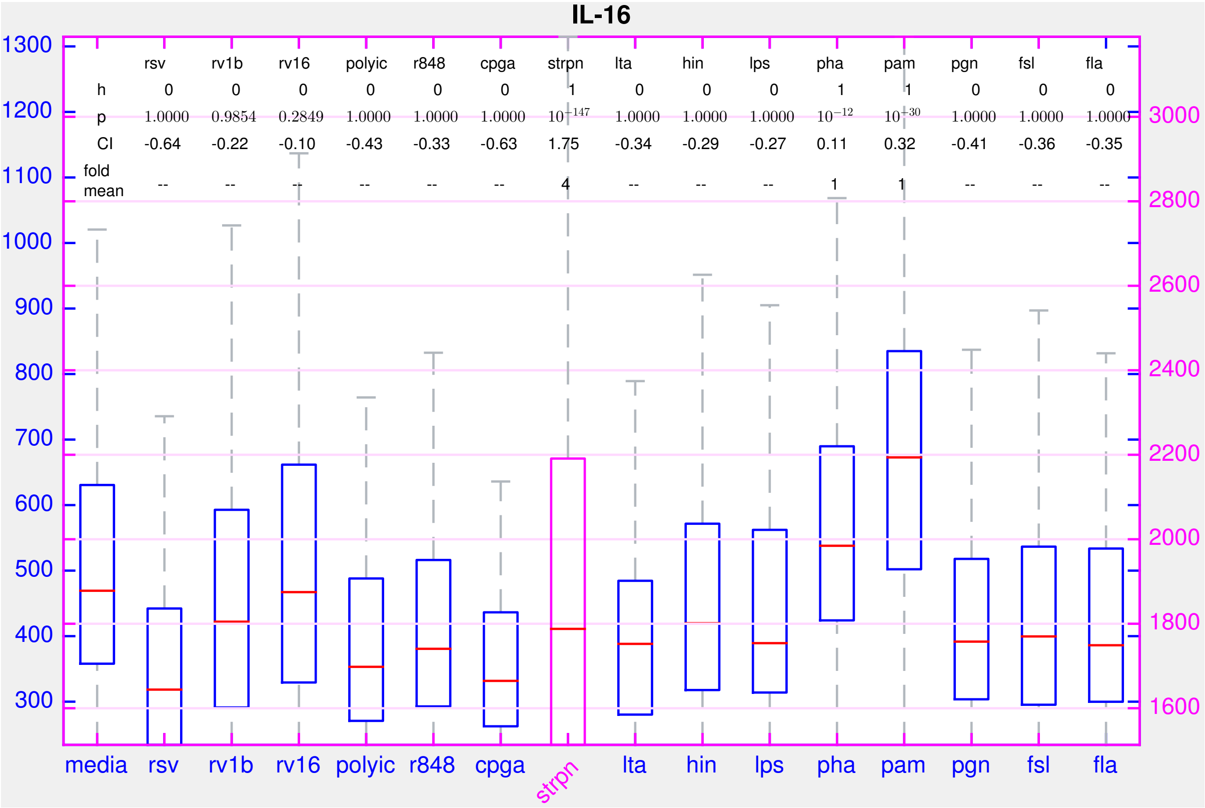

Figure S3. Boxplots for absolute levels of 28 cytokines in pg/mL for medium control and each of the 15 stimuli shown as individual panels. (16) IL-16

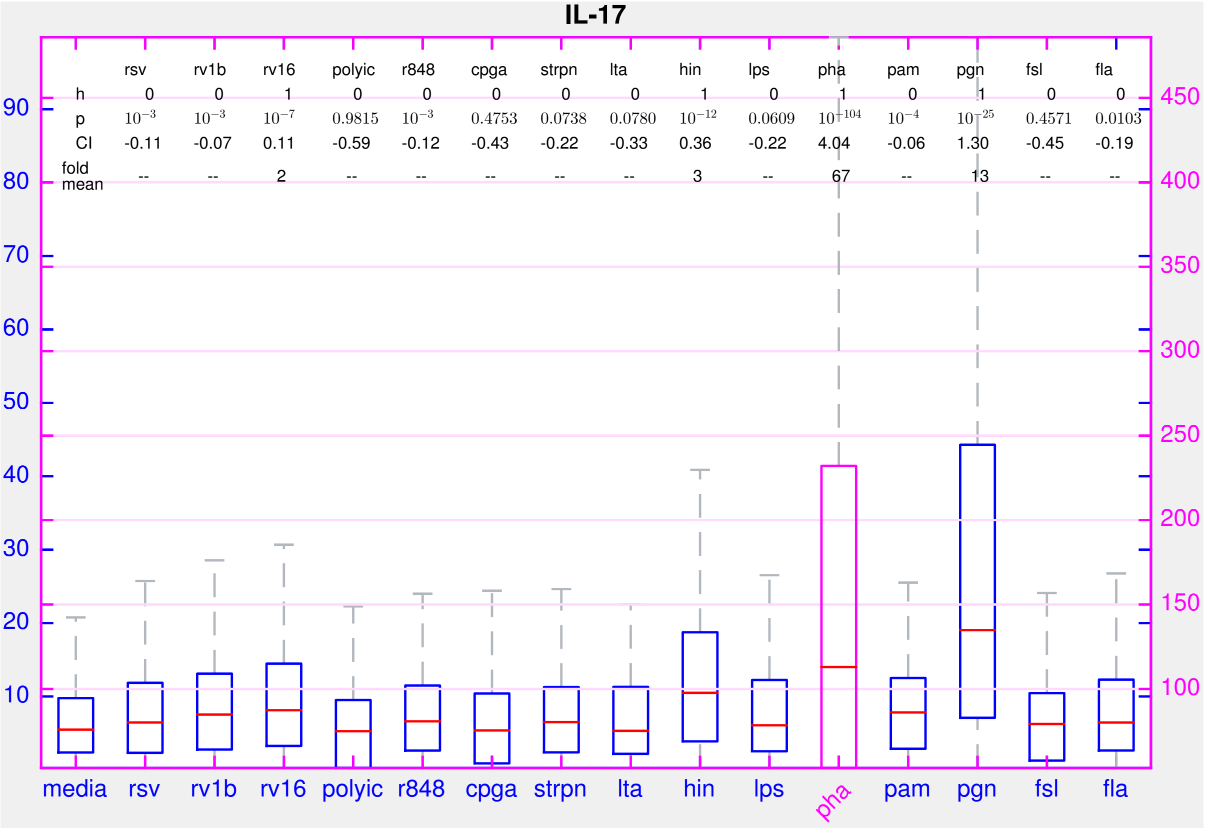

Figure S3. Boxplots for absolute levels of 28 cytokines in pg/mL for medium control and each of the 15 stimuli shown as individual panels. (17) IL-17

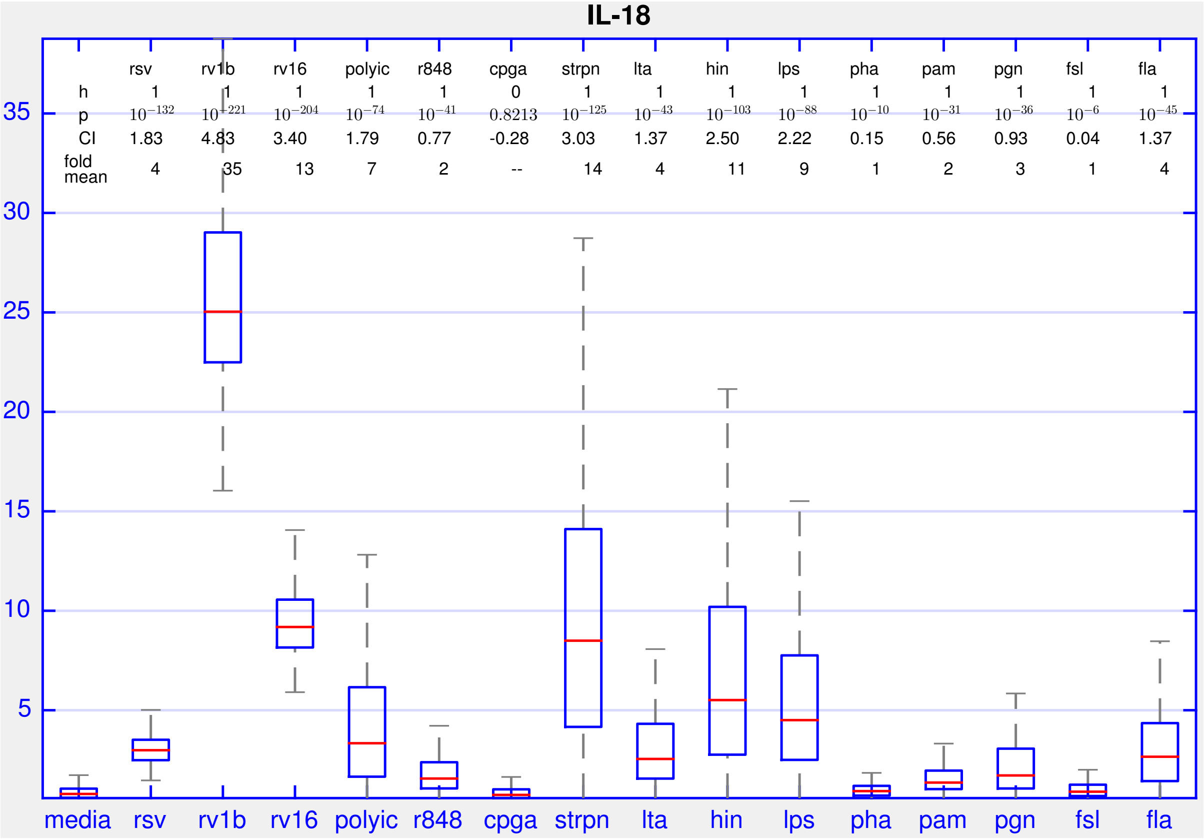

Figure S3. Boxplots for absolute levels of 28 cytokines in pg/mL for medium control and each of the 15 stimuli shown as individual panels. (18) IL-18

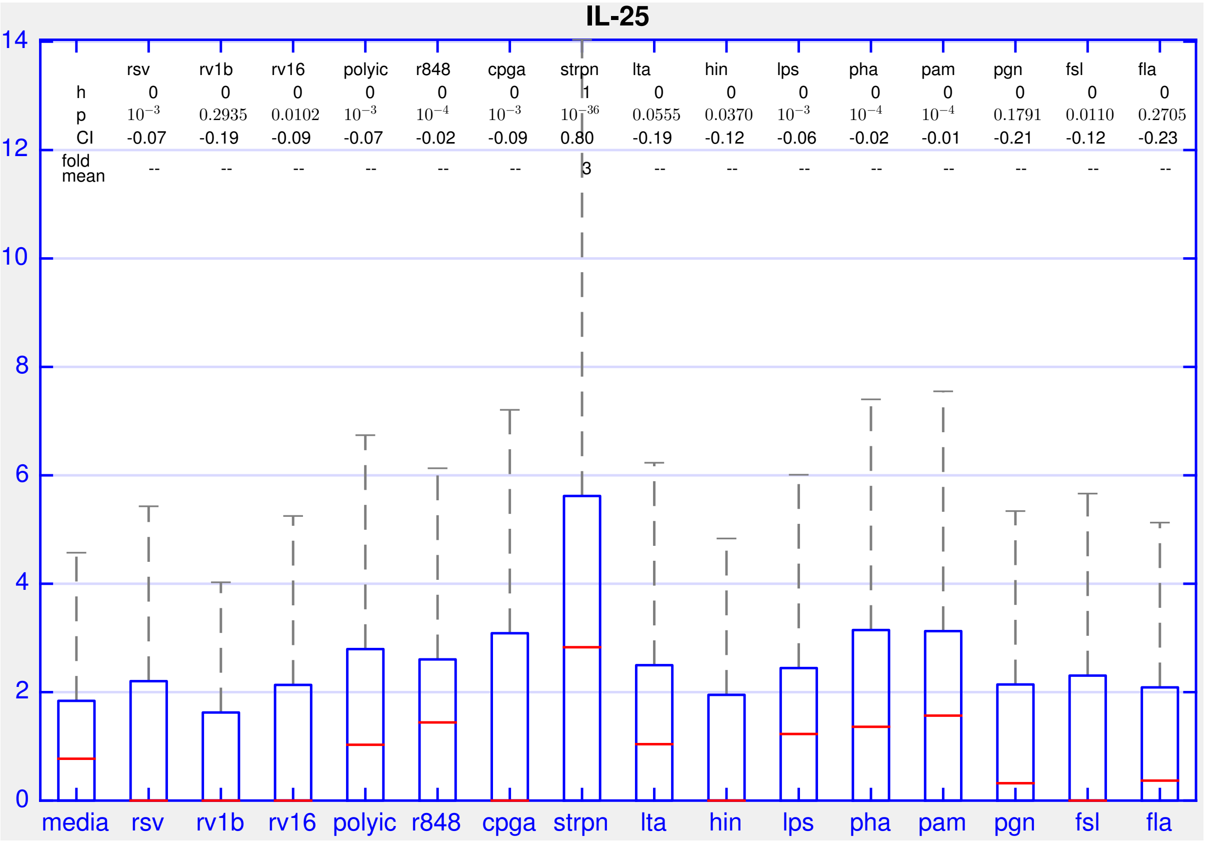

Figure S3. Boxplots for absolute levels of 28 cytokines in pg/mL for medium control and each of the 15 stimuli shown as individual panels. (19) IL-25

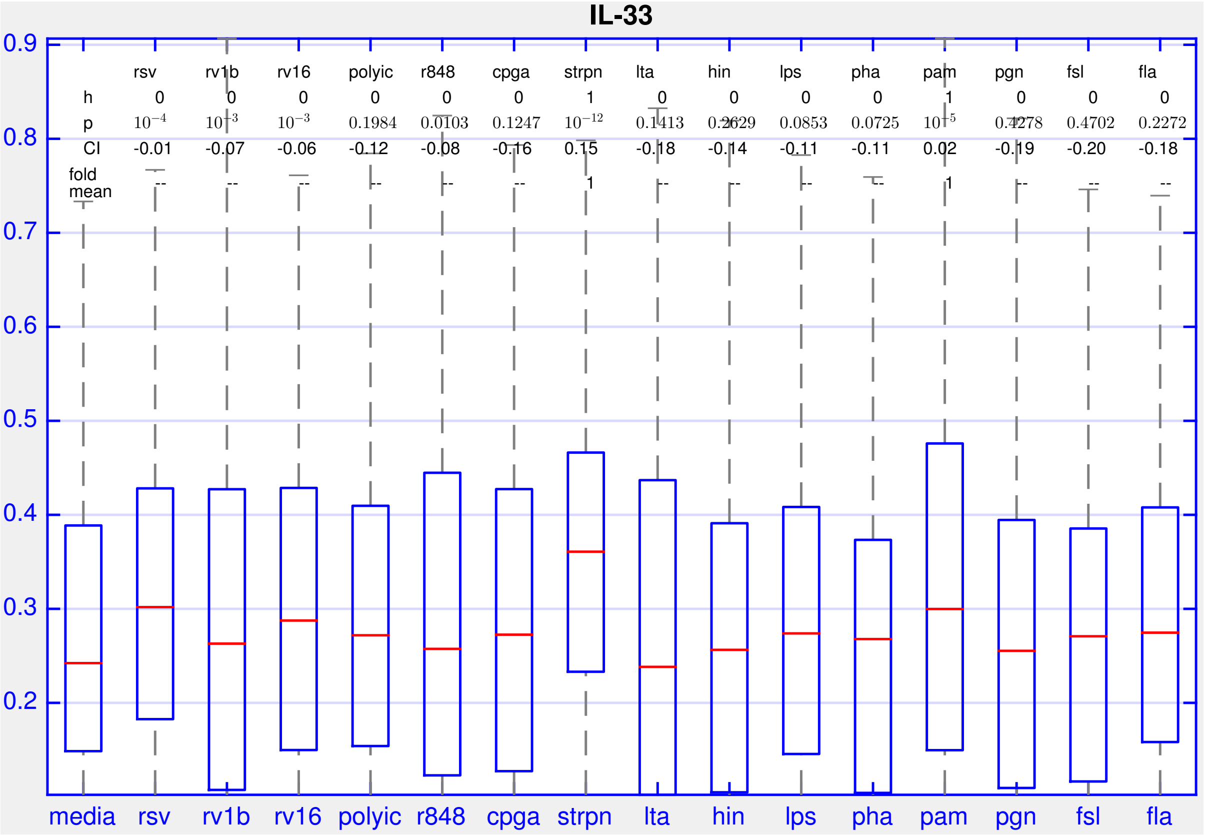

Figure S3. Boxplots for absolute levels of 28 cytokines in pg/mL for medium control and each of the 15 stimuli shown as individual panels. (20) IL-33

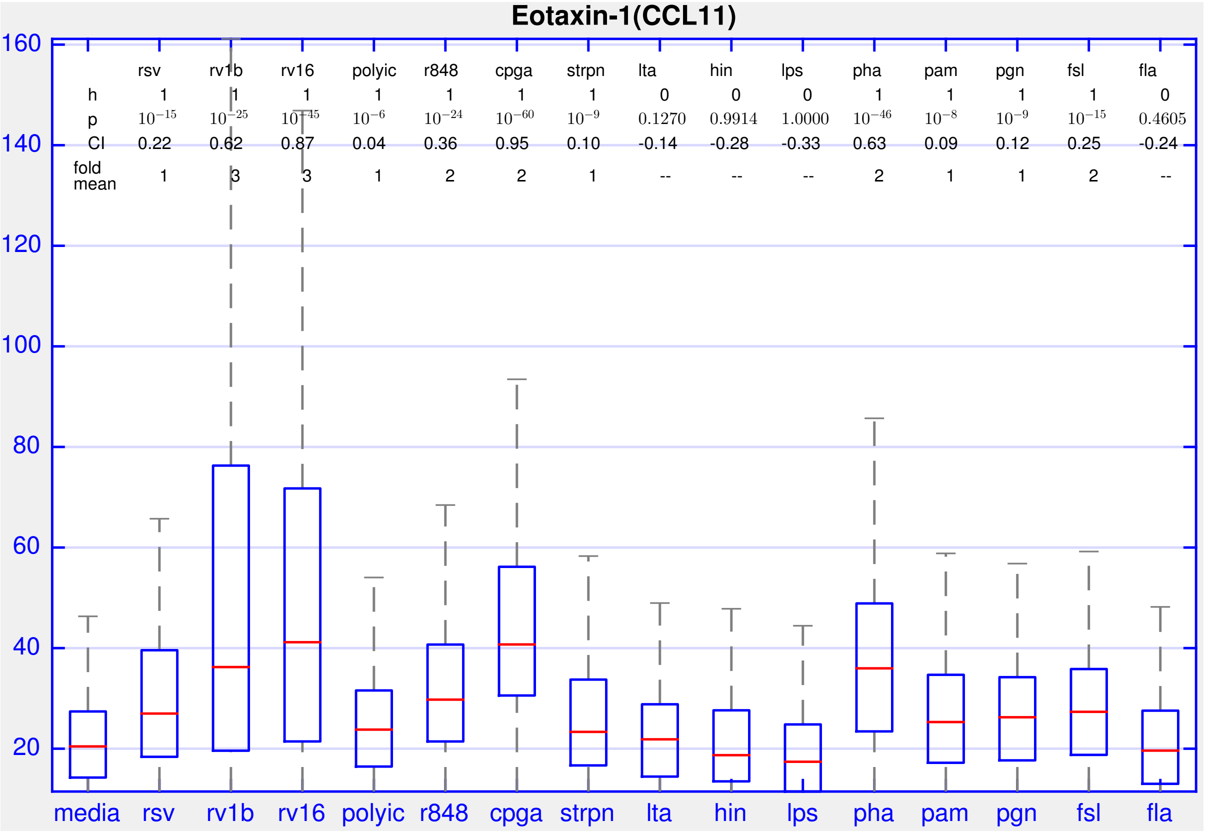

Figure S3. Boxplots for absolute levels of 28 cytokines in pg/mL for medium control and each of the 15 stimuli shown as individual panels. (21) Eotaxin-1/CCL11

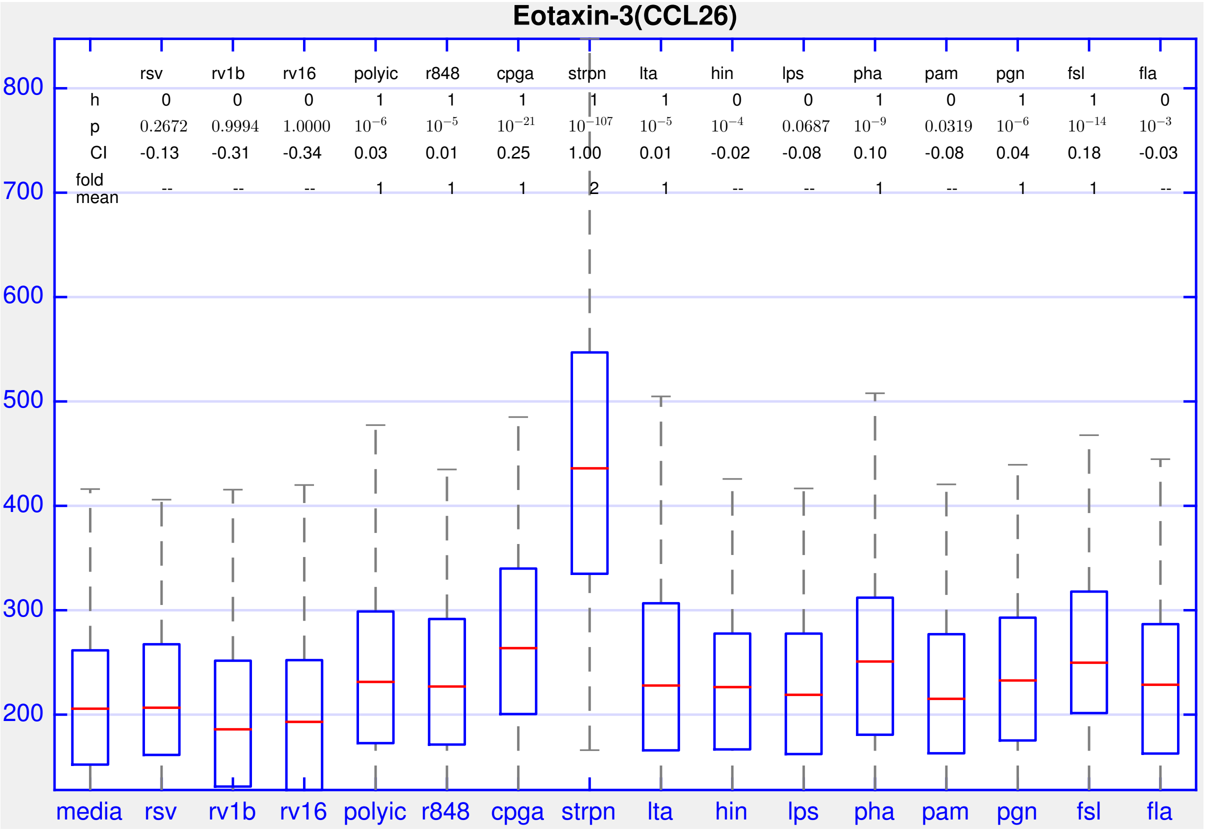

Figure S3. Boxplots for absolute levels of 28 cytokines in pg/mL for medium control and each of the 15 stimuli shown as individual panels. (22) Eotaxin-3/CCL26

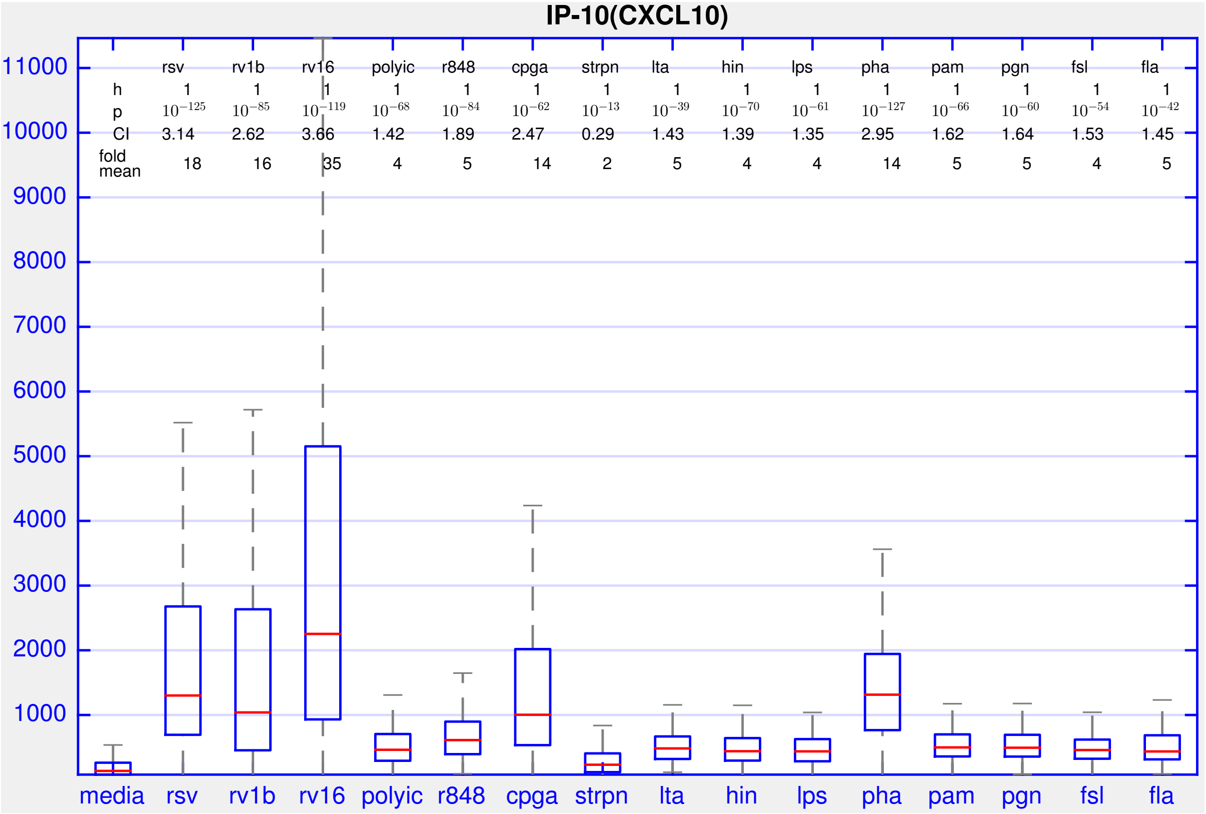

Figure S3. Boxplots for absolute levels of 28 cytokines in pg/mL for medium control and each of the 15 stimuli shown as individual panels. (23) IP-10/CXCL10

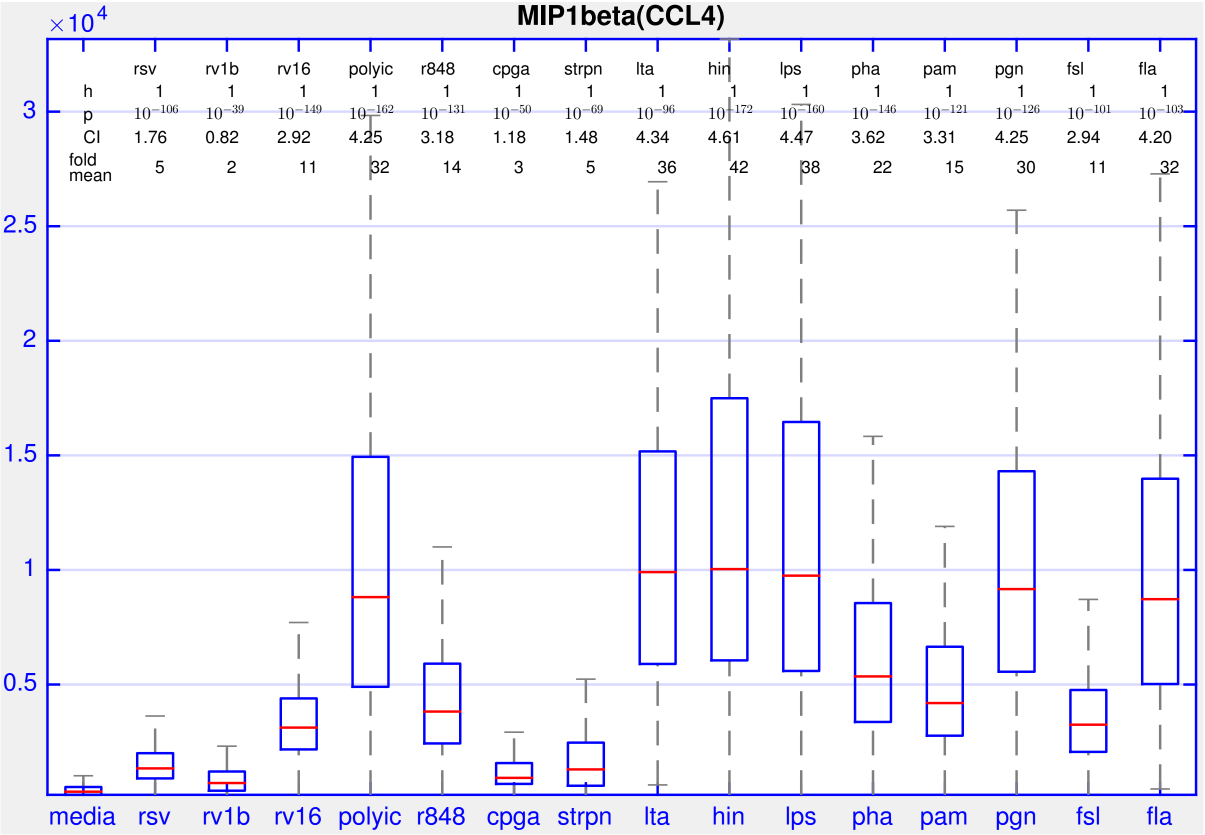

Figure S3. Boxplots for absolute levels of 28 cytokines in pg/mL for medium control and each of the 15 stimuli shown as individual panels. (24) MIP1β/CCL4

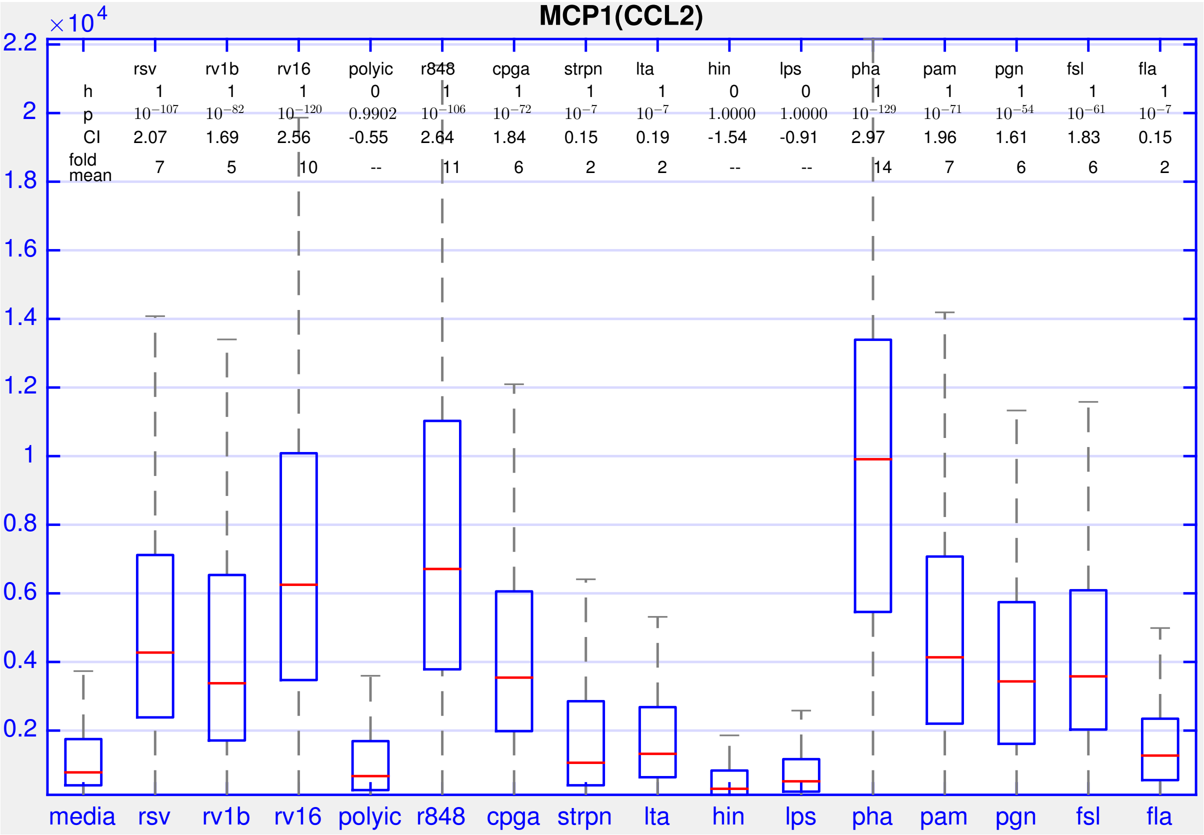

Figure S3. Boxplots for absolute levels of 28 cytokines in pg/mL for medium control and each of the 15 stimuli shown as individual panels. (25) MCP1/CCL2

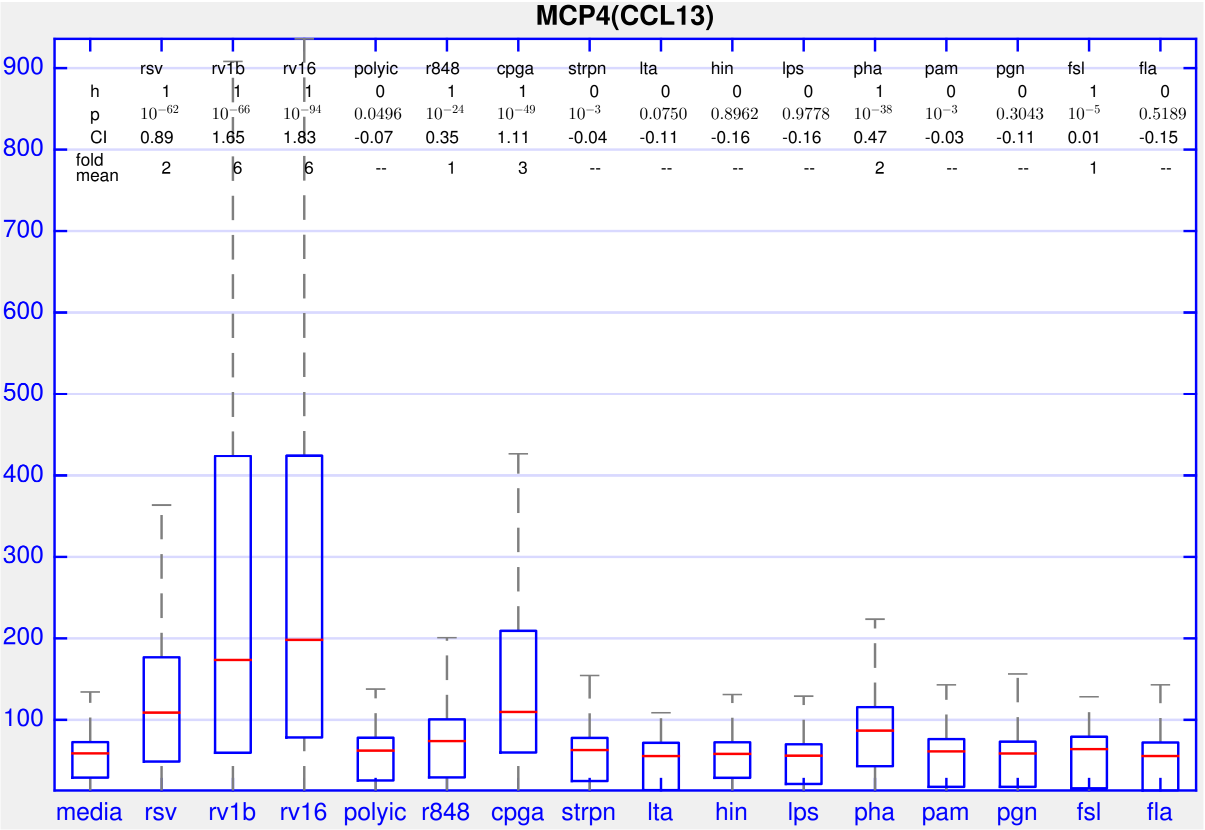

Figure S3. Boxplots for absolute levels of 28 cytokines in pg/mL for medium control and each of the 15 stimuli shown as individual panels. (26) MCP4/CCL13

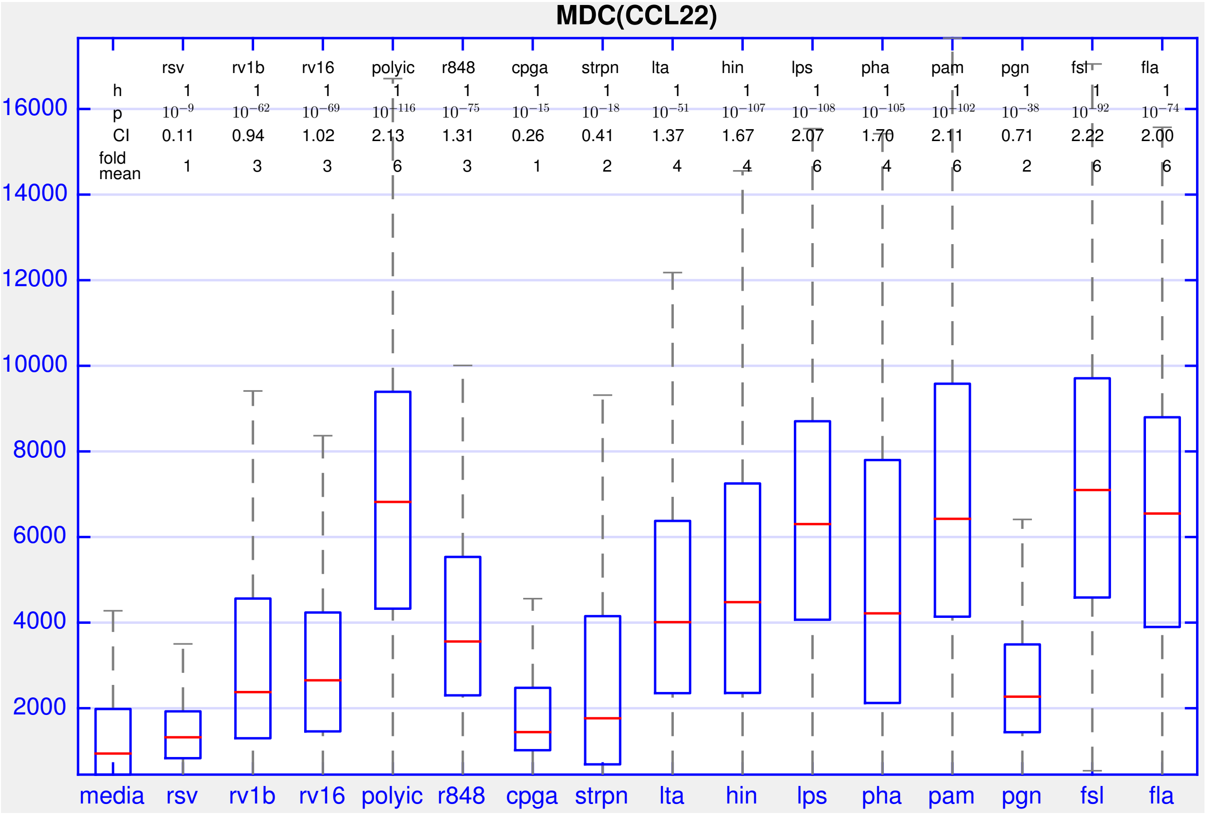

Figure S3. Boxplots for absolute levels of 28 cytokines in pg/mL for medium control and each of the 15 stimuli shown as individual panels. (27) MDC/CCL22

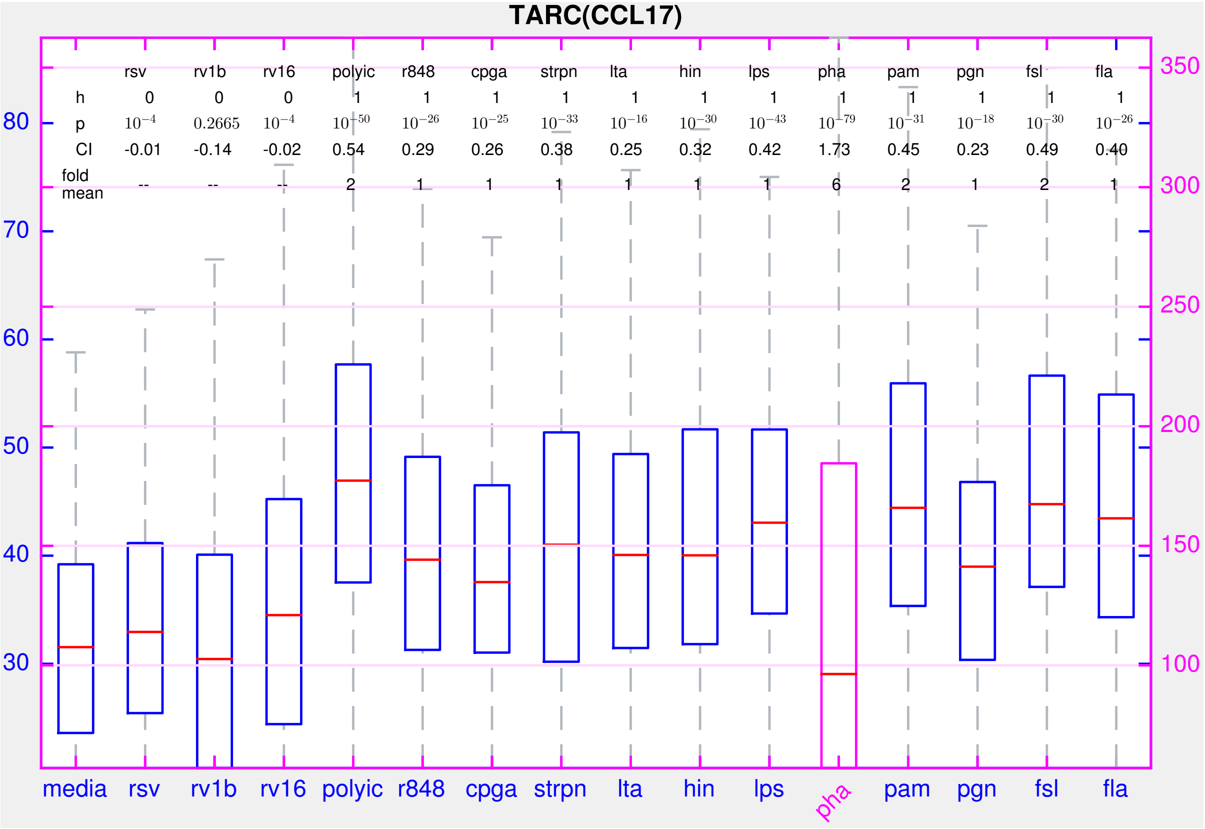

Figure S3. Boxplots for absolute levels of 28 cytokines in pg/mL for medium control and each of the 15 stimuli shown as individual panels. (28) TARC/CCL17
