# Supplementary material for: A systems immunology approach to investigate cytokine responses to viruses and bacteria and their association with disease
Source: Sci Rep. 2022 Aug 5;12:13463. doi: 10.1038/s41598-022-16509-4 (PMC9356009; doi:10.1038/s41598-022-16509-4)
Supplement: Supplementary file 4 — Supplementary Figure S8. [file 41598_2022_16509_MOESM4_ESM.pdf]

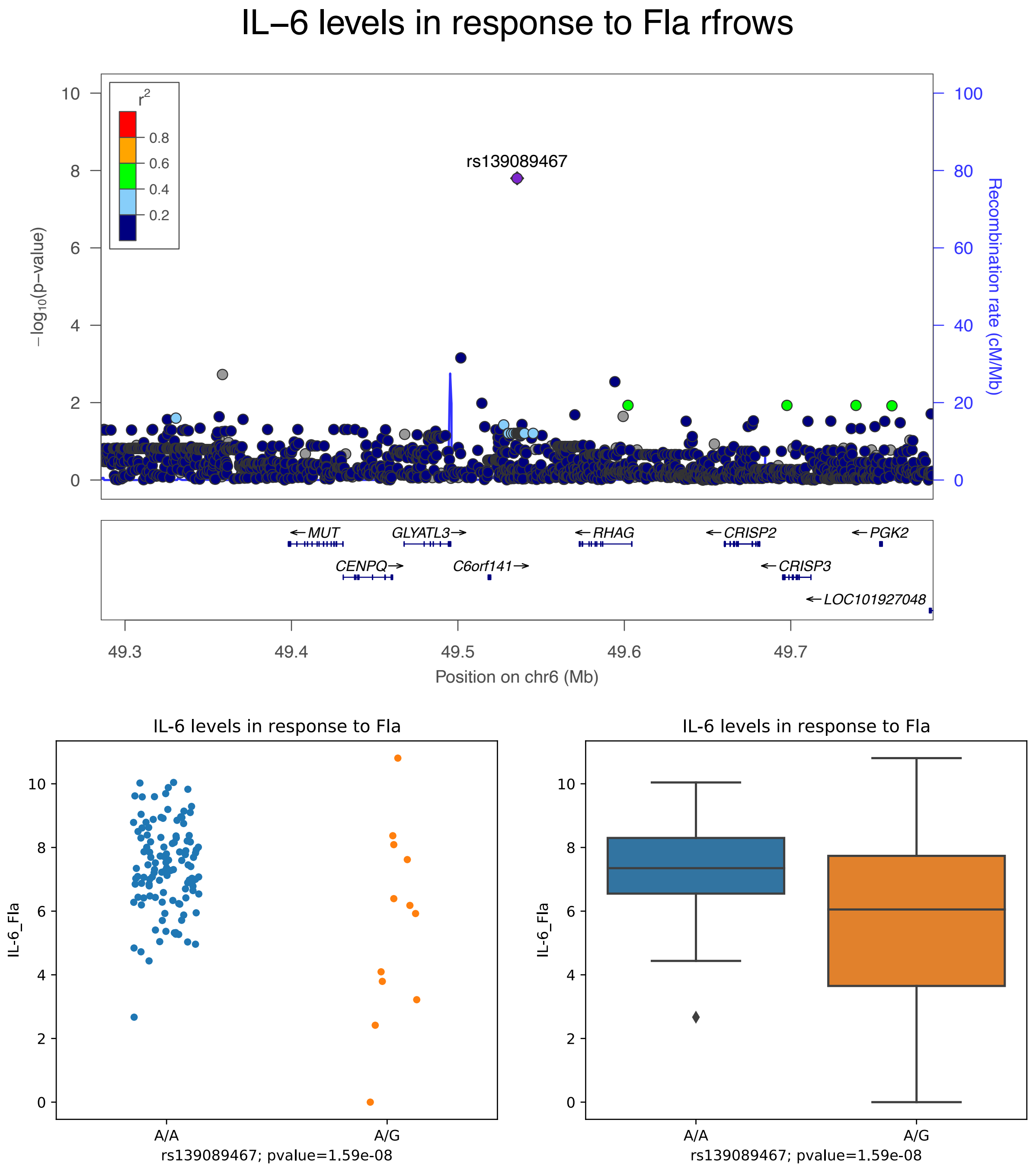

Fig. S8. Locuszoom Plots and IL-6 levels by genotypes for cQTL. (a) genotype rs139089467 (associated with IL-6 levels in response to Fla).

A locuszoom plot for each cQTL as well as IL-6 levels by genotype is provided for rs139089467 (IL-6 levels in response to Fla), rs117007889 (IL-6 levels in response to LTA), rs111481643 (IL-6 levels in response to LPS), rs77609006 (IL-6 levels in response to LTA), rs73624755 (IL-6 levels in response to LTA), rs73624755 (IL-6 levels in response to Fla). rs8028121 (IL-6 levels in response to Fla), rs7440580 (IL-6 levels in response to Hin). IL-6 levels by genotype for chr7:67342638:D and chr2:221028028:I (associated with IL-6 levels in response to FLS and Fla respectively) are also provided but it was not possible to generate locuszoom plots for these two SNPs.

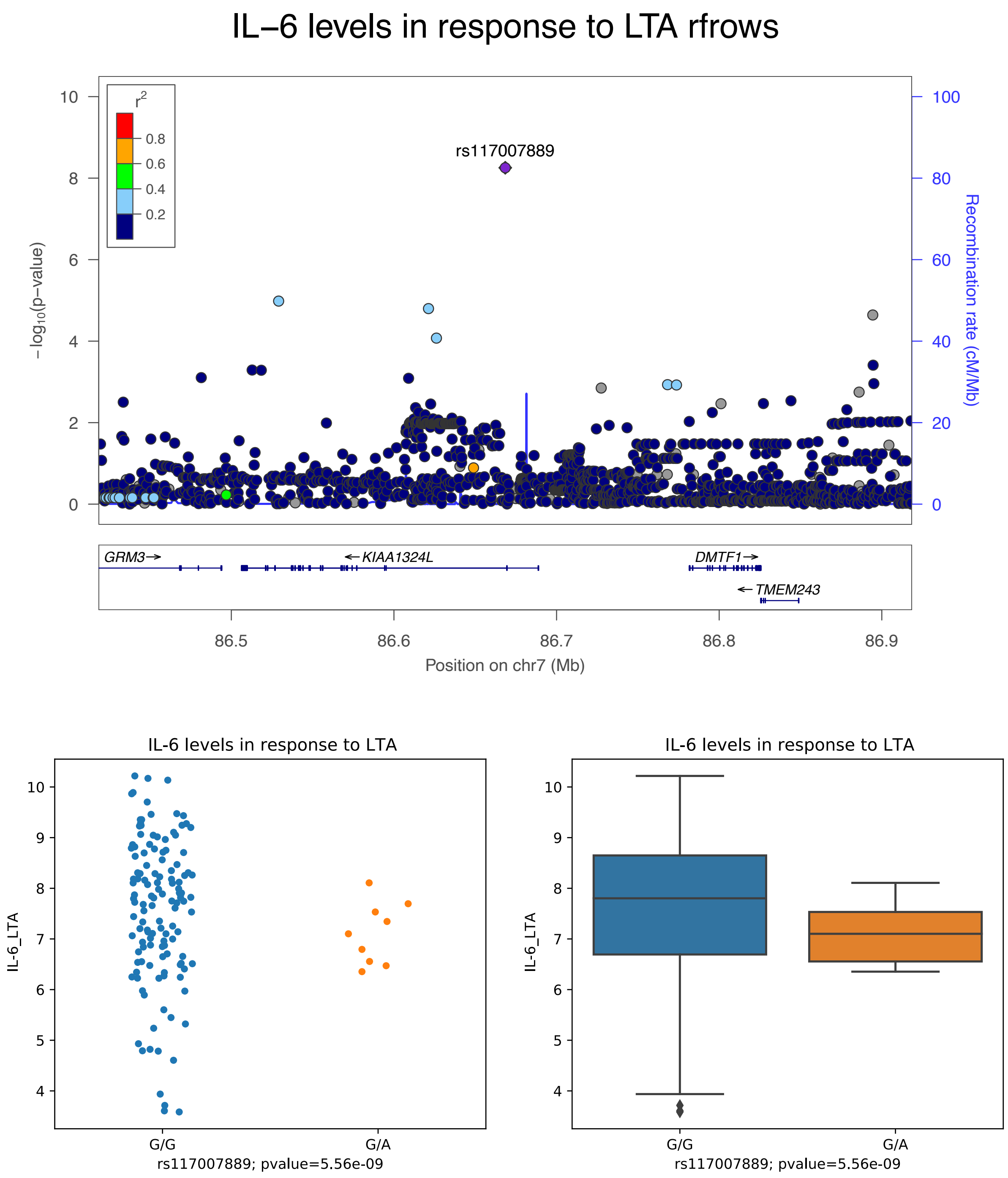

Fig. S8 Locuszoom Plots and IL-6 levels by genotypes for cQTL. (b) genotype rs117007889 (associated with IL-6 levels in response to LTA).

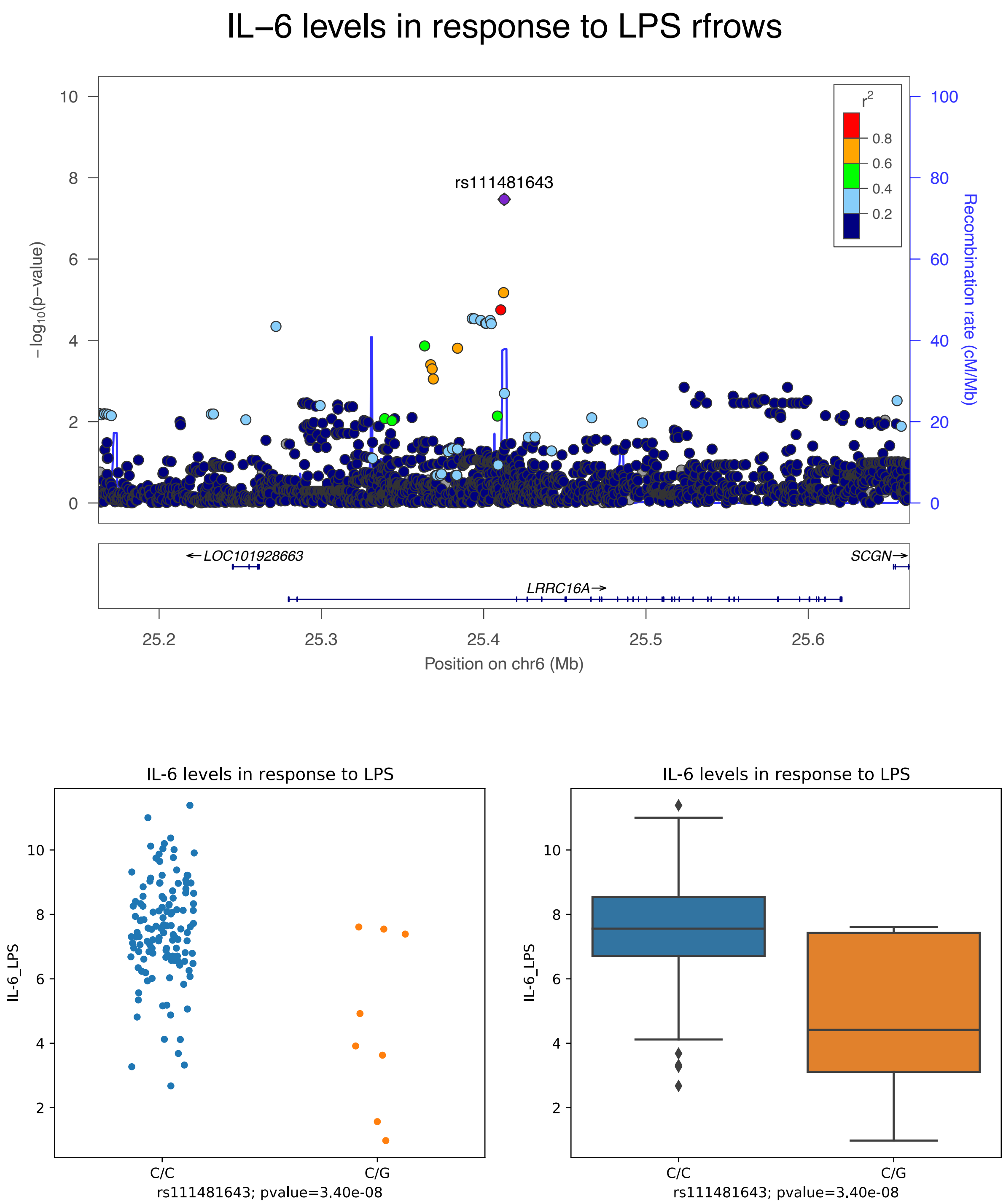

Fig. S8 Locuszoom Plots and IL-6 levels by genotypes for cQTL. (c) genotype rs111481643 (associated with IL-6 levels in response to LPS).

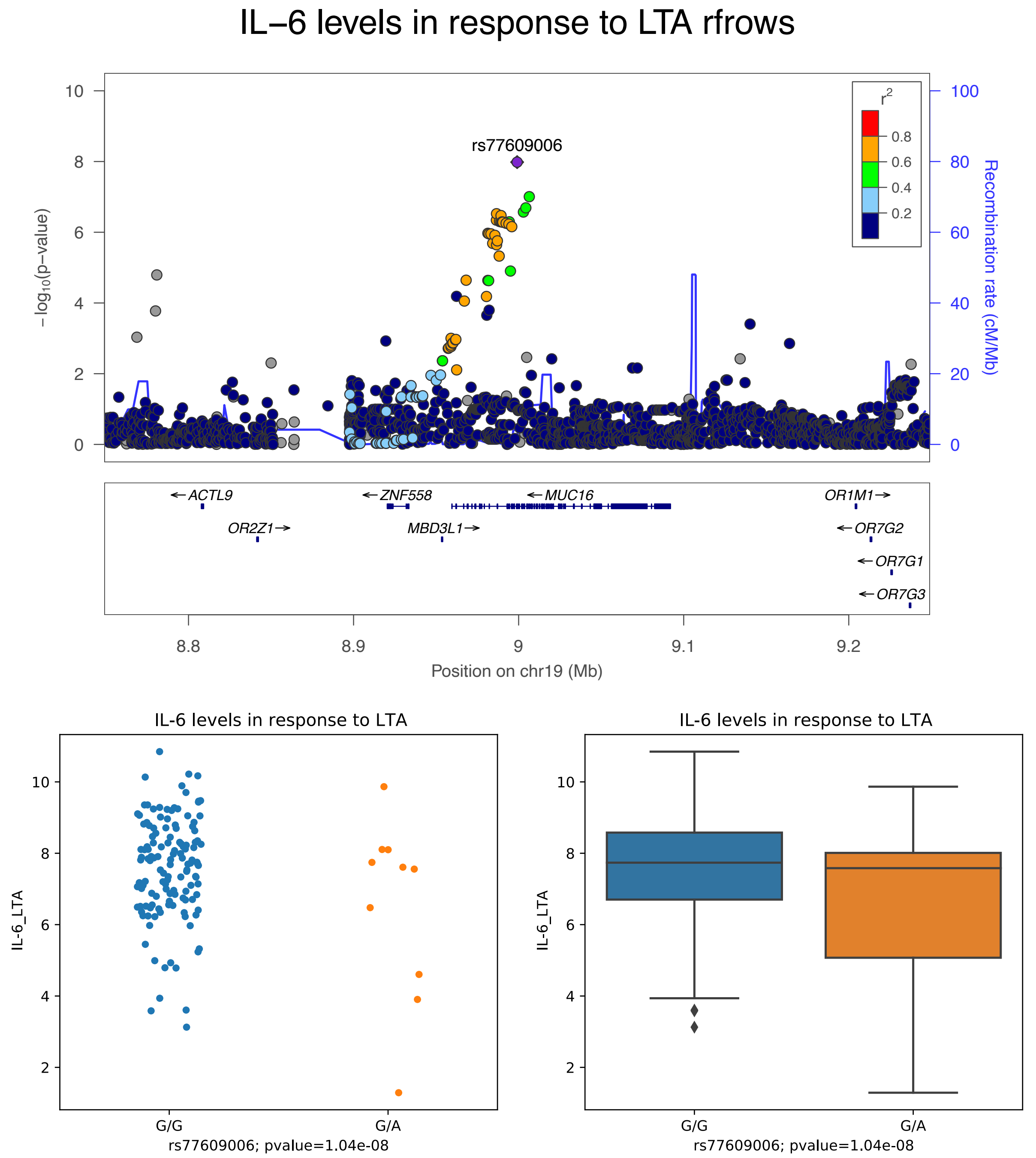

Fig. S8 Locuszoom Plots and IL-6 levels by genotypes for cQTL. (d) rs77609006 (associated with IL-6 levels in response to LTA).

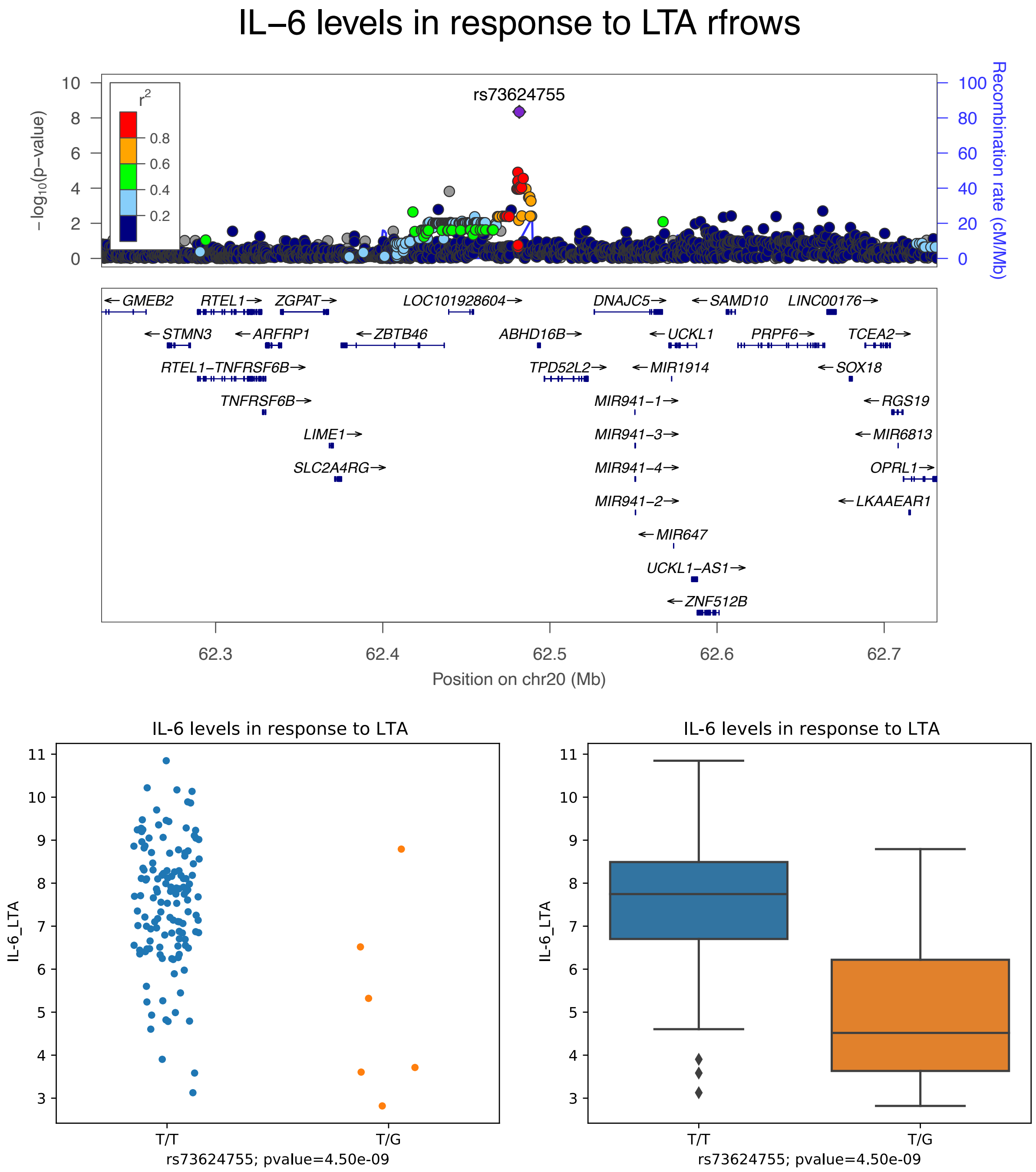

Fig. S8 Locuszoom Plots and IL-6 levels by genotypes for cQTL. (e) genotype rs73624755 (associated with IL-6 levels in response to LTA).

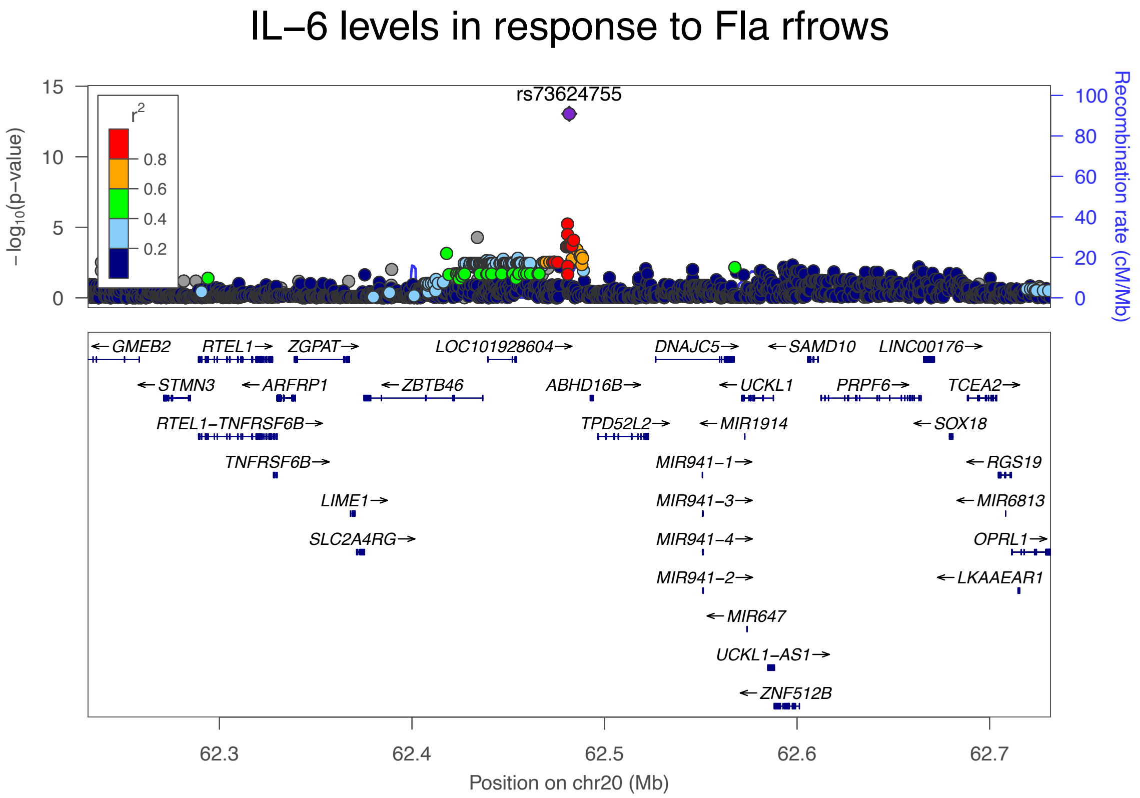

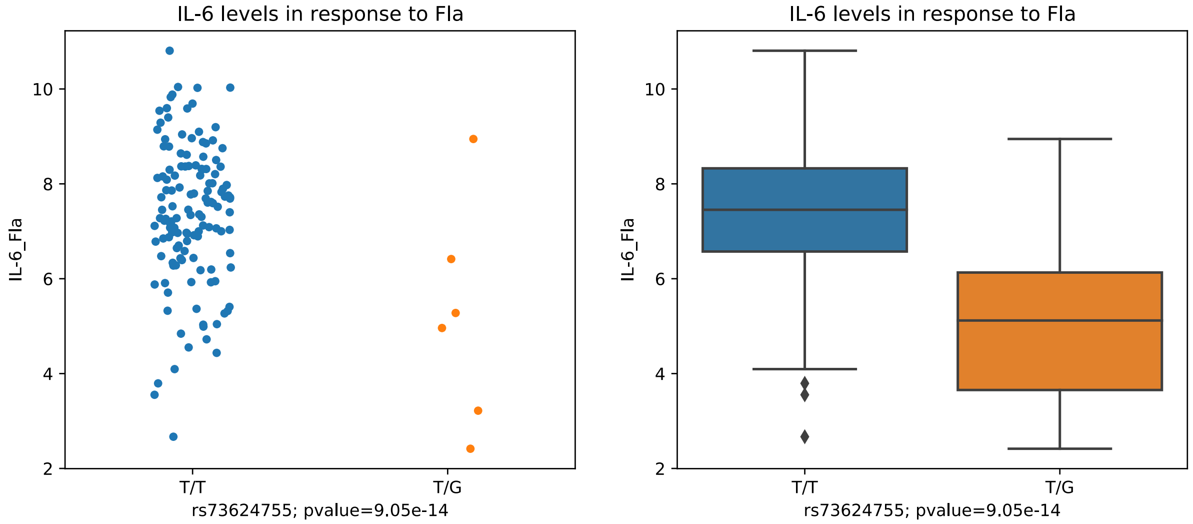

Fig. S8 Locuszoom Plots and IL-6 levels by genotypes for cQTL. (f) genotype rs73624755 (associated with IL-6 levels in response to Fla).

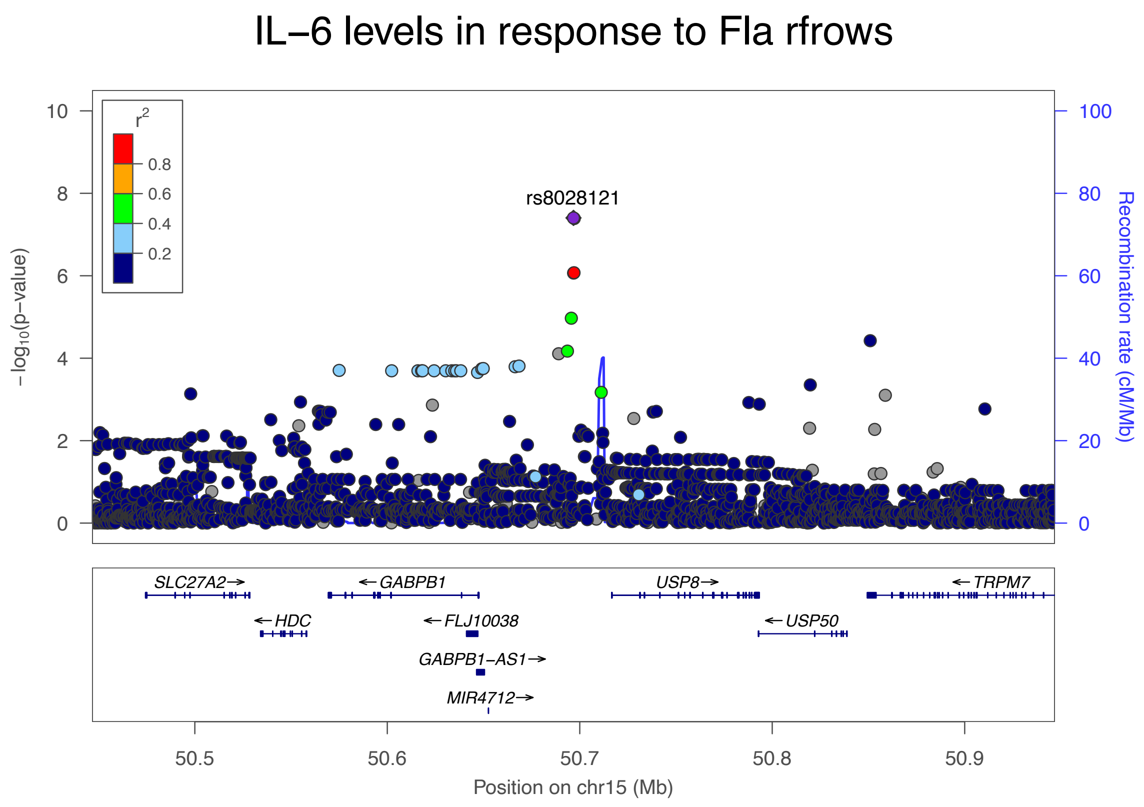

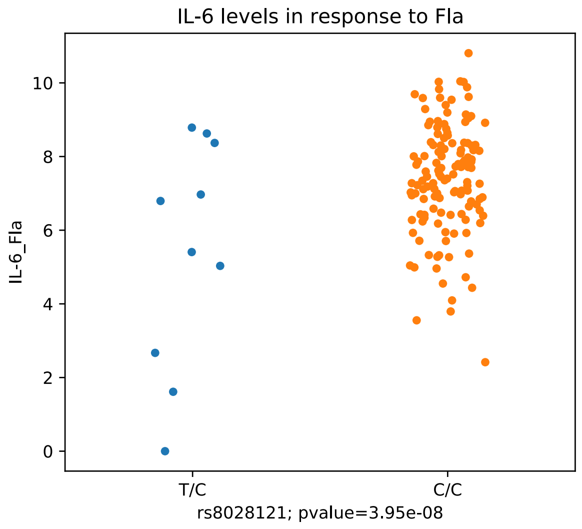

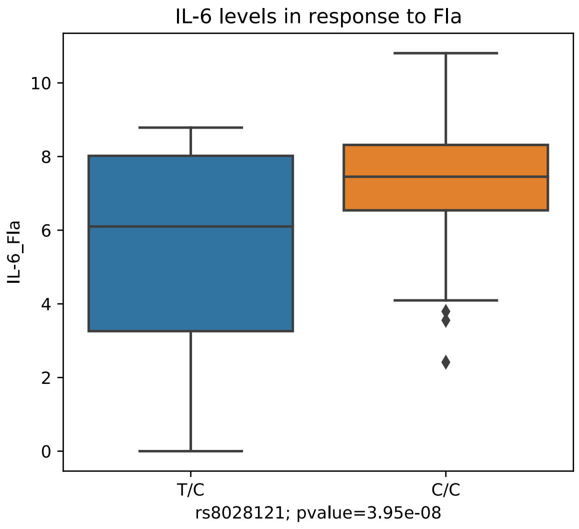

Fig. S8 Locuszoom Plots and IL-6 levels by genotypes for cQTL. (g) genotype rs8028121 (associated with IL-6 levels in response to Fla).

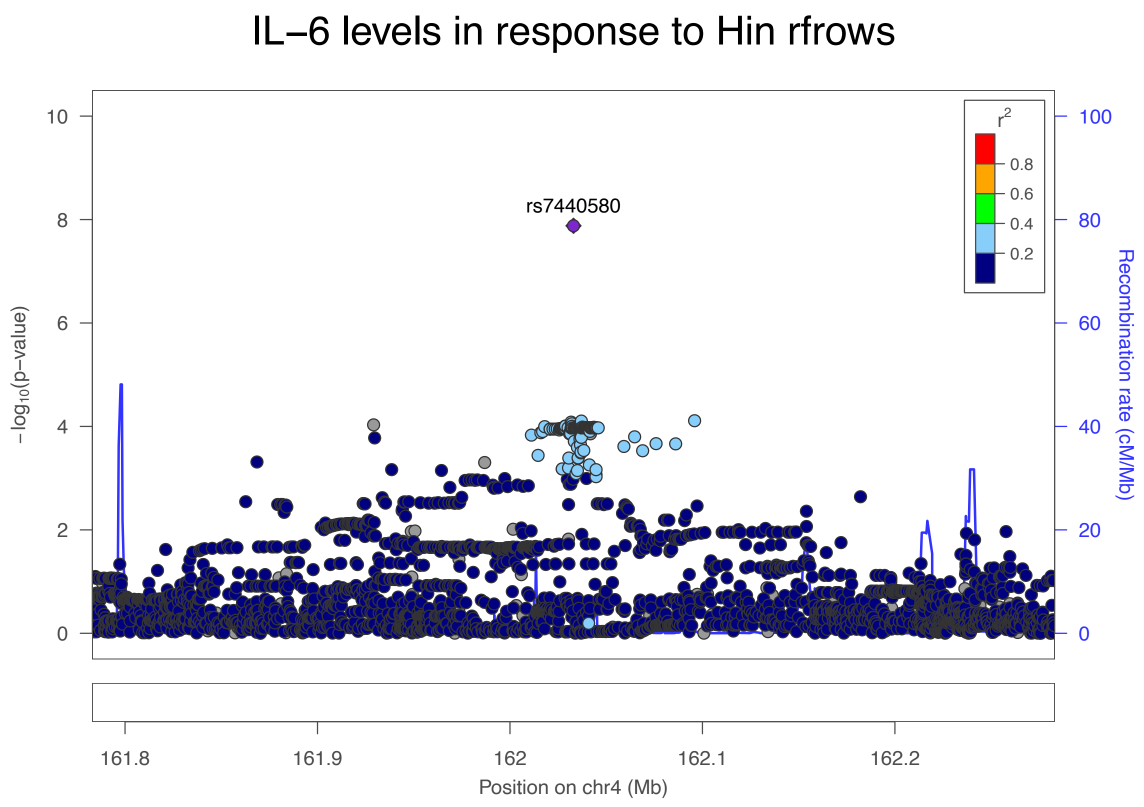

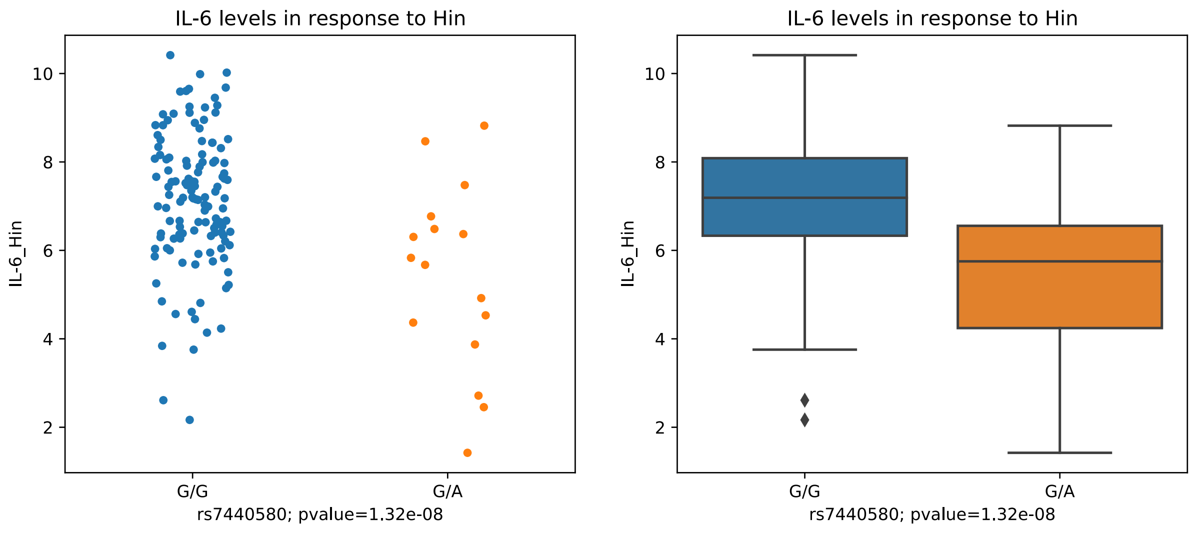

Fig. S8 Locuszoom Plots and IL-6 levels by genotypes for cQTL. (h) genotype rs7440580 (associated with IL-6 levels in response to Hin).

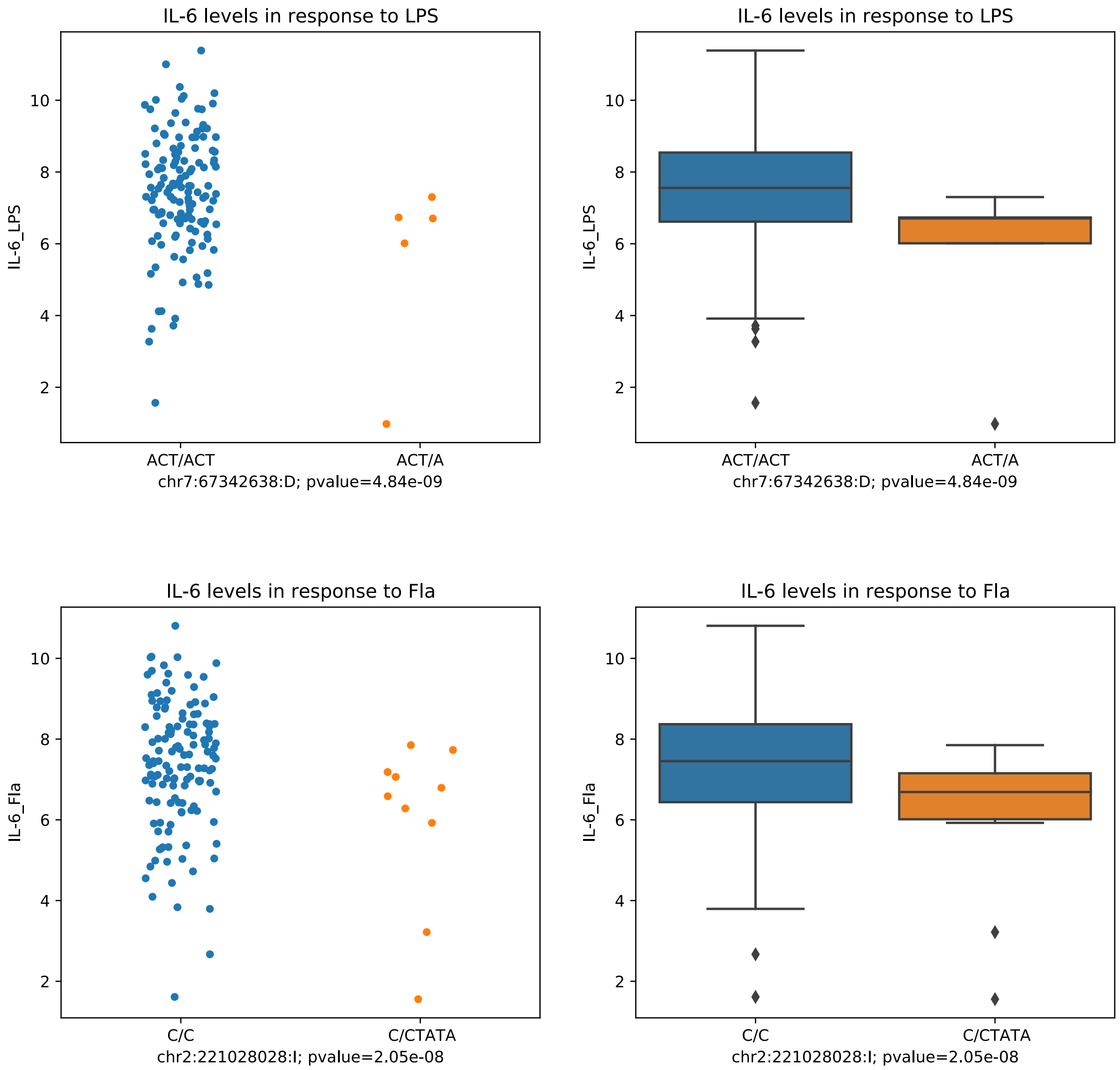

Fig. S8 Locuszoom Plots and IL-6 levels by genotypes for cQTL. (i) genotypes chr7:67342638:D and chr2:221028028:I (associated with IL-6 levels in response to FLS and Fla respectively). It was not possible to generate locuszoom plots for these two SNPs.
