# Supplementary material for: A systems immunology approach to investigate cytokine responses to viruses and bacteria and their association with disease
Source: Sci Rep. 2022 Aug 5;12:13463. doi: 10.1038/s41598-022-16509-4 (PMC9356009; doi:10.1038/s41598-022-16509-4)
Supplement: Supplementary file 5 — Supplementary Figure S9. [file 41598_2022_16509_MOESM5_ESM.pdf]

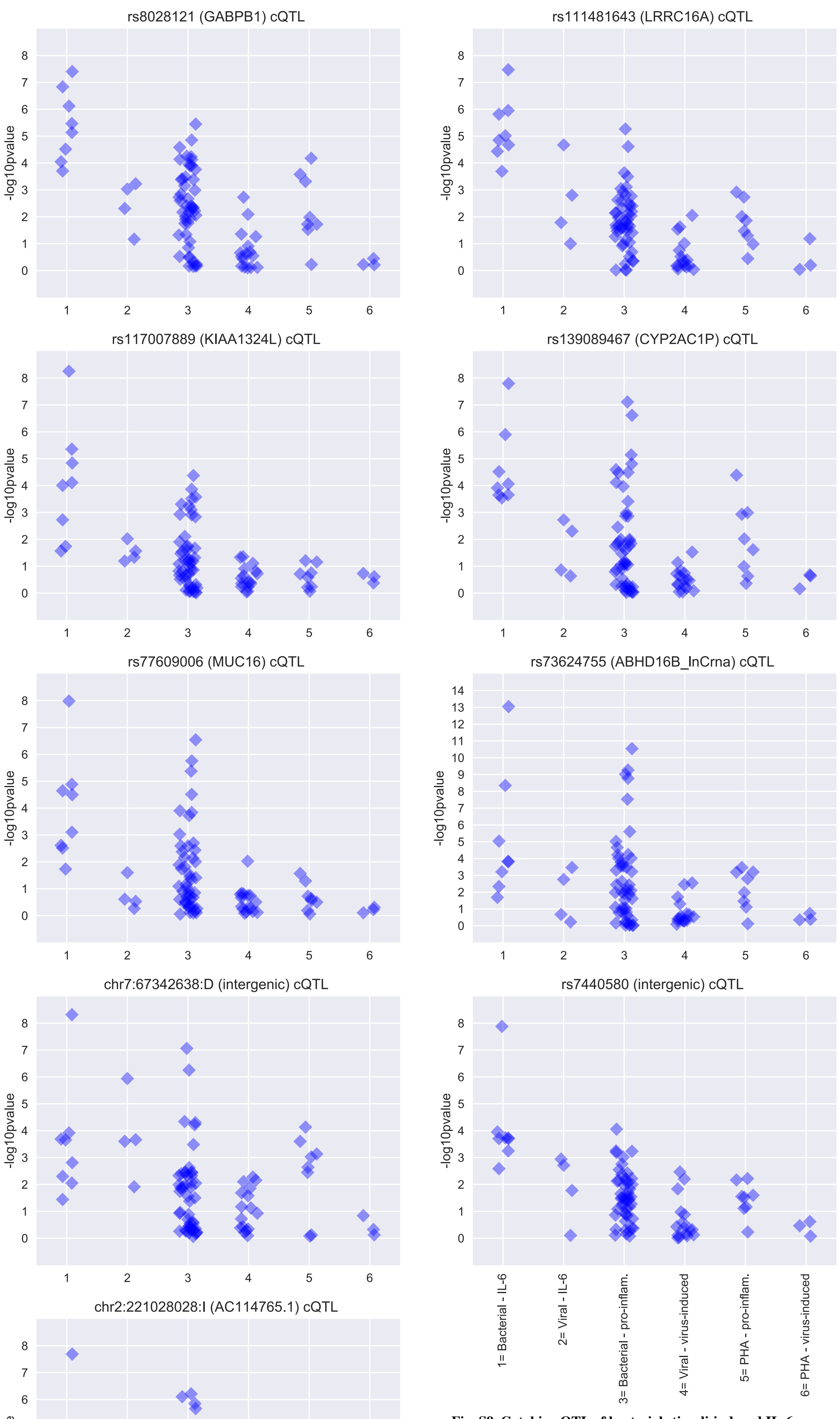

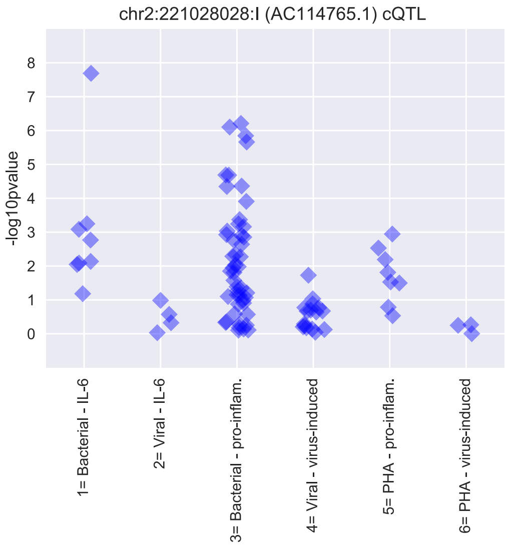

Fig. S9. Cytokine QTL of bacterial stimuli-induced IL-6 are associated with bacterial stimuli-induction of other pro-inflammatory cytokines.

Nine SNPs underlying ten cQTL were associated with IL-6 response to at least one bacterial stimulus (x-axis=1). We assessed if these cQTL were also associated with IL-6 levels in response to viral stimuli (x-axis=2), other pro-inflammatory cytokine responses to bacterial stimuli (x-axis=3), virus induced cytokine responses to viral stimuli (x-axis=4), PHA induced pro-inflammatory cytokine responses (x-axis=5), PHA induced virus-induced cytokine responses (x-axis=6). This figure was generated in Python (version 3.8, https://www.python.org/).
